# *C. elegans* germ granules require both assembly and localized regulators for mRNA repression

**DOI:** 10.1038/s41467-021-21278-1

**Published:** 2021-02-12

**Authors:** Scott Takeo Aoki, Tina R. Lynch, Sarah L. Crittenden, Craig A. Bingman, Marvin Wickens, Judith Kimble

**Affiliations:** 1 Department of Biochemistry and Molecular Biology, School of Medicine, Indiana University, Indianapolis, IN USA; 2 Department of Biochemistry, University of Wisconsin-Madison, Madison, WI USA; 3 Howard Hughes Medical Institute, University of Wisconsin-Madison, Madison, WI USA

**Keywords:** Biochemistry, RNA, Germline development, Gene regulation, RNA metabolism

## Abstract

Cytoplasmic RNA–protein (RNP) granules have diverse biophysical properties, from liquid to solid, and play enigmatic roles in RNA metabolism. Nematode P granules are paradigmatic liquid droplet granules and central to germ cell development. Here we analyze a key P granule scaffolding protein, PGL-1, to investigate the functional relationship between P granule assembly and function. Using a protein–RNA tethering assay, we find that reporter mRNA expression is repressed when recruited to PGL-1. We determine the crystal structure of the PGL-1 N-terminal region to 1.5 Å, discover its dimerization, and identify key residues at the dimer interface. Mutations of those interface residues prevent P granule assembly in vivo, de-repress PGL-1 tethered mRNA, and reduce fertility. Therefore, PGL-1 dimerization lies at the heart of both P granule assembly and function. Finally, we identify the P granule-associated Argonaute WAGO-1 as crucial for repression of PGL-1 tethered mRNA. We conclude that P granule function requires both assembly and localized regulators.

## Abstract (web summary)

Nematode P granules are cytoplasmic RNA–protein biomolecule condensates central to germ cell development. Here the authors show that dimerization of the PGL-1 scaffolding protein is crucial to granule formation and mRNA repression, and that the WAGO-1 Argonaute protein is a cofactor in repressing PGL-1 bound mRNAs.

## Introduction

RNA–protein (RNP) granules, otherwise known as biomolecular condensates^1^, are ubiquitous non-membrane-bound organelles. Some RNP granules exist in a solid-like state with little component exchange^2^, while others behave as liquid-like droplets with components dynamically diffusing in and out of the granule^3,4^. Intriguingly, mRNA regulators localizing to liquid granules do not rely on granule association for their activities^5^. Therefore, despite intense interest and an ever-expanding literature, the functional relationship between granule assembly and mRNA regulation is still unclear.

**Fig. 1:**
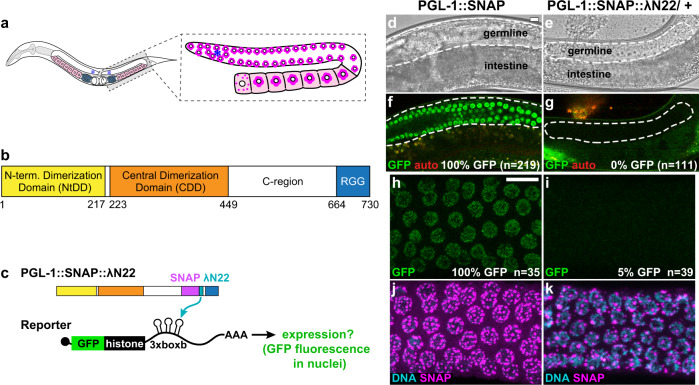
PGL-1 tethering represses an mRNA reporter in vivo. **a** Left, *C. elegans* adult hermaphrodite possesses two gonadal arms with proliferating germ cells at one end (asterisk) and differentiating gametes at the other. Gonads make sperm (blue) first and then oocytes (pink). Right, P granules (magenta) reside at the nuclear periphery of all germ cells until late oogenesis. Modified from^75^. **b** Linear diagram of *C. elegans* PGL-1. N-terminal dimerization domain (NtDD, yellow), central dimerization domain (CDD, orange), C-terminal region (C-region), and RGG repeats (blue). **c** Protein–mRNA tethering assay. The reporter mRNA encodes GFP (green)-histone H2B and harbors three boxB hairpins in its 3′UTR; a ubiquitous germline promoter drives expression (see Methods). λN22 peptide (light blue) is inserted into PGL-1 with a SNAP tag (magenta). Binding of PGL-1::SNAP::λN22 to boxB hairpins recruits reporter mRNA. Modified from^76^. **d**–**g** GFP reporter expression in germ cells of live animals. (**d**, **e**) Brightfield image. (**f**, **g**) GFP fluorescence (green); auto fluorescence (red). *n*, number of animals scored for GFP expression. %, germlines with detectable GFP. Scale bar, 10 μm, in (**d**) applies to (**d**–**g**). Fisher’s exact test of PGL-1::SNAP vs. PGL-1::SNAP::λN22/+ (*p*-value < 0.0001). **h**–**k** Representative images in fixed gonads. (**h**, **i**) GFP fluorescence (green). (**j**, **k**) SNAP staining (magenta) and DNA (DAPI, cyan). *n*, number of germlines scored for GFP expression. Fisher’s exact test of PGL-1::SNAP vs. PGL-1::SNAP::λN22/+ (*p*-value < 0.0001). Scale bar, 10 μm, in (**h**) applies to images. Scale bar, 10 µm. Figure 1 and Fig. 5 results were performed in parallel, and thus results from (**e**, **g**, **i**, **k**) are the same as reported in Fig. 5b, d, f, h.

Here, we investigate the functional relationship between RNP granule assembly and function in *Caenorhabditis elegans* germline P granules. P granules are paradigmatic RNP granules^6^ with striking similarities to germ granules in *Drosophila* and vertebrates, both in subcellular location and composition^7^. They are crucial for fertility and totipotency^8^, and some of their components can display liquid droplet behavior^9,–12^. In the primordial germ cells of the embryo, P granules are proposed to protect mRNAs from small RNA-based regulatory pathways^13,14^, and to localize and concentrate mRNAs for robust germline development independent of translational repression^15,16^. In adult germ cells, P granules are essential for proper germ cell development^17,18^. They localize to the cytoplasmic face of nuclear pores (Fig. 1a) and contain both untranslated mRNAs^19^ and numerous RNA regulatory proteins^20^. RNAi-mediated gene repression is seated in P granules^21^ and their loss correlates with aberrant upregulation of somatic transcripts^22–24^. The direct mechanistic connections between granule scaffold assembly, localized regulators, and mRNA regulation remain an open area of investigation, particularly in adult P granules. An improved understanding of this fundamental question requires manipulation of mRNA localization as well as manipulation of granule formation without eliminating pivotal assembly proteins. The former is made possible with a protein–mRNA tethering assay^25–27^, but the latter requires a deeper understanding of P granule scaffolding (PGL) proteins and the molecular basis of their assembly.

Two PGL proteins, PGL-1 and PGL-3, are key scaffolding proteins required for proper P granule assembly^17,18^. Genetic removal of both PGL proteins causes aberrant expression of spermatogenic and somatic mRNAs^22–24^, and sterility in a majority of worms^17,18^. Despite having high sequence identity (58.8% identity, 70.2% similarity^28^), the individual PGLs have distinct phenotypes and binding partners in the adult germline. The *pgl-1* null mutants are temperature-sensitive sterile, while *pgl-3* null mutants are fertile^18^. Moreover, PGL-1, not PGL-3, interacts with a germline-specific eIF4e ortholog^18^, and removal of PGL-1 prevents this eIF4e from localizing to P granules^29^. PGL proteins can self-assemble into granules, both in vitro using purified recombinant protein^9,11,12^ and in intestinal nematode cells or mammalian cells in culture when expressed on their own^30,31^. These in vitro PGL granules display liquid droplet behavior^9,11,12^, indicating that PGL protein alone is sufficient to recapitulate the biophysical properties of P granules in nematode cells^10^.

The molecular details of PGL assembly into granules were poorly understood though progress had been made. PGL-1 and PGL-3 are close paralogs^18^ with the same architecture (Fig. 1b). Many RNP granule assembly proteins rely on low-complexity sequences for multivalent–multivalent, low-affinity interactions^1^. The only low-complexity sequences in PGL are RGG repeats at the C-terminus, which mediate PGL RNA binding and are dispensable for granule formation^12,30^. Assays for granule assembly in mammalian cells implicated the conserved N-terminal region of PGL as critical^30^, and structural studies identified a central dimerization domain (DD) within that conserved region (Fig. 1b)^32^. Yet higher-ordered self-assembly demands additional PGL–PGL contacts.

In this work, we employ a tethering assay to manipulate mRNA localization in and out of granules, structural analyses to identify a new PGL dimerization domain, and incisive mutational intervention to discover the role of dimerization in P granule assembly and mRNA regulation. Our findings provide evidence that repression of mRNA expression in P granules requires both assembly and localized regulators and hence makes a major advance in understanding the functional relationship between RNP granule assembly and function.

## Results

### Tethered PGL-1 localizes to perinuclear granules and represses expression of the reporter mRNA

Prior studies have suggested that adult germ cell P granules regulate mRNA expression. To test this notion directly, we relied on a protein–mRNA tethering assay (Fig. 1c)^25,26^ widely used to investigate RNA regulatory proteins^27^. Our assay examined the expression of mRNAs to which the PGL-1 and PGL-3 proteins were tethered via λN22, a short peptide that binds with high affinity and specificity to the boxB RNA hairpin^25^. This assay has previously been used to identify the functions of a variety of RNA-binding proteins in several organisms, including nematodes^33^. For the reporter, we inserted three boxBs into the 3′UTR of an established GFP-histone transgene that is ubiquitously expressed throughout the germline^34^ (Fig. 1c, Methods). To tether PGL-1 to the GFP reporter mRNA via boxB, we generated PGL-1::SNAP::λN22 with sequential CRISPR/Cas9 gene editing in an internal, non-conserved protein region of PGL-1 (Fig. 1c and Supplementary Fig. 1a, see Methods). The SNAP tag^35^ is used to visualize subcellular localization and λN22 provides tethering. PGL-1::SNAP::λN22 homozygotes were sterile (100%, *n* = 94), but could be maintained and tested as a fertile heterozygote (PGL-1::SNAP::λN22/+). Given that *pgl-1* null homozygotes are fertile^17^, the fertility defects from the addition of λN22 to PGL-1 is likely not due to defective protein function. The endogenous locus of PGL-3 was also modified by CRISPR/Cas9 to include 3xFLAG without or with λN22 (Supplementary Fig. 1b). The logic of our tethering strategy is simple: if tethered PGL localizes to granules and represses GFP expression as predicted, this assay provides a powerful entrée into fundamental questions about granule function.

To evaluate mRNA regulation, we assayed reporter GFP fluorescence in both living animals and fixed, extruded gonads; the former facilitated scoring many samples and the latter permitted scoring subcellular localization of PGL proteins via staining and *gfp* RNA with single molecule fluorescence in situ hybridization (smFISH). In controls carrying PGL-1::SNAP without λN22, GFP fluorescence was robust (Fig. 1d, f), but GFP fluorescence was absent in animals with PGL-1::SNAP::λN22 (Fig. 1e, g). Essentially the same result was found in fixed germlines: all germlines carrying PGL-1::SNAP without λN22 expressed GFP (Fig. 1h and Supplementary Fig. 2c, n), but GFP signal was absent from most PGL-1::SNAP::λN22 germlines (Fig. 1i and Supplementary Fig. 2g, n). Importantly, the PGL-1::SNAP and PGL-1::SNAP::λN22 proteins both assembled into cytoplasmic granules at the nuclear periphery (Fig. 1j, k and Supplementary Fig. 2d, h), similar to untagged PGL-1 and PGL-3 reported previously^17,18^. The SNAP signal was lower for PGL-1::SNAP::λN22, perhaps because animals were heterozygous. In contrast with repression seen with tethered PGL-1, PGL-3 tethered to the reporter RNA did not affect reporter expression (Supplementary Fig. 3). As noted in the Introduction, *pgl-1* and *pgl-3* mutants have distinct phenotypes, and PGL-1 and PGL-3 proteins interact with different binding partners^18^. Our tethering assays now suggest a mechanistic difference between these two scaffold proteins in their ability to regulate associated transcripts. We conclude that tethered PGL-1 localizes to perinuclear granules and represses expression of the reporter mRNA.

### PGL-1 promotes mRNA recruitment to perinuclear granules

We next asked if the reporter RNA localizes with PGL-1 in perinuclear granules. To this end, we used smFISH to detect *gfp* RNAs and SNAP staining to detect PGL-1 (Supplementary Fig. 2a). Control germ cells expressing PGL-1::SNAP without λN22 possessed *gfp* RNAs in both nuclear and cytoplasmic puncta (Supplementary Fig. 2b–e). We interpret nuclear puncta as nascent transcripts at active transcription sites and cytoplasmic puncta as mRNAs, based on a previous study^36^. GFP fluorescence was robust (Supplementary Fig. 2c, n), and PGL-1::SNAP localized to perinuclear granules (Supplementary Fig. 2d), as in Fig. 1. Germ cells expressing PGL-1::SNAP::λN22 lacked robust GFP fluorescence (Supplementary Fig. 2g, n) and PGL-1::SNAP::λN22 localized to perinuclear granules (Supplementary Fig. 2h), also as in Fig. 1. The cytoplasmic RNA puncta were less diffuse with tethered PGL-1 than in the control and frequently colocalized with PGL-1::SNAP::λN22 in perinuclear granules (Supplementary Fig. 2f–i, o; see Supplementary Fig. 4 for additional images). Imaging of the rachis, the shared cytoplasmic space in the germline, did not reveal substantial aggregates of reporter RNA as observed with mRNA when regulation is disrupted in other studies^37^. The presence of *gfp* cytoplasmic transcripts (Supplementary Fig. 2f) demonstrates that the reporter was not transcriptionally silenced, despite the lack of GFP fluorescence. Total smFISH signal was lower in PGL-1::SNAP with λN22 worm germlines (Supplementary Fig. 2p), suggesting that tethered PGL-1 promotes mRNA turnover.

Although reporter mRNAs were clearly recruited to perinuclear granules, their recruitment was incomplete. One possible explanation is that PGL protein can easily dissipate from P granules^38^, and therefore partial fixation may lead to release of some P granule proteins and mRNAs into the cytoplasm. A second possible explanation relies on PGL granules being fluid, liquid droplets in cells^10^, which can allow PGL-1 and associated factors to diffuse in and out of P granules. Third, every reporter mRNA may not be bound to PGL-1. We also note that a few reporter mRNAs seemed to localize to granules in the absence of tethering. P granules are proposed to reside on the cytoplasmic side of most, if not all, nuclear pores^39^, and so these untethered reporter mRNAs may be newly transcribed mRNAs passing through P granules during transport. Alternatively, there may be endogenous mechanisms recruiting or trapping reporter mRNAs in P granules. Regardless, more reporter mRNAs were recruited to perinuclear granules after PGL-1 tethering.

### Structure of the PGL-1 N-terminal dimerization domain

To test the significance of P granule assembly to mRNA repression, we sought to perturb PGL-1 assembly into granules. An emerging principle is that multivalent–multivalent interactions drive granule formation (e.g., protein with at least two multimerization domains)^1,40^. Because PGLs are key assembly proteins for P granules and can form granules on their own^9,11,12,30,31^, we reasoned that PGLs use multiple self-interactions to drive granule assembly. We previously identified one dimerization domain (DD) centrally in PGL^32^, but DD missense mutations grossly affected protein stability. We postulated the existence of another PGL multimerization domain that might be more amenable to manipulation and again turned to structural analyses.

**Fig. 2:**
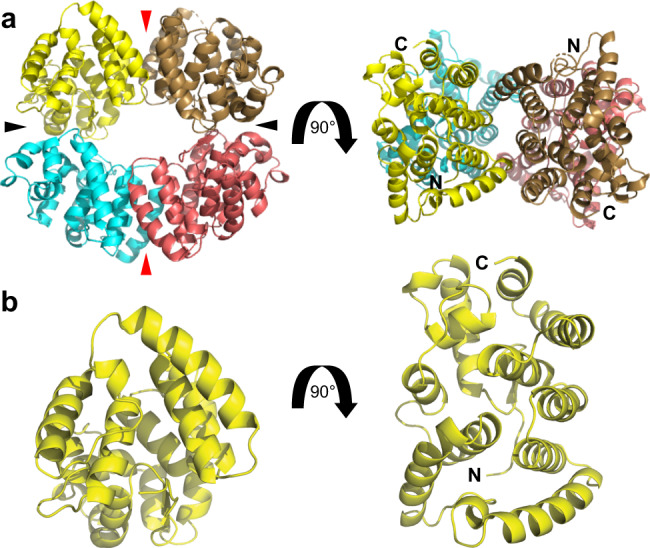
Crystal structure of PGL-1 NtDD. **a** Crystal structure of *C. japonica* PGL-1 NtDD to 1.5 Å. See Supplementary Table 1 for crystal structure data and model statistics. NtDD has four copies per asymmetric unit (ASU). Copies in yellow, brown, cyan, and salmon. Arrows indicate two pairs of subunit interfaces in the ASU. Red arrows highlight the interface relying on conserved amino acids (see text). Red arrow: interface buried surface area average, 874.1 Å^2^. Black arrow: interface buried surface area average, 572.2 Å^2^. Area calculated with PISA^77^. **b** Enlarged image of a single NtDD.

**Fig. 3:**
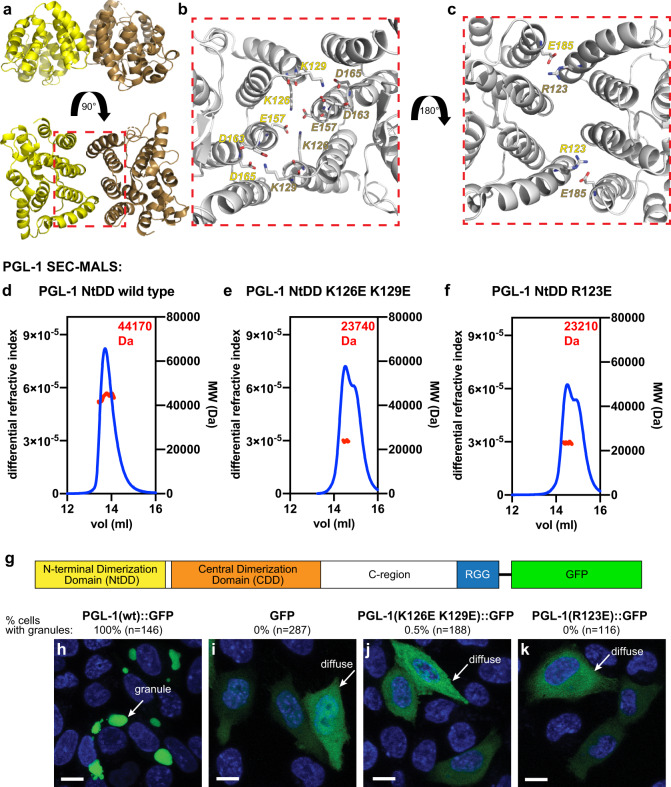
NtDD dimerization and its role in PGL-1 self-assembly. **a** Structural model of the NtDD dimer. Subunits in yellow and brown. **b**, **c** Enlargement of dimer interface (red box in **a**). PGL-1 amino acids (**b**) K126 and K129, and (**c**) R123 interact with apposing subunit side chains. Residue labels in yellow or brown to indicate their representative subunits. **d**–**f** Size exclusion chromatography and multi-angle light scattering (SEC-MALS) of recombinant PGL-1 NtDD (**d**) wild-type (44,170 (±6.509%) Da), (**e**) K126E K129E (23,740 (±2.455%) Da), and (**f**) R123E (23,210 (±2.099%) Da) proteins. Differential refractive index (left *y*-axis) in arbitrary units (blue). Molecular weight (MW, right *y*-axis) in dalton (Da, red). Wild-type protein measured the approximate size of a dimer, while both mutant proteins measured approximately as monomers. BSA control protein analyzed by SEC-MALS in Supplementary Fig. 5g. **g** Diagram of *C. elegans* PGL-1 C-terminally tagged with GFP. N-terminal dimerization domain (NtDD, yellow), central dimerization domain (CDD, orange), C-terminal region (C-region), RGG repeats (blue), and GFP (green). **h**–**k** Representative images of (**h**) GFP-tagged PGL-1, (**i**) GFP alone, and GFP-tagged PGL-1, (**j**) K126E K129E, and (**k**) R123E mutants expressed in Chinese Hamster Ovary (CHO) cells. Cell cultures were imaged live, and GFP-positive cells counted for the presence or absence of granules. Images show the majority result (percentages noted above image). Fisher’s exact test versus GFP: PGL-1 GFP (*p*-value < 0.0001), PGL-1 K126E K129E (*p*-value = 0.3958), PGL-1 R123E (>0.9999). Hoechst (DNA) in blue. GFP in green. Scale bar, 10 µm.

The region N-terminal to DD has high sequence conservation (Supplementary Fig. 1c), which implies a critical role in PGL function. Our initial efforts to express trypsin-mapped recombinant protein fragments^32^ of this N-terminal region proved unfruitful. However, addition of amino acids disordered in the DD crystal structures^32^ permitted robust expression sufficient for biochemical and structural characterization (Supplementary Fig. 5a–c, see Methods for more details). Henceforth, we refer to this stable protein fragment as the N-terminal dimerization domain (NtDD) (Fig. 1b). We determined the *Caenorhabditis japonica* PGL-1 NtDD crystal structure to 1.5 Å (Fig. 2 and Supplementary Table 1). The NtDD has a novel fold consisting of 11 alpha helices and a single N-terminal beta strand (Fig. 2b). The asymmetric unit (ASU) was composed of four NtDD domains (Fig. 2a), which were structurally similar (RMSD 0.219–0.254, C-alpha chains B-D aligned to C-alpha A) except amino acid G114, and the first three N-terminal residues could not be visualized in all of the copies of the ASU. While each ASU possessed two pairs of identical interfaces (Fig. 2a), one of these interface pairs consisted of a network of conserved amino acid side chains making extensive salt bridges and hydrogen bonds (Fig. 3a–c and Supplementary Fig. 5d–f). The complexity and conservation of these interactions suggested biological relevance. We next tested for dimerization in vitro. Recombinant PGL-1 and PGL-3 NtDD each dimerized on a sizing column combined with multi-angle light scattering (SEC-MALS, Fig. 3d and Supplementary Fig. 5g–i). To ask if the conserved interface in the NtDD crystal structure might be its dimerization interface, we used our structural model and in silico prediction^41^ to design missense mutations predicted to disrupt the interface. These analyses yielded two distinct mutants: R123E with a single mutated residue and K126E K129E with two mutated residues. PGL-1 and PGL-3 NtDD interface mutants formed monomers rather than dimers in solution (Fig. 3e, f and Supplementary Fig. 5h, i). We conclude that the dimers observed in the crystal structure represent the NtDD dimer detected in solution. Therefore, PGL proteins possess two dimerization domains. We renamed the original DD to Central DD (CDD) for clarity (Fig. 1b).

### PGL-1 dimerization through the NtDD is required for granule formation

To assess the role of NtDD dimerization in PGL granule self-assembly, we used an assay in mammalian cells where expression of wild-type PGL-1 tagged with GST was sufficient for assembly into granules^30^. Similar to that report, wild-type PGL-1 tagged with GFP also formed large cytoplasmic granules when expressed in mammalian cells (Fig. 3g, h), while GFP alone was diffuse (Fig. 3i). However, PGL-1::GFP mutated to either K126E K129E or R123E no longer self-assembled into granules (Fig. 3j, k). We conclude that PGL-1 NtDD dimerization is essential for granule formation in mammalian cells.

**Fig. 4:**
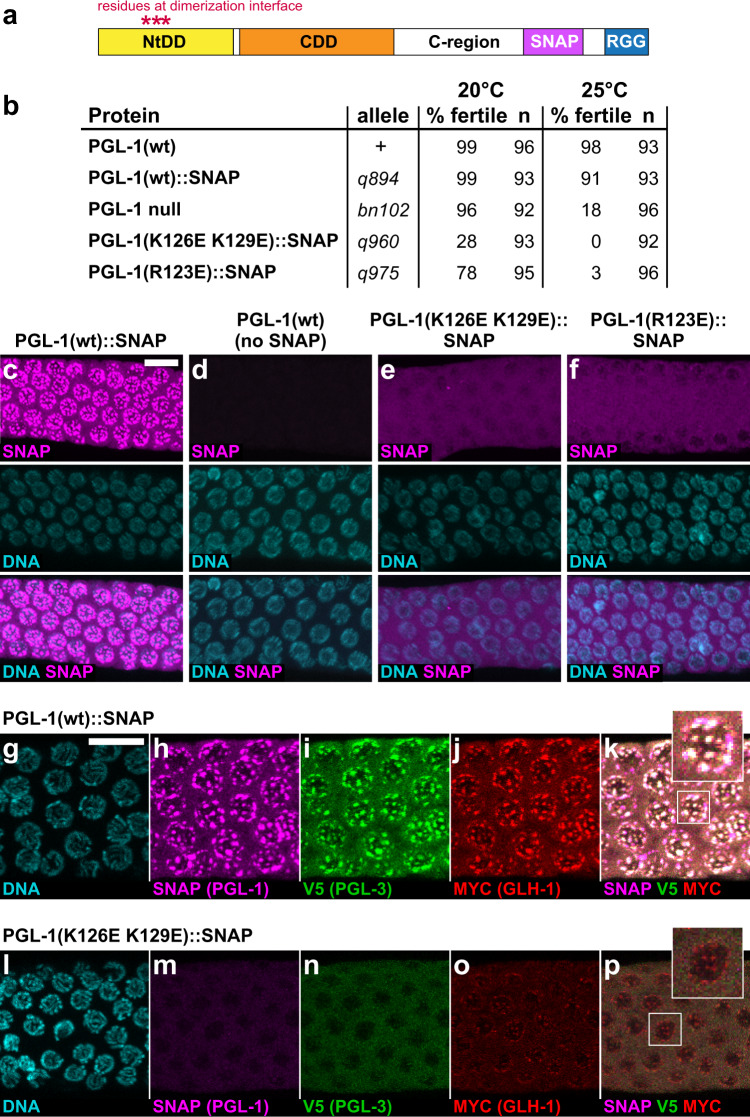
NtDD dimerization is critical for fertility and P granule formation in nematodes. **a** Sites of SNAP tag insertion and missense mutations in *C. elegans* PGL-1. N-terminal dimerization domain (NtDD, yellow), central dimerization domain (CDD, orange), C-terminal region (C-region), SNAP (magenta), and RGG repeats (blue). **b** Fertility of SNAP-tagged PGL-1 animals. Percentages were obtained after scoring individuals for production of larval progeny after 5 days at either 20 or 25 °C. Statistics reported in Supplementary Fig. 6a. **c**–**p** Extruded adult germlines, fixed, stained, and imaged in same region of meiotic pachytene (see Supplementary Fig. 6b). (**c**–**f**) Representative images of SNAP staining to visualize PGL-1 expression and granule formation. SNAP in magenta, DNA (DAPI) in cyan. All images are partial Z-stacks to maximize visualization of P granules. Images were taken from germlines containing embryos; similar images were obtained from germlines lacking embryos (Supplementary Fig. 6f, g). (**c**) PGL-1::SNAP localizes to granules around nuclei (*n* = 49). (**d**) Control, wild-type animal lacking SNAP tag shows virtually no background staining (*n* = 20). (**e**) PGL-1(K126E K129E)::SNAP is diffuse (*n* = 38). (**f**) PGL-1(R123E)::SNAP is diffuse (*n* = 24). (**g**–**p**) Representative images showing localization of three P granule components in germ cells expressing either (**g**–**k**) PGL-1::SNAP (*n* = 20) or (**l**–**p**) PGL-1(K126E K129E)::SNAP (*n* = 14). (**g**, **l**) DNA (DAPI, cyan); (**h**, **m**) SNAP (PGL-1::SNAP or mutant, magenta); (**i, n**) V5 (PGL-3, green); (**j**, **o**) MYC (GLH-1, red); (**k**, **p**) Merge. Scale bar, 10 µm for all images, except 2.5-fold enlargements of nuclei in boxes placed outside main images. *n* = biologically independent animals examined over 2 independent experiments.

To assess the role of PGL-1 NtDD dimerization in nematode germ cells, we used CRISPR/Cas9 to introduce the dimerization-defective mutations into SNAP-tagged PGL-1 (Fig. 4a and Supplementary Fig. 1a, see Methods). We first asked about effects on fertility (Fig. 4b and Supplementary Fig. 6a). Most wild-type PGL-1 (no SNAP) and PGL-1::SNAP animals were fertile at 20 and 25 °C (Fig. 4b and Supplementary Fig. 6a). In contrast, most *pgl-1* null mutants were fertile at 20 °C but few were fertile at 25 °C (Fig. 4b and Supplementary Fig. 6a), as reported previously^17,18^. Fertility of the PGL-1 NtDD dimerization mutants, K126E K129E and R123E, by contrast, was sharply reduced at both 20 and 25 °C; many mutant worms were sterile at 20 °C and most were sterile at 25 °C (Fig. 4b and Supplementary Fig. 6a). Fertility was therefore impacted more severely in dimerization mutants than in *pgl-1* null mutants (Fig. 4b and Supplementary Fig. 6a). The NtDD dimerization mutants had smaller than normal germlines and many lacked oocytes (Supplementary Fig. 6c–e), which are defects typical of *pgl-1* and *pgl-1 pgl-3* null mutants^17,18^. We conclude that PGL-1 NtDD dimerization is critical for fertility. The sterility of the PGL-1 dimerization mutants compared to null animals suggests that NtDD dimerization mutation disrupts the function of proteins other than PGL-1.

To investigate the role of PGL-1 NtDD dimerization in granule assembly, we compared the subcellular localization of wild-type PGL-1::SNAP to NtDD dimerization-defective PGL-1::SNAP mutant proteins (Fig. 4c–f). Wild-type PGL-1::SNAP assembled into cytoplasmic granules at the nuclear periphery (Fig. 4c), but the K126E K129E and R123E mutant proteins were largely diffuse in both fertile (Fig. 4e, f) and sterile (Supplementary Fig. 6f, g) germlines. The two mutant PGL-1 fluorescent intensities were equivalent and above background (Supplementary Fig. 7a–g), albeit less than wild-type PGL-1. Immunoblots also revealed a decrease in protein expression between PGL-1 wild-type and mutant protein (Supplementary Fig. 7h). While there are potential artifacts in quantitating levels of granule versus cytoplasmic proteins, there exists the possibility that mutant PGL-1 protein has lower protein levels due to higher protein turnover rates^11,42,43^. Changes in protein abundance may affect the propensity of PGL-1 to form granules. Of note, the mutant PGL-1s were capable of forming small perinuclear granules in a variable number of germ cells (Fig. 4e, f and Supplementary Fig. 6f, g). In addition, for each mutant, we found a single germline (1 of 59 for K126E K129E; 1 of 54 for R123E) with PGL-1 perinuclear granules in all germ cells (Supplementary Fig. 6h, i). Therefore, both PGL-1 mutant proteins are capable of incorporating into granules, but do so much less efficiently than their wild-type counterparts (Fig. 4c).

We next asked why the fertility defects of PGL-1 NtDD dimerization mutants were more severe than a *pgl-1* null mutant. The likely explanation was interference with assembly of other P granule components into granules. Normally, PGL-1 interacts with PGL-3^18^, and both PGL-1 and PGL-3 rely on GLH-1 or GLH-4 Vasa helicases to localize to the nuclear periphery in adult germ cells^31,44^. In contrast, GLH proteins can assemble at the nuclear pore independently of PGLs^18^. We postulated that PGL-1 assembly mutants might interfere with PGL-3 assembly into granules but not affect GLH-1. To test this idea, we epitope-tagged endogenous *pgl-3* and *glh-1* and compared localization of PGL-3::V5 and GLH-1::Myc in germ cells also expressing either wild-type PGL-1::SNAP or the dimerization-defective mutant PGL-1(K126E K129E)::SNAP. In the presence of wild-type PGL-1::SNAP, all three proteins, PGL-1, PGL-3 and GLH-1, colocalized to granules at the nuclear periphery (Fig. 4g–k), as previously observed for untagged proteins^17,18,45^. By contrast, a dimerization-defective mutant protein, PGL-1(K126E K129E)::SNAP, was diffuse rather than granular, and wild-type PGL-3 became similarly diffuse (Fig. 4l–n). GLH-1, however, was still capable of localizing to granules at the nuclear periphery (Fig. 4o–p), similar to previous reports^18^. Although these GLH-1 granules appeared smaller than normal, their formation was seen around virtually all germline nuclei (Fig. 4p). We conclude that the mutation-induced defect in PGL-1 NtDD dimerization has a dominant-negative effect that reduces assembly of both PGL-1 and PGL-3 into granules, and that this likely explains the severe fertility defects of PGL-1 dimerization mutants.

**Fig. 5:**
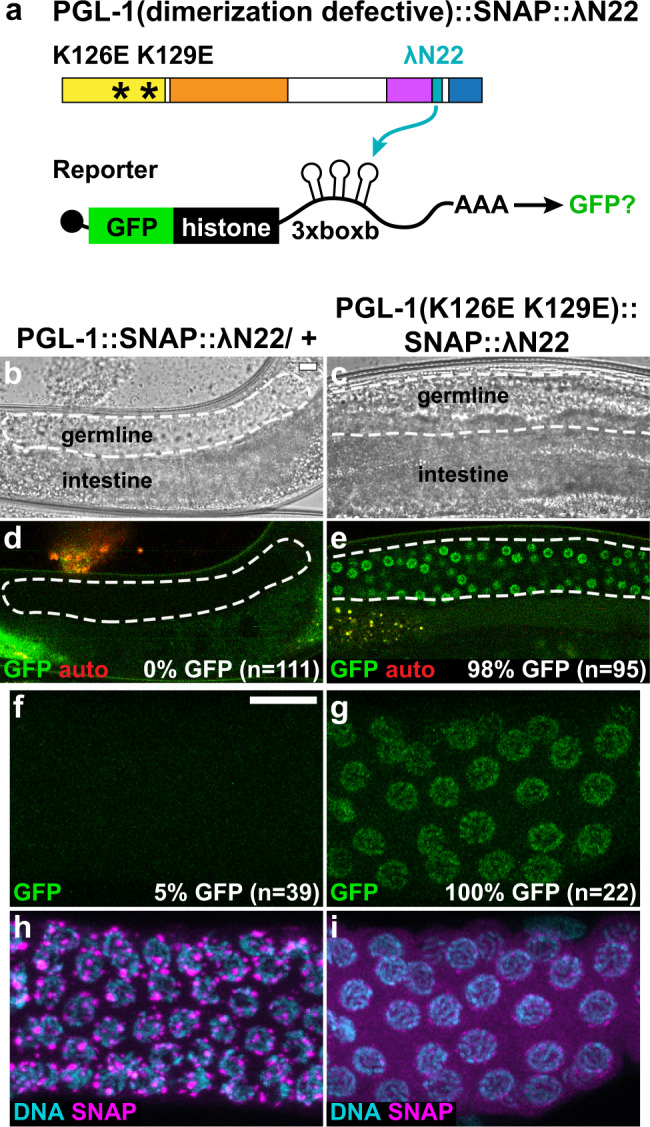
PGL-1 assembly is required for tethered mRNA reporter repression. **a** Protein–mRNA tethering assay and PGL-1 assembly. To test the necessity of granule formation for mRNA repression, NtDD assembly mutations were added to PGL-1::SNAP::λN22 and germlines observed for GFP reporter expression. N-terminal dimerization domain (NtDD, yellow), central dimerization domain (CDD, orange), SNAP (magenta), λN22 (light blue) and RGG repeats (blue), and GFP (green). Modified from^76^. **b**–**e** GFP reporter expression in germ cells of live animals. (**b**, **c**) Brightfield image. (**d**, **e**) GFP fluorescence (green); auto fluorescence (red). *n*, number of animals scored for GFP expression. Scale bar, 10 μm, in (**b**) applies to (**b**–**e**) images. **f**–**i** Representative images of PGL-1 granule formation, seen by SNAP staining (magenta) and GFP fluorescence (green) in fixed gonads. DNA (DAPI) in cyan. *n*, number of germlines scored for GFP expression. %, germlines with detectable GFP. Fisher’s exact test of PGL-1::SNAP::λN22 vs. PGL-1 (K126E K129E)::SNAP::λN22 (*p*-value < 0.0001). Scale bar, 10 μm, in (**f**) applies to (**f**–**i**) images. Figure 1 and Fig. 5 results were performed in parallel, and thus results from (**b**, **d**, **f**, **h**) are the same as reported in Fig. 1e, g, i, k.

The identification of assembly-defective PGL-1 proteins coupled with our tethering assay (Fig. 5a) allowed us to test the relationship between granule assembly and mRNA repression. We introduced the K126E K129E mutation into PGL-1::SNAP::λN22 and tested for reporter expression. PGL-1(K126E K129E)::SNAP::λN22 homozygotes were fertile (21%, *n* = 96) to an extent comparable to PGL-1(K126E K129E)::SNAP without λN22 (Fig. 4b and Supplementary Fig. 6a). While control PGL-1::SNAP::λN22 repressed the reporter (Fig. 5d, f), assembly-defective PGL-1 was not repressive and the vast majority of germ cells expressed GFP (Fig. 5e, g and Supplementary Fig. 2k, n). The PGL-1(K126E K129E)::SNAP::λN22 mutant protein was diffuse and non-granular compared to wild-type PGL-1::SNAP (Fig. 5h, i), similar to PGL-1(K126E K129E)::SNAP without λN22 (Fig. 4e). By smFISH, *gfp* RNA signal in PGL-1(K126E K129E)::SNAP::λN22 mutant germlines was observed robustly throughout the cytoplasm (Supplementary Fig. 2j–m). Formally, the PGL-1 interface residues might affect assembly and mRNA repression independently, but the simplest explanation is that PGL-1 must assemble properly to repress mRNA.

### PGL-1 and WAGO-1 can assemble into granules independently of each other

PGL-1 tethering provides a simple assay for identification of additional factors needed for P granule mRNA repression (Supplementary Fig. 8a). We depleted candidate P granule-associated RNA regulators with RNAi and sought GFP reporter de-repression in PGL-1::SNAP::λN22 worms. In this candidate screen, knockdown of the cytoplasmic Argonaute WAGO-1 had a dramatic effect (Supplementary Fig. 8b). RNAi against other candidates, by contrast, had either no or a minor effect on GFP repression (Supplementary Fig. 8b). The RNAi screen highlighted WAGO-1 as a key factor in PGL-1 mediated mRNA repression, a finding consistent with previous studies showing that WAGO-1 localizes to P granules and regulates gene expression^46,47^.

**Fig. 6:**
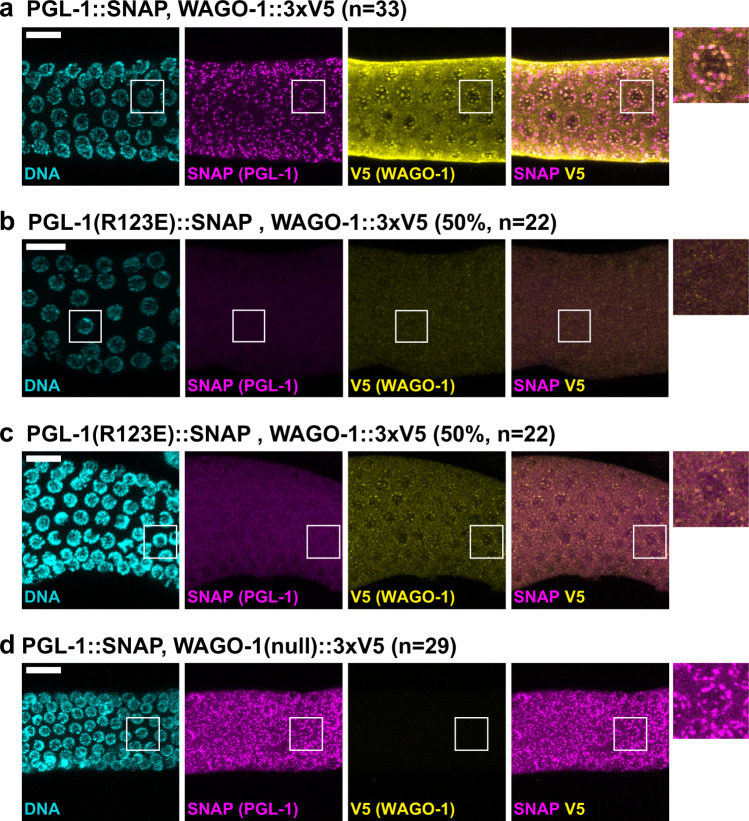
PGL-1 and WAGO-1 assemble independently in P granules. **a**–**d** Representative images showing localization of PGL-1 and WAGO-1 in germ cells expressing (**a**) PGL-1::SNAP, WAGO-1::3xV5 (*n* = 33). (**b**) PGL-1(R123E)::SNAP, WAGO-1::3xV5 without WAGO-1 puncta (11 of 22 germlines). (**c**) PGL-1(R123E)::SNAP, WAGO-1::3xV5 with WAGO-1 puncta (11 of 22 germlines). (**d**) PGL-1::SNAP, WAGO-1(null)::3xV5 (*n* = 29). DNA (DAPI, cyan); SNAP (PGL-1::SNAP or mutant, magenta); V5 (WAGO-1::3xV5 wild-type or null, yellow). Scale bar, 10 µm for all images, except 2.5-fold enlargements placed outside main images. *n* = biologically independent animals examined over 2 independent experiments.

To further investigate *wago-1*, we first inserted an epitope-tag at the endogenous locus (Supplementary Fig. 8c, see Methods). WAGO-1::3xV5 colocalized with PGL-1(wt)::SNAP in perinuclear granules (Fig. 6a), consistent with a previous report that WAGO-1 resides in P granules^46^. We next asked if WAGO-1 association with perinuclear granules was dependent on PGL-1 assembly. In animals expressing assembly-defective PGL-1(R123E)::SNAP, which fails to form granules efficiently (Fig. 4f), WAGO-1 was diffuse in about half the germlines (Fig. 6b), but granular in the other half (Fig. 6c). Therefore, WAGO-1 can assemble into P granules independently of PGL-1. To investigate if PGL-1 assembly is independent of WAGO-1, we generated an internal deletion that creates a frameshift and fails to express WAGO-1 protein (Fig. 6d and Supplementary Fig. 8c, see Methods). PGL-1::SNAP localized to perinuclear granules in the absence of WAGO-1 (Fig. 6d). The variable sizes of GLH-1, PGL-3, and WAGO-1 granules seen in PGL-1 dimerization-defective germlines may indicate that PGL-1 assembly mutations affect the efficiency of recruiting these proteins into P granules at the nuclear periphery. Regardless, we conclude from these studies that PGL-1 and WAGO-1 can assemble into granules independently of each other.

### WAGO-1 is a regulator of PGL-1-associated mRNA repression in P granules

**Fig. 7:**
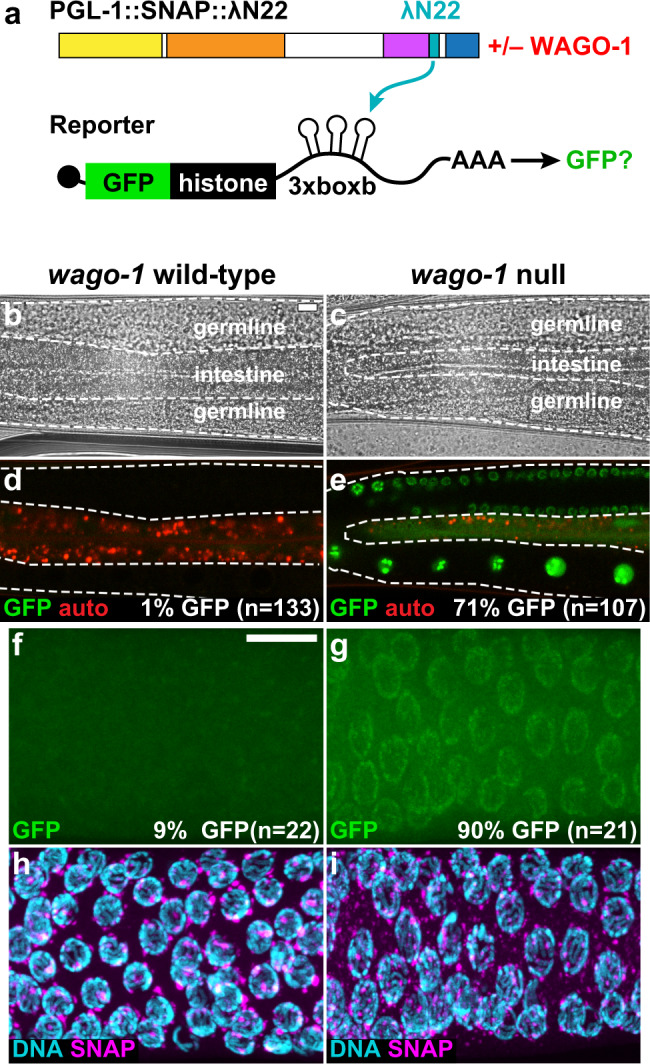
Argonaute WAGO-1 is required for PGL-1 tethered mRNA reporter repression. **a** Protein–mRNA tethering assay and WAGO-1. To test the necessity of WAGO-1 for mRNA repression, PGL-1::SNAP::λN22 germlines were analyzed for GFP reporter expression in the presence or absence of WAGO-1. N-terminal dimerization domain (NtDD, yellow), central dimerization domain (CDD, orange), SNAP (magenta), λN22 (light blue) and RGG repeats (blue), and GFP (green). Modified from^76^. **b**–**e** GFP reporter expression in germ cells of live animals with (**b**, **d**) wild-type *wago-1* or (**c**, **e**) *wago-1* null. (**b**, **c**) Brightfield image. (**d**, **e**) GFP fluorescence (green); auto fluorescence (red). *n*, number of animals scored for GFP expression. Scale bar, 10 μm, in (**b**) applies to (**b**–**e**) images. **f**–**i** Representative images of fixed germlines with (**f**, **h**) wild-type *wago-1* or (**g**, **i**) *wago-1* null. (**f**, **g**) GFP fluorescence (green). (**h**, **i**) DNA (DAPI, cyan) and PGL-1 granule formation seen by SNAP staining (magenta). *n*, number of germlines scored for GFP expression. Fisher’s exact test of *wago-1* wild-type vs. null (*p*-value < 0.0001). Scale bar, 10 μm, in (**f**) applies to images (**f**–**i**).

**Fig. 8:**
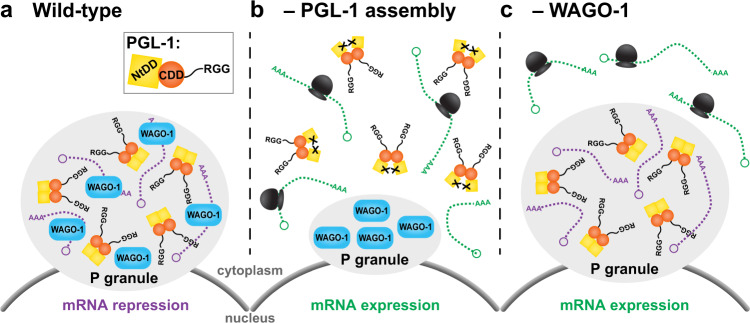
Model of P granule assembly and mRNA repression. **a** P granules assemble at the nuclear pore with assembly-competent PGL-1 protein. PGL-1 is shown as a dimer for simplicity but multivalent PGL-1s likely form an oligomeric protein–RNA network via its N-terminal dimerization domain (NtDD, yellow) and central dimerization domain (CDD, orange). WAGO-1 (blue) binds to RNA (purple), as expected for an Argonaute, and WAGO-1-associated RNAs are repressed, potentially by mRNA turnover (purple). **b** When PGL-1 NtDD cannot dimerize, PGL-1 fails to properly assemble into P granules at the nuclear periphery. WAGO-1 assembles independently into P granules. PGL-1-associated, non-granular mRNAs (green) are available for translation (ribosomes, black). **c** In the absence of cytoplasmic Argonaute WAGO-1, PGL-1 proteins assemble into P granules normally with its associated mRNA (purple), but loss of WAGO-1 perturbs repression of P granule-localized transcripts. PGL-1’s liquid droplet properties permit diffusion of some transcripts (green) into the cytoplasm for translation. See text for further discussion.

Finally, we explored the role of WAGO-1 in mRNA repression within granules (Fig. 7a). To this end, we tested PGL-1::SNAP::λN22 for its ability to repress reporter RNA in the presence or absence of WAGO-1. We again conducted our assays in living animals to ensure a large sample size (Fig. 7b–e) and fixed extruded gonads to visualize PGL in addition to GFP fluorescence (Fig. 7f–i). The reporter was repressed with wild-type WAGO-1 (Fig. 7d, f and Supplementary Fig. 9a–d), as expected, but de-repressed in the *wago-1* null mutant (Fig. 7e, g and Supplementary Fig. 9e–h), consistent with *wago-1* RNAi (Supplementary Fig. 8b). De-repression was seen in ~70% of germlines when assayed in living animals (Fig. 7e) and in ~90% of fixed gonads (Fig. 7g). In tethered PGL-1 germlines, GFP fluorescence levels were modestly increased with *wago-1* null versus wild-type (Supplementary Fig. 9i), so perhaps loss of *wago-1* causes partial de-repression. Loss of *wago-1* in non-tethered worms had no observable effect on fluorescence (Supplementary Fig. 10), arguing against *wago-1* alone affecting fluorescent reporter expression. Regardless, the percentages demonstrate strong but incomplete de-repression, a result consistent with WAGO-1 functioning redundantly with other Argonautes^46–48^. As expected, PGL-1::SNAP::λN22 assembles into granules, with or without WAGO-1 (Fig. 7h, i). The *gfp* reporter mRNA colocalizes modestly more with PGL-1 in animals with wild-type WAGO-1 (Supplementary Fig. 9a–d, j) than in *wago-1* null mutants (Supplementary Figs. 9e–h, j and 11). However, the total smFISH signal was higher in *wago-1* null mutants (Supplementary Fig. 9k), implying that PGL-1-mediated repression of mRNA transcripts by WAGO-1 is through mRNA turnover. We conclude that WAGO-1 is a regulator of PGL-1-associated mRNA repression in P granules. These results also provide direct evidence that granule formation alone is not sufficient for mRNA regulation (Fig. 8).

## Discussion

This work investigates the functional relationship between assembly of a paradigmatic liquid droplet, the *C. elegans* P granule, and the activities of its regulatory components. Our analyses make three key advances. First, we discover that PGL-1 dimerization of the N-terminal domain is required for its assembly into P granules. Second, we demonstrate that PGL-1-mediated mRNA repression relies on PGL-1 assembly. Third, we find that mRNA repression by PGL-1 employs the activity of at least one P granule constituent, the Argonaute WAGO-1. Together, these advances support a model that PGL-1 assembly is necessary for its biological function but not sufficient to repress the expression of localized mRNAs (Fig. 8). Below we discuss these advances and their implications for RNP granules more generally.

An emerging principle of RNP granule assembly is that multivalent macromolecular interactions drive granule formation^1,40^. This work extends that principle to nematode P granules and their assembly proteins, the PGLs (see Introduction). A previous study reported that PGL proteins possess a dimerization domain (DD) in their central region^32^, and here we report the discovery of a second PGL DD in the N-terminal region (NtDD). Thus, PGLs possess two protein folds that confer multivalency. Based on insights from the NtDD structure, we designed two distinct PGL-1 mutant proteins that are unable to dimerize in vitro and are severely compromised in vivo for assembly into P granules and fertility. These results provide evidence that PGL-1 dimerization is crucial for P granule assembly and for PGL-1’s biological function. Although attempts to disrupt dimerization via the central DD led to protein instability and hence could not test its biological significance^32^, we include both N-terminal and central DDs in our model for how PGLs mediate assembly into P granules (Fig. 8a). We speculate that PGL-3 alone can still assemble into granules, and that mutant PGL-1 can assemble with PGL-3 via the CDD. In doing so, mutant PGL-1 NtDD could prevent dimerization of both PGL-1 and PGL-3 NtDDs, and hence block proper granule formation. Another possibility is that a third PGL domain or peptide associates with P granules through direct or indirect interactions. A critical future direction is to investigate how each dimerization domain and other PGL regions contribute to higher order and likely oligomeric assembly.

Self-assembling, multivalent proteins have been identified for several RNP granules. Examples include TDP-43 in nuclear granules^49^, Oskar and Vasa for *Drosophila* polar granules^50–52^; EDC3 and LSM4 for P-bodies^53^; and MEG-3 and MEG-4 for embryonic P granules^9,38,54^. These various assembly proteins rely on a combination of multimerization domains and low complexity, intrinsically disordered sequences to facilitate granule formation^9,53–57^. A leading hypothesis of liquid droplet assembly invokes reliance on multiple, weak interactions among RNP constituents^1,40^. Our work highlights the idea that interactions between structured regions are a driving force in vivo for assembly of liquid droplet granules as well as their biological function (see below). Recombinant PGL proteins make liquid droplets on their own in vitro^9,11,12^, suggesting that PGLs possess regions responsible for low-affinity contacts in the full-length protein. We suggest that PGL uses both dimerization domains as well as additional low-affinity contacts to facilitate liquid droplet formation. Alternatively, the DDs may be subject to post-translational modifications that modulate their affinity in vivo. PGL did not require its C-terminal RGG repeats to form granules in mammalian culture^30^, but RGG repeats in other assembly proteins enhance granule formation^57^. The PGL RGG repeats may instead be needed to trigger robust granule assembly with RNA^12^, or impart liquid droplet properties associated with PGL in nematodes^10^. Regardless, the discovery that PGL proteins are multivalent with two dimerization domains advances our understanding how this paradigmatic liquid droplet assembles.

Our study employs a protein–RNA tethering assay to provide direct evidence that mRNA expression is repressed when mRNAs are recruited to adult germline P granules. Three lines of indirect evidence had previously led to this idea: P granules contain repressed mRNAs^19^; spermatogenic and somatic transcripts are aberrantly expressed in the absence of P granules^22–24^; and a variety of RNA regulatory factors colocalize in P granules, including inhibitory RNA-binding proteins, like the Pumilio homolog FBF-2^37^, and RNA regulatory enzymes, like the Argonaut/Piwi PRG-1^58^ and the deadenylase PARN-1^59^. Despite these clues, the significance of P granule assembly for biological function remained enigmatic. Our discovery of key amino acids required for PGL-1 assembly into granules allowed us to compare RNA regulation when coupled to assembly-competent or assembly-defective PGL-1. We found that a reporter mRNA tethered to assembly-competent PGL-1 protein localized with PGL-1 to P granules and was repressed, but a reporter mRNA tethered to an assembly-defective PGL-1 did not localize to granules and was expressed. Together, these results suggest that mRNA binding to granule-forming PGL-1 leads to repression (Fig. 8a, b).

We also tethered PGL-3, which, unlike PGL-1, had no detectable effect on reporter expression. Therefore, PGL-1 and PGL-3 may have distinct molecular roles, consistent with their distinct phenotypes and partners (see Introduction). We acknowledge that these results may not fully reflect the endogenous situation, because tethering to a reporter 3′UTR artificially localizes the mRNA to granules and the location of PGL-1 tethering within the reporter mRNA may affect its regulatory mechanism. Thus, additional experiments are needed to understand how PGL-1 regulates gene expression.

Expression of mRNAs localized within an RNP granule might be repressed by either of two broad mechanisms. Granule localization might recruit RNAs to form interactions with negative-acting factors (e.g., RNA turnover machinery) or it might sequester RNAs away from positive-acting factors (e.g., translational machinery). Our results suggest that localization with assembled PGL-1 and key repressors is the primary mechanism (Fig. 8). Previous work identified an in vitro RNase activity for PGL DD^32^. However, PGL-1 tethering of RNA, and subsequent recruitment into granules, is not sufficient for repression on its own, and also relies on a P granule-localized Argonaute, WAGO-1 (Fig. 8c). Previous studies reported that WAGO-1 is P granule-associated and represses transcripts that have been primed by the piRNA pathway as part of the secondary RNAi response^47^. Our tethering results broaden the role of WAGO-1 to repress mRNAs associated with the P granule scaffold. Previous studies also reported that WAGO-1 is functionally redundant with other Argonautes^46–48^. We suspect this redundancy may explain why some, not all, *wago-1* null mutant germlines repressed PGL-1 tethered reporter mRNAs. The specific mechanism for mRNA repression is still unclear, although results from this work favors RNA turnover, a mechanism that may be driven by PGL’s RNase activity or the Argonaute. Additional work must be done to understand the relative importance and individual roles of these Argonautes and PGL-1 in mRNA repression. PGL-1 tethering can be used in future studies to screen for additional factors involved in regulating mRNA expression.

Other cytoplasmic RNP granules have been proposed to repress mRNAs. Granule formation of a yeast amyloid-like RNA-binding protein correlates with translational inhibition of transcripts critical for gametogenesis^60^, and P-bodies contain mRNAs that are translationally repressed in cells^61^. Our work extends this idea further by showing the dependence of other enzymatic factors in liquid droplet granules for mRNA repression. Our model proposes that loss of PGL-1 dimerization compromises granule assembly and hence compromises the coalescence of RNAs with WAGO-1 and other P granule-associated repressors (Fig. 8b), while loss of WAGO-1 relieves granule-recruited mRNAs from repression (Fig. 8c). Therefore, the scaffolding function of PGL-1 is important but not sufficient for P granule function. We also propose that, without WAGO-1, mRNAs that are normally destined for P granule association can exist in either of two states. Those remaining in the granule are not expressed due either to the presence of other RNA repressors or the absence of ribosomes, but those diffusing out of the liquid droplet granule reach the translational machinery for expression (Fig. 8c). By extension, we propose that other RNP granules with liquid droplet properties may require both scaffolding proteins and active enzymatic factors to regulate their mRNAs.

This work adds to the emerging theme that granules play a general role in regulating the function of their components. For example, stress granules sequester the mTORC1 protein complex to block activation of mTOR signaling^62^, mammalian cells can trap hormones and melanin in amyloid-like aggregates to prevent active signaling^63,64^, and liquid droplet granule formation of cGAS with cytosolic DNA triggers its enzymatic activity in vitro and cell culture^65^. The functional relationship between PGL-1 and WAGO-1 is unknown. PGL-1 may simply recruit or retain mRNAs in P granules to be regulated by WAGO-1. A more enticing model is that PGL-1 forms a higher-ordered, RNP complex with mRNA that enhances WAGO-1’s biochemical activity. Further studies that pair insights into mechanisms of granule assembly with direct in vivo assays of regulation will be pivotal moving forward to decipher the mechanistic function of other RNP granules in their biological context.

## Methods

### Protein expression and purification

We previously used *C. elegans* PGL-3 recombinant protein and limited proteolysis to identify a central dimerization domain (CDD)^32^. While we could express CDD efficiently we could not express recombinant protein that was N-terminal to the cleavage site (PGL-3 amino acid residues 205-206). We tried moving the six-histidine purification tag to the N- and C- termini, shortened the protein regions used for expression, and tried several different orthologs with little success. The insight came after aligning protein sequences of several *Caenorhabditis sp*. and studying the CDD domain boundary (Supplementary Fig. 1c). Protease cleavage occurred in a conserved portion of the N-terminal region and this region was disordered in our DD crystal structures. After inclusion of this region (PGL-3 amino acid residues 205-212), we could express and purify recombinant N-terminal protein from *C. elegans* PGL-1 and its orthologs. We refer to this region as the N-terminal domain (NtDD).

This study used *C. elegans* PGL-1, PGL-3, and *C. japonica* PGL-1 recombinant NtDD proteins. The *C. elegans* PGL-3 coding region was PCR amplified from cDNA (Supplementary Table 2). A his-tagged, codon-optimized (*Escherichia coli*) version of *C. japonica* and *C. elegans* PGL-1 NtDD was ordered as DNA fragments (IDT, Coralville, IA and Twist Bioscience, San Francisco, CA). Constructs were cloned into a pET21a vector (MilliporeSigma, Burlington, MA) using Gibson Assembly cloning^66^, and plasmids transformed into Rosetta2 cells (MilliporeSigma, Burlington, MA) or BL21 cells (Invitrogen, Carlsbad, CA). Mutations were introduced with primers (Supplementary Table 2) and cloned into pET21a vectors via Gibson Assembly. Cultures were grown at 37 °C with shaking (225 rpm) until ~0.8 OD, cooled for 30–60 min, and induced with a final concentration of 0.1 mM IPTG. Cultures were then grown at 16 °C with shaking (160 rpm) for 16–18 h, collected, and bacterial pellets frozen until use. Selenomethionine-incorporated *C. japonica* protein was expressed in SelenoMethionine Medium Complete (Molecular Dimensions, Suffolk, UK), and grown, induced, and collected in a similar manner.

Bacterial pellets were defrosted on ice and reconstituted in lysis buffer (20 mM sodium phosphate pH 7.4, 300 mM NaCl, 10 mM imidazole, 5 mM beta-mercaptoethanol (BME)) with protease inhibitors (cOmplete™ EDTA-free, Roche, Indianapolis, IN). Lysozyme (Sigma-Aldrich, St. Louis, MO) was added at 50 µg/ml and incubated on ice for 20 min prior to lysis in a French press or microfluidizer. Samples were spun at low (3220*g*, 4 °C, 20 min) and high speed (10,000*g*, 20 °C, 10 min), then incubated with 1.5 ml NiNTA beads (Thermo Fisher Scientific, Waltham, MA) for 1 h at 4 °C with rotation. Sample supernatant was separated by gravity flow, washed twice with lysis buffer, and eluted using lysis buffer with increasing imidazole concentrations (20, 40, 60, 80, 100, 250 mM). Eluted samples were monitored via Bradford assay (Bio-Rad, Hercules, CA), and dialyzed overnight in HN buffer (20 mM HEPES pH 7.4, 100 mM NaCl). The dialyzed samples were concentrated with a Centriprep 10K concentrator (MilliporeSigma, Burlington, MA) or Vivaspin 10K concentrator (Sartorius, Göttingen, Germany). For crystallography, calcium was added to 1 mM CaCl_2_, and the histidine tag removed with carboxypeptidase A^67^ bound to agarose (Sigma, St. Louis, MO) at a ratio of 10 protein:1 enzyme (w/w). These protein–enzyme samples were incubated at room temperature (~20 °C) for 45–90 min with rotation prior to supernatant elution by centrifugation in microflow columns (Thermo Fisher Scientific, Waltham, MA). All samples were run on a sizing column, either the S200 (GE Healthcare, Chicago, IL) or an Enrich SEC 650 (Bio Rad, Hercules, CA) in HNT buffer (20 mM HEPES pH 7.4, 100 mM NaCl, 0.5 mM TCEP pH 7.4). Fractions containing recombinant protein were collected, concentrated in a Vivaspin 10K concentrator or an Amicon 10K concentrator (MilliporeSigma, Burlington, MA), and protein concentration estimated by A280. Samples were frozen in liquid nitrogen or used immediately. SDS-PAGE gels of the recombinant proteins are available in the Source Data File.

### Crystallization and structure determination

*C. elegans* PGL-1, *C. elegans* PGL-3, and *C. japonica* PGL-1 NtDD recombinant protein were screened in crystallization conditions using 400 nl hanging and sitting drop 96-well trays set up with the Mosquito (TTP Labtech, Cambridge, MA) in 20 °C. Several conditions produced labile crystal plates. Data were collected to 4 Å from *C. elegans* PGL-1 crystal plates, determined to have a very large unit cell (86 Å × 86 Å × 460 Å) and P6 point group, and eventually determined to have perfect merohedral twinning. *C. japonica* PGL-1 also crystallized as large (60–150 Å) rhomboid crystals in 40–45% PEG 400 at low (Na Citrate pH 5.5–6.0) and physiologic pH (imidazole pH 7.5–8.0). Crystals grown in citrate or imidazole both diffracted well, but we used imidazole (100 mM imidazole pH 7.5, 45% PEG 400, 1 mM TCEP pH 7.4) due to its higher reproducibility for large crystals and its modestly better resolution. The crystals did not require additional cryo-protection due to the high PEG 400. We eventually collected a full data set to 1.5 Å in space group C2. Data were collected at LS-CAT Sector 21 (21-ID-D, 21-ID-G).

PGL-1 NtDD forms a novel domain. Novelty and translational pseudosymmetry precluded us from using any model for molecular replacement. Trial heavy atom soaks also proved unfruitful, and the *C. japonica* PGL-1 NtDD has just two methonines past the start codon, making selenomethionine phasing challenging. To boost anomalous signal, we mutated two non-conserved isoleucines to methionines (I63M, I212M). This methionine mutant provided phases to 3.6 Å by single anomalous dispersion (SAD) that we used to build a 1.6 Å model of the mutant protein (PDB 5W4D [10.2210/pdb5W4D/pdb]) Ramachandran statistics were 98.68% favored, 1.32% allowed, and 0.00% outliners). We used this model for molecular replacement into the wild-type data set to build a complete 1.5 Å model (PDB 5W4A [10.2210/pdb5W4A/pdb] Ramachandran statistics were 98.1% favored, 1.90% allowed, and 0.00% outliners). Amino acid G114 and the first three residues of the N-terminus could not be visualized in all of the copies of the ASU. The RMSD was calculated with only the C-alpha carbons present in all four ASU copies. No symmetry constraints were used in refinement. Data and model statistics are in Supplementary Table 2.

### Size exclusion chromatography with multi-angle laser light scattering (SEC-MALS)

Molecular weights of *C. elegans* PGL-1 NtDD wild-type and mutant recombinant protein were determined by conducting SEC-MALS experiments using an AKTA FPLC (GE Healthcare Biosciences) with a SEC 650 sizing column, a refractive index detector (Optilab T-rEX; Wyatt Technology), and a multiple light scattering detector (Dawn Heleos II; Wyatt Technology). Wild-type and mutant PGL-1 NtDD samples were injected at 1 mg/ml in HNT buffer. Flow rate was set at 0.4 ml/min, and data were collected at 2 s intervals. Data processing and analysis were performed using the ASTRA software (Wyatt Technology).

Molecular weights of *C. elegans* PGL-3 NtDD wild-type and mutant recombinant protein were determined by conducting SEC-MALS experiments using Agilent Technologies 1260 LC HPLS system (Agilent Technologies, Santa Clara, CA) equipped with Dawn^®^ Heleos^™^II 18-angle MALS light scattering detector, Optilab^®^ T-rEX^™^ (refractometer with EXtended range) refractive index detector, WyattQELS^™^ quasi-elastic (dynamic) light scattering (QELS) detector and ASTRA software (all four from Wyatt Technology Europe GmbH, Dernbach, Germany). A total of 500 µl (1 mg/ml) of the samples in HNT buffer (20 mM HEPES pH 7.5, 100 mM NaCl, 0.5 mM TCEP pH 7.4) were injected and run on a Superdex 75 10/300 GL column (GE Healthcare) pre-equilibrated with the same buffer, at a flow rate of 0.5 ml/min at 20 °C. Lysozyme (Sigma-Aldrich, St. Louis, MO) was used as a control.

### Mammalian cell culture maintenance, transfection and imaging

Full-length PGL-1 was cloned into a pcDNA 3.1 vector (Thermo Fisher Scientific, Waltham, MA) with a C-terminal eGFP and OLLAS epitope linker (Supplementary Table 2). Mutations to PGL-1 were created using Gibson Assembly cloning^66^ (Supplementary Table 2). Chinese Hamster Ovary (CHO) cells (ATCC, Manassas, VA) were propagated according to distributor’s recommendations. Briefly, cells were grown in F-12K Medium (Thermo Fisher Scientific, Waltham, MA) with 10% fetal bovine serum (Gibco), and split with Trypsin 0.25% (Gibco) every 2–3 days. Cells were grown to 70% confluence and transfected with TransIT-CHO Transfection Kit (Mirus Bio LLC, Madison, WI). Transfected cells were split the following day and grown in ibiTreat 15 µ-Slide 8 well slides (Ibidi, Madison, WI) overnight. Hoechst stain (Invitrogen, Carlsbad, CA) was added to wells prior to imaging by confocal microscopy for GFP and Hoechst fluorescence, and transmitted light. Well dilutions were chosen based on adequate cell spacing to discern each cell, and 25 fields of view were taken based on the highest concentration of GFP-positive cells. Experiments were repeated four times with similar results. During image collection, we observed a single example of a granule-like blob in the PGL-1(K126E K129E)::GFP. The cell appeared unhealthy, and thus the granule may be an artifact of cell death, but we included it in our study for completeness. Fisher’s exact test was performed to compare imaging results.

### Worm maintenance, CRISPR mutagenesis, fertility, and imaging

Frozen strains:

N2 Bristol

JK5687: *pgl-1(q894*)[PGL-1::SNAP] *IV*

JK5902: *pgl-1(q975*)[PGL-1(R123E)::SNAP] *IV*

JK6158: *wago-1(q1087)*[WAGO-1::3xV5]; *pgl-1(q894*)[PGL-1::SNAP] *IV*

JK6159: *wago-1(q1089)*[WAGO-1(null deletion)::3xV5]; *pgl-1(q894*)[PGL-1::SNAP] *IV*

JK6157: *wago-1(q1087)*[WAGO-1::3xV5]; *pgl-1(q975*)[PGL-1(R123E)::SNAP] *IV*

JK5898: *glh-1(q858*)[GLH-1::3xMYC] *I; pgl-1(q894*)[PGL-1::SNAP] *IV; pgl-3(q861*)[PGL-3::3xV5] *V*

JK5970: *qSi375*[(mex-5 promoter::eGFP::linker::his-58::3xboxb::tbb-2 3′UTR) *weSi2] *II; pgl-1(q894*)[PGL-1::SNAP] *IV*

JK5873: *qSi375*[(mex-5 promoter::eGFP::linker::his-58::3xboxb::tbb-2 3′UTR) *weSi2] *II; pgl-1(q994*)[PGL-1::SNAP::λN22]*/nT1[qIs51](IV;V)*

JK5874: *qSi375*[(mex-5 promoter::eGFP::linker::his-58::3xboxb::tbb-2 3′UTR) *weSi2] *II; pgl-1(q994*)[PGL-1::SNAP::λN22]*/nT1[qIs51](IV;V)*

JK6149: *qSi375*[(mex-5 promoter::eGFP::linker::his-58::3xboxb::tbb-2 3′UTR) *weSi2] *II; pgl-1(q994*)[PGL-1::SNAP::λN22]*/nT1[qIs51](IV;V)*

JK6150: *qSi375*[(mex-5 promoter::eGFP::linker::his-58::3xboxb::tbb-2 3′UTR) *weSi2] *II; pgl-1(q994*)[PGL-1:SNAP:λN22]*/nT1[qIs51](IV;V)*

JK6147: *wago-1(q1089)*[WAGO-1(null deletion)::3xV5]; *qSi375*[(mex-5 promoter::eGFP::linker::his-58::3xboxb::tbb-2 3′UTR) *weSi2] *II; pgl-1(q994*)[PGL-1::SNAP::λN22]*/nT1[qIs51](IV;V)*

JK6148: *wago-1(q1089)*[WAGO-1(null deletion)::3xV5]; *qSi375*[(mex-5 promoter::eGFP::linker::his-58::3xboxb::tbb-2 3′UTR) *weSi2] *II; pgl-1(q994*)[PGL-1::SNAP::λN22]*/nT1[qIs51](IV;V)*

JK6367: *qSi375*[(mex-5 promoter::eGFP::linker::his-58::3xboxb::tbb-2 3′UTR) *weSi2] *II*

JK6368: *wago-1(q1089)*[WAGO-1(null deletion)::3xV5]; *qSi375*[(mex-5 promoter::eGFP::linker::his-58::3xboxb::tbb-2 3′UTR) *weSi2] *II*

CDE15: *qSi375*[(mex-5 promoter::eGFP::linker::his-58::3xboxb::tbb-2 3′UTR) *weSi2] *II; pgl-3(ddc1)*[PGL-3::3xFLAG] *V*

CDE16: *qSi375*[(mex-5 promoter::eGFP::linker::his-58::3xboxb::tbb-2 3′UTR) *weSi2] II; pgl-3(ddc3)[PGL-3::λN22::3xFLAG] *V*

Worm strains that could not be frozen:

1. *pgl-1(q960*)[PGL-1(K126E K129E)::SNAP] *IV*

2. *glh-1(q858*)[GLH-1::3xMYC] I; pgl-1(q960)[PGL-1(K126E K129E)::SNAP] *IV; pgl-3(q861*)[PGL-3::3xV5] *V*

3. *qSi375*[(mex-5 promoter::eGFP::linker::his-58::3xboxb::tbb-2 3′UTR) *weSi2] II; pgl-1(q1053)[PGL-1(K126E K129E)::SNAP::λN22]*/nT1[qIs51](IV;V)*

*C. elegans* were maintained on nematode growth medium (NGM; 25 mM KPO_4_ pH 6.0, 5 mM NaCl, 1 mM CaCl_2_, 1 mM MgSO_4_, 2.5 mg/ml tryptone, 5 µg/ml cholesterol, 1.7% agar) petri dishes and fed *E. coli* OP50, as previously reported^68^. For CRISPR-Cas9 mutagenesis, a Cas9 protein co-conversion approach was used^69^ (Supplementary Tables 3 and 4). Briefly, worms were injected with a target CRISPR-Cas9 RNA (crRNA) or a plasmid expressing a Cas9-scaffold with tandem target sequence RNA (sgRNA) to a gene of interest^69^, a target crRNA to *dpy-10* or *unc-58*, a scaffolding tracrRNA (IDT), recombinant Cas9 protein^70^, a *dpy-10/unc-58* repair DNA oligo that inserted a dominant mutation^69^, and an epitope tag/missense mutant repair oligo or PCR product (Supplementary Table 4). See Supplementary Tables 3 and 4 for guide RNAs and repair templates used. F1s with the co-injection marker phenotype were additionally screened by a combination of PCR without or with restriction enzyme digest (Supplementary Table 2) to identify those with the repair of interest (Supplementary Table 2). In JK5687, a SNAP tag^35^ was inserted between PGL-1 amino acids G713 and G714 in N2 worms. A 3xMYC tag was added to the N-terminus of GLH-1 between G17 and F18. A 3xV5 tag, 3xFLAG, and 3xFLAG::λN22 were added in the C-terminal region of PGL-3 between residues G627 and S628. A 3xV5 tag was added to the C-terminus of WAGO-1 between residues E914 and A915. To generate the *wago-1* null allele, a WAGO-1::3xV5 allele was mutated so that 648 base pairs were deleted from the N-terminus and proper coding frame shifted to add premature stop codons (Supplementary Fig. 8c). The *wago-1* null allele was confirmed by staining and imaging (Fig. 6d). F2s were PCR screened to identify homozygous SNAP alleles and the PCR product sequenced to confirm proper repair (Supplementary Table 2). Three worm strains were too infertile to freeze. All worms were outcrossed at least twice with N2, with the exception of *glh-1(q858*)[GLH-1::3xMYC] *I; pgl-1(q960*)[PGL-1(K126E K129E)::SNAP] *IV; pgl-3(q861*)[PGL-3::3xV5] *V* that was backcrossed with JK5898.

Worms were singled into the peripheral wells of a 24-well plate that contained NGM agar and OP50 bacteria. Worms were allowed to propagate for 5 days at 20 °C or 25 °C, and then scored for progeny and gravid progeny. We report the progeny numbers here. A Fisher’s exact test was performed to compare fertility results.

### Immunoblotting

To check PGL-1::SNAP protein expression, worms were boiled in 5x sample buffer (250 mM Tris pH 6.8, 25 mM EDTA pH 8.0, 25% glycerol, 5% SDS, 500 mM beta-mercaptoethanol), run on an SDS-PAGE gel, and transferred to PVDF. The blot was blocked in 5% dehydrated milk in PBS-T (PBS + 0.1% Tween-20), incubated overnight with anti-SNAP antibody (1:1000) (NEB, Ipswich, MA), washed and probed with goat-anti-rabbit horseradish peroxidase secondary antibody (1:10000) (Invitrogen, Carlsbad, CA), and developed with ECL substrate (Thermo Fisher Scientific, Waltham, MA) and film (Kodak, Rochester, NY). Radiographs were scanned, contrast adjusted and cropped (Photoshop, Adobe Creative Cloud) as shown. Full immunoblots are available in the Source Data File.

### Fluorescent imaging

To analyze GFP reporter expression, L4 larvae were propagated for approximately 24 h to adulthood at 20 °C, placed in M9 with 0.1 mM levamisole on a glass slide with a cover slip, imaged at 10x magnification on a compound microscope and counted for the presence or absence of GFP fluorescence in their germlines. Numbers represent totals from two separate experiments. A Fisher’s exact test was performed to compare examined reporter strains. The reporter images of live worms were taken of worms treated in a similar manner and visualized on a Leica SP8 scanning laser confocal microscope.

For confocal imaging, germlines were extruded, fixed with 1–3% paraformaldehyde (Electron Microscopy Sciences, Hatfield, PA) and permeabilized with 0.5% Triton-X in PBS-T (PBS + 0.1% Tween-20)^71^. Germlines were incubated with 1 μg/ml primary antibodies overnight [anti-MYC (JAC6 (rat) (1:1000), Bio-Rad, Hercules, CA); anti-V5 (sv5-Pk1 (mouse) (1:1000), Bio-Rad, Hercules, CA); anti-FLAG (M2 (mouse) (1:1000), Millipore-Sigma; Burlington, MA)] or 30 nM SNAP JF 549 ligand^72^ for 1 h, stained with fluorophore-labeled secondary antibodies (1:1000) (Alexa 488 Donkey anti-Mouse, Alexa 555 Donkey anti-Mouse, Alexa 647 Donkey anti-Mouse, Alexa 488 Goat anti-Rabbit; Invitrogen, Carlsbad, CA) and DAPI 0.5 µg/ml (Invitrogen, Carlsbad, CA), washed and mounted in Vectashield (Vector Laboratories, Burlingame, CA). Quantitation of GFP and SNAP fluorescence was performed in ImageJ by quantitating the summed intensity projections of confocal stacks, similar to^73^. Briefly, a line (40 pixel width) was drawn along the germline axis. Pixel intensity was measured using plot profile, and intensities averaged and plotted in Excel. Multiple, individual *t*-tests between strains were performed at each distance point using GraphPad Prism 8 version 8.4.3 (471) for MacOS (GraphPad Software). Each row was analyzed individually, without assuming a consistent standard deviation.

### Single molecule fluorescence in situ hybridization (smFISH)

For smFISH, gonads were extruded, fixed, and hybridized with single molecule FISH probes^36^. Briefly, the *gfp* exon probe set contains 38 unique oligonucleotides labeled with CAL Fluor Red 610. Probes were dissolved in RNase-free TE buffer (10 mM Tris-HCl, 1 mM EDTA, pH 8.0) to create a 250 µM probe stock. Mid-L4 stage animals were grown on OP50 for 24 h, then dissected in PBS + 0.1% Tween-20 + 0.25 mM levamisole. Animals were fixed in 4% paraformaldehyde for 20 min, incubated at room temperature in PBS-T (PBS + 0.1% Tween-20) for 10–25 min, and equilibrated in smFISH wash buffer (30 mM sodium citrate pH 7.0, 300 mM NaCl, 1% formamide, 0.1% Tween-20, DEPC water) for 10–16 min. Samples were then incubated in hybridization buffer (30 mM sodium citrate pH 7.0, 300 mM NaCl, 1% formamide, 10% dextran sulfate w/v, DEPC water) plus 0.5 µM smFISH probe at 37 °C for 26–44 h. 30 nM SNAP 549 ligand was added during the smFISH wash buffer + DAPI wash; samples were washed at 37 °C for approximately 60 min. Finally, samples were resuspended in 12 µl ProLong Gold antifade mounting medium (Thermo Fisher Scientific, Waltham, MA), mounted on glass slides, and cured in a dark drawer for at least 24 h before imaging.

Confocal imaging for smFISH and protein fluorescence. Most samples were imaged using a Leica SP8 scanning laser confocal microscope, taking 0.3 µm (smFISH experiments) or 1 µm (other non-smFISH confocal imaging experiments) slices in sequence. Maximum intensity partial stack projections were generated and brightness adjusted using ImageJ^74^. PGL-3 tethering experiments were imaged using a 3i-Zeiss spinning disk confocal microscope that consisted of a 3i spinning disk system [CSU-X1 M1 Spinning disk confocal, a Prime BSI CMOS camera and 405, 488, and 561 nm lasers (Intelligent Imaging Innovations Inc., Denver, CO)] mounted on an AxioObserver (ZEISS, Oberkochen, Germany). Z-stacks were taken at 63x, 0.27 µm slices, and maximum intensity stack projections were generated and brightness adjusted using ImageJ as performed in other samples. Approximately 5 μm thickness maximum intensity images are presented in smFISH figures. All images were treated equally in ImageJ and Photoshop, with the exception of the transmitted light images. Imaging experiments were repeated at least twice with similar results, with the exception of PGL-1(K126E K129E)::SNAP::λN22 worms.

For GFP levels, three equivalent sized boxes (technical replicates) were made over random nuclei in a maximum projection germline image and quantified for GFP fluorescence. These technical replicates were averaged and calculated for each worm germline imaged (biological replicate). Graphs were made in Prism, with each dot corresponding to a single biological sample. Ordinary, one-way ANOVA was performed to compare GFP levels in PGL-1 (Supplementary Fig. 2n) and PGL-3 (Supplementary Fig. 3b) tethering experiments. Student *t*-tests were performed in the PGL-1 tethering experiments (Supplementary Figs. 2o, p and 9i). Gonads were treated as biological replicates.

### smFISH image analysis

To quantify *gfp* smFISH signal localization between samples, *gfp* smFISH signal and PGL-1::SNAP signal were each detected then colocalized using Imaris version 9.3.1. The same batch settings were run on all directly compared images. In the 3D view for each image, detection for both *gfp* and PGL-1::SNAP surfaces was checked. If necessary, the edit tool was used to select and delete *gfp* and/or PGL-1::SNAP surfaces that were detected outside the imaged germline (e.g., on nearby intestine tissue). The *gfp* surface object was selected and the total *gfp* signal was pulled from the statistics tab (Detailed tab, select Average Values from dropdown, “Intensity Sum Ch = 4 Img = 1” row and “Sum” column) and stored in an excel file. The Surface-Surface coloc was run from Surface-Surface coloc XTension. The *gfp* and PGL-1::SNAP channels were selected and the “no smoothing” option was selected. The same statistics tab navigation was used to pull GFP intensity from the ColocSurface objects that were created by the XTension. Total GFP intensity in the colocalized surfaces was divided by total GFP intensity in the germline to calculate how much GFP signal is located in the PGL granules. Some *gfp* and PGL-1::SNAP colocalization values were calculated as over 100%. Although colocalization was performed using the Imaris user interface, the Surface-Surface Colocalization XTension ran an available MATLAB algorithm. We observed that the Surface-Surface colocalization created a new set of colocalization surfaces, which are differently shaped and larger than the surfaces written with Imaris algorithms to detect *gfp* or PGL-1::SNAP surfaces (Supplementary Fig. 10l). Therefore, the total *gfp* intensity in the coloc surfaces was sometimes larger than in the *gfp* surfaces.

PGL granule staining is sensitive to physical perturbation and some germlines lacked PGL-1::SNAP signal entirely or almost entirely. GFP smFISH and PGL-1::SNAP signal overlap was therefore calculated from the top 66% of images in terms of PGL-1::SNAP Intensity (Detailed tab, select Average Values from dropdown, “Intensity Sum Ch = 5 Img = 1” row and “Sum” column). Excluding the low-PGL images increased median signal overlap from 26% to 34% in PGL-1::SNAP and from 41% to 57% in PGL-1::SNAP::λN22 (Supplementary Fig. 2o). Excluding the low-PGL images increased median signal overlap from 113% to 121% in PGL-1::SNAP::λN22 with WAGO-1 and from 74% to 81% in PGL-1::SNAP::λN22 without WAGO-1 (Supplementary Fig. 9j). See Supplementary Table 5 for the Imaris settings used.

Each of the two independent samples were run through a two-tailed *t*-test to calculate significance. We used an online calculator (http://vassarstats.net). Data points for quantified fluorescence and smFISH are available in the Source Data File.

Data were analyzed using a Dell Precision 5820 with a 64-bit Windows 10 Education operating system, an Intel(R) Xeon(R) W-1245 CPU 3.70 GHz processor, and 128 GB of RAM. Imarisx64 9.3.1 and ImarisFileConverterx64 9.3.1 were installed along with MATLAB R2018b. The Surface-Surface Colocalization XTension was downloaded and installed from the Bitplane XTension File Exchange at http://open.bitplane.com/tabid/235/Default.aspx?id=111.

### Reporting summary

Further information on experimental design is available in the Nature Research Reporting Summary linked to this paper.

## Supplementary information

Supplementary Information

Peer Review File

Reporting Summary

## Data Availability

The data that support this study are available from the corresponding author upon reasonable request. Model coordinates and data are available at RCSB (www.rcsb.org) with accession codes 5W4A and 5W4D. Source data are provided with this paper.

## Source data

Source Data
