# Supplementary material for: C. elegans germ granules require both assembly and localized regulators for mRNA repression
Source: Nat Commun. 2021 Feb 12;12:996. doi: 10.1038/s41467-021-21278-1 (PMC7881195; doi:10.1038/s41467-021-21278-1)

SUPPLEMENTARY INFORMATION

Supplementary Tables and Figures

Supplementary Table 1. Data collection and refinement statistics

Supplementary Table 2. DNA primers

Supplementary Table 3. CRISPR-Cas9 guide RNAs

Supplementary Table 4. CRISPR-Cas9 repair DNA oligos and PCR product

Supplementary Table 5. Imaris batch detection settings

Supplementary Figure 1. PGL sequence alignment and the *pgl-1* locus
(a-b) *pgl-1* and *pgl-3* primary transcripts. 5' and 3' UTRs are grey, exons are white, numbered 1-8 and separated by introns. Allleles and sites of *pgl-1* and *pgl-3* mutations are labeled, including location of SNAP tag (magenta) and λN22 fusion (blue). (c) Sequence alignment of PGL N-terminal Dimerization Domain (NtDD) in *C. elegans* (*Ce*), *C. japonica* (*Cj*), *C. brenneri* (*Cbn*), *C. briggsae* (*Cbr*), *C. remanei* (*Cr*). Alignment and conservation (cons.) determined by T-Coffee [1]. Starred residues (*) are identical. Period (.) and colon (:) residues are similar. Residues participating in salt bridges only are in orange. Residues participating in hydrogen bonds only are in yellow. Residues forming both hydrogen bonds and salt bridges are highlighted in red. *C. elegans* PGL-1 missense mutations and their allele numbers are labeled. Dashed lines mark the newly-annotated end of PGL-1 NtDD domain and start of PGL-1 Central Dimerization Domain (CDD).

Supplementary Figure 2. GFP reporter transcript tethering to P granules
(a) Single molecule fluorescent in situ hybridization (smFISH) in nematode germ cells to visualize RNA expression and localization. Nucleus in light blue, P granules in magenta, and GFP coding region in green. (b-m) Gonads were extruded from animals harboring the *gfp* reporter and (b-e) PGL-1::SNAP (n=30); (f-i) PGL-1::SNAP::λN22 (n=27); (j-m) PGL-1 (K126E K129E)::SNAP::λN22 (n=10). Gonads were fixed and imaged for *gfp* RNA using (b,f,j) smFISH (yellow); (c,g,k) GFP protein fluorescence (green); (d,h,l) DNA (DAPI, cyan) and SNAP (magenta). The smFISH, DNA and SNAP images are merged in e,i,m. White arrows mark examples of intranuclear puncta; black arrows mark examples of cytoplasmic puncta. Scale bar, 5 μm, for all images, except for 2.5-fold enlarged images in inset. For germline location, see Supplementary Fig. 6b. n= biologically independent animals examined over 2 independent experiments. (N) Quantification of GFP signal in confocal images for the GFP boxB reporter expressed with PGL-1::SNAP (n=23), PGL-1::SNAP::λN22 (n=24), PGL-1(K126E K129E)::SNAP::λN22 (n=10). A silenced GFP reporter (n=25) served as a negative control. Mean GFP fluorescent signal (line) and standard deviation reported in arbitrary units. An ordinary one-way ANOVA statistical test was used to compare data. ***, p-value <0.0001; n.s.1, p-value = 0.5171; n.s.2, p-value = 0.8790. n= biologically independent animals (3 technical replicates) examined over 2 independent experiments. (o) smFISH and PGL-1::SNAP signal colocalization in germlines expressing the GFP reporter and either PGL-1::SNAP or PGL-1::SNAP::λN22. Reported as the Sum of smFISH GFP Intensity in SNAP:smFISH colocalized binned signal)/(Total smFISH GFP binned signal). Co-localization greater in germlines expressing PGL-1::SNAP::λN22 (**, p-value = 0.0003). (p) Total smFISH signal in germlines expressing the GFP reporter and either PGL-1::SNAP or PGL-1::SNAP::λN22. Total smFISH signal is less in germlines expressing PGL-1::SNAP::λN22 (**, p-value = 0.002). Box plots represent the first and third quartiles, black

line is median, whiskers to min and max values. n= biologically independent animals examined over 2 independent experiments. Two-tailed t tests were used to calculate significance between smFISH data. See Methods for more details.

Supplementary Figure 3. PGL-3 tethering does not affect GFP boxB reporter expression
(a) Example fixed, extruded germline images of GFP expression in N2 wild type (n= 21) or worms expressing the GFP boxB reporter with PGL-3::3xFLAG (n= 21) or PGL-3::λN22::3xFLAG (n= 21). DNA (DAPI, cyan), GFP (green), and PGL-3 (FLAG, magenta). Scale bar (white), 5 µm. The experiment was repeated 3 times with similar results. (b) GFP fluorescence quantitation of N2 wild type (n= 21) or worms expressing the GFP boxB reporter with PGL-3::3xFLAG (n= 21) or PGL-3::λN22::3xFLAG (n= 21). n= number of biologically independent animals (3 technical replicates) examined over 3 independent experiments. An ordinary one-way ANOVA statistical test was used to compare data. GFP expression in PGL-3::3xFLAG versus PGL-3::λN22::3xFLAG worms not significant (n.s., p-value = 0.4302). ***, p-value <0.0001.

Supplementary Figure 4. GFP reporter transcripts localize to P granules when tethered
(a-f) Six additional examples of germlines harboring PGL-1::SNAP::λN22 (n=27). Gonads were fixed and imaged for *gfp* RNA using smFISH (yellow), DNA (DAPI, cyan) and SNAP (magenta). The three are merged in images on right. White arrows mark examples of intranuclear puncta; black arrows mark examples of cytoplasmic puncta. Scale bar, 5 µm, for all images, except for 2.5-fold enlarged images in inset. For germline location, see Supplementary Fig. 6b. n= biologically independent animals examined over two independent experiments.

Supplementary Figure 5. Supplemental biochemical and structural analyses of PGL-1 and PGL-3 NtDD.
(a-c) Coomassie-stained polyacrylamide gel of recombinant PGL-1 and PGL-3 NtDD wild-type and mutant protein. Ladder marker sizes labeled in kilodaltons (kDa) on right. SDS-PAGE gels were repeated twice with similar results. (a) Recombinant *C. japonica* (Cj) PGL-1 N-terminal Dimerization Domain (NtDD) protein used for crystallization. Recombinant *C. elegans* PGL-3 NtDD protein included for comparison. (b) Wild-type and mutant *C. elegans* PGL-1 NtDD recombinant proteins used for biochemical characterization. (c) Wild-type and mutant *C. elegans* PGL-3 NtDD recombinant proteins used for biochemical characterization. (d) Tables of predicted hydrogen bonds and salt bridges at the NtDD dimerization interface. Amino acid numbers correspond to PGL-1 NtDD. *C. japonica* and corresponding *C. elegans (parentheses)* residues listed. NtDD mutations tested in bold. (e,f) Surface representation of the NtDD dimerization interface. (e) Amino acids colored by identity (red) and similarity (pink). (f) Amino acids at dimerization interface (purple). (g) SEC-MALS of bovine serum albumin (BSA). Differential refractive index (left y axis) in arbitrary units (blue). Molecular weight (MW, right y axis) in daltons (Da, red; 64360 Da +/- 3.478%). (h,i) Size exclusion chromatography and multi-angle light scattering (SEC-MALS) of recombinant PGL-3 (h,i) NtDD wild type, (h) K126E K129E and (i) R123E proteins. A280 UV absorbance (left y axis) was normalized to the maximum value. Molecular weight (MW, right y axis) for MALS in daltons (Da). Wild-type protein (blue) measured the approximate size of a dimer, while both mutant proteins (red) measured approximately as monomers.

Supplementary Figure 6. Supplemental images of PGL-1 dimerization mutants
(a) Statistics of strain fertility results reported in Fig. 4b. A two-sided Fisher's exact test was used to compare data. (b) Schematic of an adult hermaphrodite germline. An asterisk marks proliferating germ cells here and in images C-E. The germline produces oocytes at this stage; sperm were made earlier and stored in the spermatheca (not shown). Red box marks region imaged (~ 5 µm maximum intensity images presented). Modified from [2]. (c-e) Representative

brightfield images of extruded gonads from worms grown at 20°C. Scale bar, 10 µm. Worms were examined three times with similar results. Similar germline phenotypes were observed in the confocal images throughout this study. (c) Wild-type PGL-1::SNAP gonads are of normal size and produce oocytes and embryos. (d) Representative image of PGL-1(K126E K129E)::SNAP sterile gonads, which are small and produce no gametes. (e) PGL-1(R123E)::SNAP sterile gonads are also small and produce no gametes. (f-i) Representative partial z-projection stacks of SNAP (magenta) and DNA (DAPI, cyan) stained germlines. Scale bar, 10 µm, applies for all images. (f,g) PGL-1::SNAP mutant protein is expressed in sterile gonads (no embryos observed). (f) PGL-1(K126E K129E)::SNAP, n=21 gonads imaged. (g) PGL-1(R123E)::SNAP, n=30 gonads imaged. (h,i) In single rare gonads, PGL-1::SNAP mutants were seen to assemble into granules in all germ cells. The molecular phenotype was observed only once in each mutant, despite the imaging experiments were performed 3 times.

Supplementary Figure 7. PGL-1 protein expression. (a-d) Examples of SNAP staining in (a) PGL-1::SNAP, (b) wild-type N2 negative control, (c) PGL-1(K126E K129E)::SNAP and (d) PGL-1(R123E)::SNAP. DNA (DAPI) in cyan and SNAP in magenta. Imaging experiments were performed three times with similar results. Scale bar (white), 10 µm. (e) Fluorescent signal (y-axis, arbitrary units) from SNAP-stained PGL-1::SNAP (red), N2 (blue), PGL-1(K126E K129E)::SNAP (green) and PGL-1(R123E)::SNAP (purple) germlines were measured from the beginning of the germline (start of progenitor zone, Supplementary Fig. 6b) to the meiotic pachytene region (x-axis, µM). Solid line represents the mean with black showing the standard error. (f,g) Statistics of fluorescence signal curve comparisons in (e). Each distance position was compared independently. (f) P-values comparing N2 versus GFP boxB reporter curves. N2 versus PGL-1::SNAP in purple, N2 versus PGL-1(R123E)::SNAP in blue, and N2 versus PGL-1(K126E K129E)::SNAP in yellow. (g) P-values comparing GFP boxB reporter strain curves. Statistics for f-g were calculated by multiple unpaired t-tests, with a 1.00% False Discovery Rate (FDR) by a two-stage step-up (Benjamini, Krieger, and Yekutieli). PGL-1::SNAP versus PGL-1(K126E K129E)::SNAP in black. PGL-1::SNAP versus PGL-1(R123E)::SNAP in magenta. PGL-1(K126E K129E)::SNAP versus PGL-1(R123E) in light blue. (h) Immunoblot of embryo-containing adult, N2, PGL-1::SNAP or PGL-1(R123E)::SNAP expressing worms. Samples were separated by SDS-PAGE and probed with SNAP and actin antibodies. Experiment was repeated twice with similar results.

Supplementary Figure 8. Targeted RNAi screen reveals WAGO-1 as a potential candidate in repressing PGL-tethered mRNA transcripts
(a) Candidate gene RNAi screening strategy. PGL-1::SNAP::λN22 tethered to GFP reporter mRNAs repress GFP expression. We propagated larval worms on bacteria expressing RNAi for specific P granule-associated enzymatic factors. After 5 days (one generation), young adult worm progeny were observed for GFP nuclei in their germlines, indicating tethering de-repression. N-terminal Dimerization domain (NtDD, yellow), Central Dimerization Domain (CDD, orange), SNAP (magenta), λN22 (light blue), and RGG repeats (blue), and GFP (green). Modified from [3]. (b) Candidate genes and their effect on GFP expression. Sequences of candidate genomic regions reported. Results were repeated at least three times and summarized here. (c) *wago-1* (R06C7.1.1) primary transcript. 5’ and 3’ UTRs are grey, exons are white, numbered 1-9 and separated by introns. Site of 3xV5 insertion and deleted region of null allele noted.

Supplementary Figure 9. PGL-1-tethered GFP reporter transcripts localize to granules in the presence or absence of WAGO-1
Gonads were extruded from *gfp* reporter, PGL-1::SNAP::λN22 animals harboring (a-d) *wago-1* wild-type (n=22); (e-h) *wago-1* null (n=21). Gonads were fixed and imaged for (a,e) *gfp* RNA using smFISH (yellow), (b,f) GFP protein fluorescence (green), (c,g) DNA (DAPI, cyan) and SNAP

(magenta). *gfp* RNA smFISH, DNA and SNAP merged in d, h. Black arrows mark examples of intranuclear puncta; white arrows mark examples of cytoplasmic puncta. Scale bar, 5 µm, for all images, except for 2.5-fold enlarged images in inset. For germline location, see Supplementary Fig. 6b. n= biologically independent animals examined over 2 independent experiments. (i) Quantification of GFP signal in confocal images for the GFP boxB reporter expressed with PGL-1::SNAP::λN22 with (n=22) or without (n=21) WAGO-1. Mean GFP fluorescent signal (line) and standard deviation reported in arbitrary units. GFP signal was modestly greater germlines without WAGO-1 (*, p-value = 0.0249). n= biologically independent animals (3 technical replicates) examined over 2 independent experiments. A two-tailed t test was used to calculate the p-value. (j) smFISH and PGL-1::SNAP signal colocalization in germlines expressing the GFP reporter, PGL-1::SNAP::λN22 in the presence or absence of WAGO-1. Reported as the (Sum of smFISH GFP Intensity in SNAP:smFISH colocalized binned signal)/(Total smFISH GFP binned signal). Co-localization ratios are modestly greater in germlines expressing PGL-1::SNAP::λN22 with WAGO-1 (*, p-value = 0.0390). (k) Total smFISH signal in germlines expressing the boxB GFP reporter, PGL-1::SNAP::λN22 in the presence or absence of WAGO-1. Total smFISH signal was greater in germlines expressing PGL-1::SNAP::λN22 without WAGO-1 (**, p-value = 0.001). Box plots represent the first and third quartiles, black line is median, whiskers to min and max values. Two-tailed t tests were used to calculate significance between smFISH data. See Methods for more details. (l) Example image of Imaris drawn colocalization volumes for PGL-1 and smFISH. PGL-1 granules in pink, gfp RNA granules in yellow, colocalized granules in green. Note how the digital, square edges increased the boundaries outside of the actual signal.

Supplementary Figure 10. GFP reporter expression in the presence or absence of WAGO-1. (a-c) Examples of GFP fluorescence in (a) negative control wild-type N2, (b) *wago-1(+)*; GFP reporter, and (c) *wago-1(-)*; GFP reporter. DNA (DAPI) in cyan and GFP in green. Scale bar (white), 10 µm. Imaging experiments were performed twice with similar results. (d) GFP Fluorescent signal (y-axis, arbitrary units) from germlines of described genotypes were measured from the beginning of the germline (start of progenitor zone, Supplementary Fig. 6b) to the meiotic pachytene region (x-axis, µM). Solid line represents the mean with black showing the standard error. Note that the GFP reporter expressed similarly with or without WAGO-1. N2 in blue, wago-1 (+); GFP boxB reporter in green, and wago-1 (-); GFP boxB reporter in purple. (e) Statistics of fluorescence signal curve comparisons in (d). P-values are reported for comparisons between N2 versus GFP boxB reporter, and GFP boxB reporter strains. N2 versus wago-1 (+); GFP boxB reporter in black, N2 versus wago-1 (-); GFP boxB reporter in pink, wago-1 (+); GFP boxB reporter versus wago-1 (-); GFP boxB reporter in light blue. Statistics were calculated by multiple unpaired t-tests, with a 1.00% False Discovery Rate (FDR) by a two-stage step-up (Benjamini, Krieger, and Yekutieli). Note that in the comparison between *wago-1(+);* GFP boxB reporter and *wago-1(-);* GFP boxB reporter, only 2 of 972 distance positions had p-values <0.05.

Supplementary Figure 11. Additional images of PGL-1-tethered GFP reporter transcripts without WAGO-1

(a-f) Six additional examples of germlines harboring PGL-1::SNAP::λN22, *wago-1* null imaged for *gfp* RNA (yellow), DNA (DAPI, cyan), and SNAP (magenta). *gfp* RNA, DNA and SNAP merged in right images. Black arrows mark examples of intranuclear puncta; white arrows mark examples of cytoplasmic puncta. Scale bar, 5 µm, for all images, except for 2.5-fold enlarged images in inset. For germline location, see Supplementary Fig. 6b. Imaging experiments were performed twice with similar results.

Supplementary References:

1 Magis, C. *et al.* T-Coffee: Tree-based consistency objective function for alignment evaluation. *Methods Mol Biol* **1079**, 117-129, doi:10.1007/978-1-62703-646-7_7 (2014).

2 Crittenden, S. L., Troemel, E. R., Evans, T. C. & Kimble, J. GLP-1 is localized to the mitotic region of the C. elegans germ line. *Development* **120**, 2901-2911 (1994).
3 Aoki, S. T. *et al.* An RNA-Binding Multimer Specifies Nematode Sperm Fate. *Cell Rep* **23**, 3769-3775, doi:10.1016/j.celrep.2018.05.095 (2018).

Supplementary Table 1 Data collection and refinement statistics

	C. japonica NtDD, Selenomethionine (5W4D [https://www.rcsb.org/structure/5W4D])	*C. japonica* NtDD, Wild type (5W4A [https://www.rcsb.org/structure/5W4A])
**Data collection**		
Space group	C 1 2 1	C 1 2 1
Cell dimensions		
a, *b*, *c* (Å)	133.3, 94.8, 72.5	132.77, 94.67, 72.95
α, β, γ (°)	90, 91.4, 90	90, 90.8, 90
Resolution (Å)	48.47 - 1.599 (1.656 - 1.599)*	30.36 - 1.5 (1.554 - 1.5)
R_{merge}	0.0469 (0.8775)	0.08108 (1.282)
$I / \sigma I$	24.44 (2.30)	16.91 (1.85)
Completeness (%)	97.06 (95.16)	99.77 (99.33)
Redundancy	7.6 (7.2)	15.1 (13.6)
**Refinement**		
Resolution (Å)	48.470 - 1.599 (1.656 - 1.599)	30.360 - 1.500 (1.554 - 1.500)
No. reflections	115302 (11238)	143649 (14297)
R_{work} / R_{free}	0.1607 / 0.1925 (0.2590 / 0.2707)	0.1681 / 0.2039 (0.3038 / 0.3232)
No. atoms	7593	7785
Protein	6768	6813
Ligand/ion	168	136
Water	657	836
B-factors	30.58	30.21
Protein	29.29	28.97
Ligand/ion	53.64	51.75
Water	37.95	36.82
R.m.s. deviations		
Bond lengths (Å)	0.010	0.010
Bond angles (°)	1.00	0.99

One crystal for each structure. *Values in parentheses are for highest-resolution shell.

Supplementary Table 2. DNA Primers

name	gene(s) targeted	sequence	purpose
wago-1 sc1 F	*wago-1*	CTTTGTGTCATCATCACCAAATTGTTTC	wago-1 3xV5
wago-1 sc1 R	*wago-1*	catcaagtgtatgtggcatagctc	wago-1 3xV5
wago-1 sc2 F	*wago-1*	GTT CTC CAA TCT CGC TTA TTC GGT G	wago-1 null
wago-1 sc2 R	*wago-1*	CGA AAC GAT TTC TAA AAT GAA GCG ATG	wago-1 null
pgl-3 5.5 F	*pgl-3*	ctccggttaacgaatacaaagttagtg	pgl-3 3xV5
pgl-36b R	*pgl-3*	gttgaaatttgaaggcttaggaacctc	pgl-3 3xV5
glh-1 3-4 F1	*glh-1*	caactacagtaacctcgacacac	glh-1 3xmyc
glh-1 3-4 R1	*glh-1*	caaatccagaaccgccagtc	glh-1 3xmyc
prSA57	*pgl-3*	CTCCGGTTAACGAATACAAAGTTAGTG	pgl-3 3xFLAG, pgl-3 lambda 3xFLAG
prSA58	*pgl-3*	CAACCAATAAAACGTTGAAATTTGAAGGC	pgl-3 3xFLAG, pgl-3 lambda 3xFLAG
pgl-1 seq 5F	*pgl-1*	ttcctgccccgactaatcaatacaaag	pgl-1 SNAP
pgl-1 sc3 R	*pgl-1*	gttggagtttagaaacctccgcgtc	pgl-1 SNAP
B90F	wt	cattatatgacattgtcgaaatgtcc	wild type N2
B90R	wt	ttgtagttatacagaagaccgttacg	wild type N2
prAK349	*MosSCI reporter*	aagaggcagaatgtgaacaagactc	MosSCI insert
prAK169	*MosSCI reporter*	catgatgaattgaaatcatccc	MosSCI insert
pgl-1 sc6 F	*pgl-1*	gtccgaaatttgatgctgctgctg	pgl-1 R123E, pgl-1 K126E K129E
pgl-1 sc6 R	*pgl-1*	gtttcaacaacggcttgcagctatc	pgl-1 R123E, pgl-1 K126E K129E
PGL-3 His6 NotI R	*pgl-3*	gagtgcggccgcaagctttagtgatgatgatggtggtgcttcggtccctcgatcatcagc	PGL-3
NdeI PGL-3 F	*pgl-3*	gatatacatatggaagcaaacaaacgacaaattg	PGL-3
NdeI pgl-1 NIII F	*pgl-1*	CTCTGAATATTTTGTTTACTTTAAGAAGGAGATATACATATGGAGGCTAACAAGCGAG	PGL-1
JaPGL-1N Met1 F	*pgl-1*	GACATCGTCCAGATgTTGTTTCCGTCG	Cj PGL-1 methionine mutation
JaPGL-1N Met1 R	*pgl-1*	CGACGGAAACAAcATCTGGACGATGTC	Cj PGL-1 methionine mutation
JaPGL-1N Met2 F	*pgl-1*	GTTGGCCGATATCATgCGCAACGGTC	Cj PGL-1 methionine mutation
JaPGL-1N Met2 R	*pgl-1*	GACCGTTGCGcATGATATCGGCCAAC	Cj PGL-1 methionine mutation
PGL-3 K126E K129E F	*pgl-3*	tctcgatgctcgcccgaaaaacagaactaaaggaatcgaatgacgccaagattctgcaag	PGL-3 K126E K129E
PGL-3 K126E K129E R	*pgl-3*	CTT GCA GAA TCT TGG CGT CAT TCG ATT CCT TTA GTT CTG TTT TTC GGG CGA GCA TCG AGA	PGL-3 K126E K129E
3.1 NheI PGL-1 F	*pgl-1*	TAATACGACTCACTATAGGGAGACCCAAGCTGGATGGAGGCTAACAAGCGAGAAATTGTG	PGL-1 GFP
PGL-1 OLLAS R	*pgl-1*	TAAGGCGAGGCCCCAGTTCATTGGCGAATCCTGAGCCGAAACCTCCGCGTCCACCACGAC	PGL-1 GFP
OLLAS eGFP F	*eGFP*	AATGAACTGGGGCCTCGCCTTATGGGTAAAAGCGGGGGCATGGTGAGCAAGGGCGAGGAG	PGL-1 GFP
eGFP NotI R	*eGFP*	AAACGGGCCCTCTAGACTCGAGCGGCCTTACTTGTACAGCTCGTCCATG	PGL-1 GFP
PGL-1 K126E K129E F	*pgl-1*	CCG TAA GGC CGA GCT GAA GGA GAC TGA AGA CGC TAA G	PGL-1 K126E K129E
PGL-1 K126E K129E R	*pgl-1*	CTT AGC GTC TTC AGT CTC CTT CAG CTC GGC CTT ACG G	PGL-1 K126E K129E
PGL-3 R123E F	*pgl-3*	CTC GAT GCT CGC CGA AAA AAC AAA GCT AAA GAA G	PGL-3 R123E
PGL-3 R123E R	*pgl-3*	CTT CTT TAG CTT TGT TTT TTC GGC GAG CAT CGA G	PGL-3 R123E
PGL-1 R123E F	*pgl-1*	GAA TGC TCG CCG AAA AGG CCA AGC TGA AG	PGL-1 R123E
PGL-1 R123E R	*pgl-1*	CTT CAG CTT GGC CTT TTC GGC GAG CAT TC	PGL-1 R123E
NdeI PGL-1 NIII F	*pgl-1*	CTCTGAATATTTTGTTTACTTTAAGAAGGAGATATACATATGGAGGCTAACAAGCGAG	PGL-1
PGL-1 NIII His6 HindIII R	*pgl-1*	CTCGAGTGCGGCCGCAAGCTTTAGTGATGATGATGGTGGTGAGATTTCGGACCATCCAAC	PGL-1
pgl-1 opt R123E F	*pgl-1*	*GAATGCTTGCCGACAAGGCAAAGTTG*	PGL-1 R123E
pgl-1 opt R123E R	*pgl-1*	*CAACTTTGCCTTGTCGGCAAGCATTC*	PGL-1 R123E
pgl-1 opt K126E K129E F	*pgl-1*	*GTAAGGCAGACTTGAAGGACACCGAAGATG*	PGL-1 K126E K129E
pgl-1 opt K126E K129E R	*pgl-1*	*CATCTTCGGTGTCCTTCAAGTCTGCCTTAC*	PGL-1 K126E K129E
pET21a NdeI start F		*GAAATAATTTTGTTTAACTTTAAGAAGGAGATATACAT*	recombinant protein
PGL-1 opt 212 His6 R	*pgl-1*	*GTGGTGGTGCTCGAGTGCTTAATGATGATGATGATGATGTGACTTAGGCCCGTCCAGCAT*	PGL-1

Supplementary Table 3. CRISPR-Cas9 guide RNAs

Name	Type	Strain targeted	mutation	Sequence
glh-1 sgRNA 1	CRISPR-Cas9 sgRNA plasmid	N2	3xmyc	target sequence: TCCACTACCGAATCCAGTTT
pgl-3 sgRNAin1	CRISPR-Cas9 sgRNA plasmid	N2	3xV5	target sequence: GCAACGGAACGTCTGGAAG
pgl-3 crRNA 1	CRISPR-Cas9 RNA	N2	3xFLAG, 3xFLAG::λN22	target sequence: ACGTCTGGAAGaggctctta
pgl-1 crRNA 1	CRISPR-Cas9 RNA	N2	SNAP	target sequence: cccaccagttcagcttatgg
pgl-1 crRNA 5	CRISPR-Cas9 RNA	JK5687, JK5898, JK5874	K126E K129E	target sequence: gtcttcagtcttcttcagct
pgl-1 crRNA 8	CRISPR-Cas9 RNA	JK5687	R123E	target sequence: cttcttcagcttggccttac
SNAP crRNA 1	CRISPR-Cas9 RNA	JK5687	λN22	target sequence: CCTGGGCTGGGTCCTGCAGG
wago-1 crRNA 1	CRISPR-Cas9 RNA	N2	3xV5	target sequence: TGCGACTCCTTTGCTTCAAT
wago-1 crRNA 2	CRISPR-Cas9 RNA		null deletion	target sequence: ATTGGAGTCATAGCTCCTGG
wago-1 crRNA 3	CRISPR-Cas9 RNA		null deletion	target sequence: AAATCTGGGCAAGCGTACTG

Supplementary Table 4. CRISPR-Cas9 repair DNA oligos and PCR product

Name	Type	Strain targeted	mutation	Sequence	Enzyme screen
glh-1 3xmyc repair 1	ssDNA repair oligo	N2	3xmyc	ttccaccggttttattttgattaaaaactttatttcagCgAAAACTGGAAACGAACAGAAGCTTATTTCCGAGGAAGA<br>CCTCGCCGGAGAGCAAAAGCTCATCTCTGAAGAGGATCTTGGAGCCGAACAGAAGCTTATCTCT<br>GAAGAAGACCTCGGAGGATTCGGTAGTGGAGGCGGTTTCGGTGGTGGTAACAATGGAG	n/a
pgl-3 3xV5 repair 1	ssDNA repair oligo	N2	3xV5	agtttgccagcagcaacggaacCtcCggaCgaggcGGAAAGCCAATCCCAAACCCACTCCTCGGACTCGA<br>CTCCACCGGAGGAAAGCCAATCCCAAACCCACTCCTCGGACTCGACTCCACCATCGGAAAGCC<br>AATCCCAAACCCACTCCTCGGACTCGACTCCACCGGAtcttatggaggtggtcgcggtggcgatcgt	n/a
pgl-3 Lambda 3xFLAG repair R	ssDNA repair oligo	N2	3xFLAG::λN22	ACGATCGCCACCGCGACCACCTCCATAAGACTTGTCATCGTCATCCTTGTAATCGATGTCATGAT<br>CTTTATAATCACCGTCATGGTCTTTGTAGTCGGATCCTCCGTTGGCGGCCTTCCATTGGGCTTGCT<br>TCTCGGCACGACGCTCACGACGACGGGTACGGGCGTTTCCGCCTCTTCCAGACGTTCCGTTGCT<br>GCTGG	n/a
pgl-3 3xFLAG repair R	ssDNA repair oligo	N2	3xFLAG	ACGATCGCCACCGCGACCACCTCCATAAGACTTGTCATCGTCATCCTTGTAATCGATGTCATGAT<br>CTTTATAATCACCGTCATGGTCTTTGTAGTCGCCTCTTCCAGACGTTCCGTTGCTGCTGG	n/a
pgl-1 SNAP	PCR product	JK5687	SNAP	ggattcggtcaatttgctcccaccagttcagcttatggaAGTGGCGGTATGGACAAAGACTGCGAAATGAAGCGC<br>ACCACCCTGGATAGCCCTCTGGGCAAGCTGGAACTGTCTGGGTGCGAACAGGGCCTGCACCGTA<br>TCATCTTCCTGGGCAAAGGAACATCTGCCGCCGACGCCGTGGAAGTGCCTGCCCCAGCCGCCGT<br>GCTGGGCGGACCAGAGCCACTGATGCAGGCCACCGCCTGGCTCAACGCCTACTTTCACCAGCC<br>TGAGGCCATCGAGGAGTTCCCTGTGCCAGCCCTGCACCACCCAGTGTTCCAGCAGGAGAGCTTT<br>ACCCGCCAGGTGCTGTGGAAACTGCTGAAAGTGGTGAAGTTCGGAGAGGTCATCAGCTACAGCC<br>ACCTGGCCGCCCTGGCCGGCAATCCCGCCGCCACCGCCGCCGTGAAAACCGCCCTGAGCGGA<br>AATCCCGTGCCCATTCTGATCCCCTGCCACCGGGTGGTGCAGGGCGACCTGGACGTGGGGGGCT<br>ACGAGGGCGGGCTCGCCGTGAAAGAGTGGCTGCTGGCCCACGAGGGCCACAGACTGGGCAAGC<br>CTGGGCTGGGTCCTGCAGGCGGATCCggaggaggtggtcgcggaggatatggcggtggagaccgtg	n/a
pgl-1 K126E K129E repair	ssDNA repair oligo	JK5687, JK5898, JK5874	K126E K129E	tcgatgacgacaagaagctcggaatgctcgcccgtaaggcTGagctgaagGagactgaagacgctaagattcttcaagctcttct<br>caaagt	BlpI
pgl-1 R123E repair	ssDNA repair oligo	JK5687	R123E	tttctgtcatcgatgacgacaagaagctcggaatgctcgcTGAGaaggccaagctgaagaagactgaagacgctaagattcttc	HpyAV
PGL-1 SNAP lambda repair	ssDNA repair oligo	JK5687	λN22	ctccaccgccatatcctccgcgaccacctcctccGGATCCGTTGGCGGCCTTCCATTGGGCTTGCTTCTCGGC<br>ACGACGCTCACGACGACGGGTACGGGCGTTTCCAGAAGAGCCTGCAGGACCCAGCCCAGGCTT<br>GCCCAGTCTGTGGCCCTCGT	n/a
wago-1 3xV5 repair	ssDNA repair oligo	N2	3xV5	ATT TAT TTA TAT TTT GCA GGA CTA AAG GAC CAA TTG AAG GAA AGC CAA TCC CAA ACC<br>CAC TCC TCG GAC TCG ACT CCA CCG GAG GAA AGC CAA TCC CAA ACC CAC TCC TCG<br>GAC TCG ACT CCA CCA TCG GAA AGC CAA TCC CAA ACC CAC TCC TCG GAC TCG ACT<br>CCA CCG GAG CAA AGG AGT CGC AAG GCG AGC GCC TC	n/a
wago-1 deletion repair	ssDNA repair oligo	JK6158	null deletion	CCA ATG CCA CCA GTC ACT GCT CCA GGA TCA AAA CAT TGA AGT AAA CAA TCG AGA<br>ATA CAC	n/a

Supplementary Table 5. Imaris batch detection settings

	gfp smFISH channel: Figure S2L	PGL-1::SNAP channel: Figure S2L	*gfp* smFISH channel: Figure S9I	PGL-1::SNAP channel: Figure S9I
[Algorithm]				
Enable Region of Interest	FALSE	FALSE	FALSE	FALSE
Enable Region Growing	TRUE	TRUE	TRUE	TRUE
Enable Tracking	FALSE	FALSE	FALSE	FALSE
[Source Channel]				
Source Channel Index	4	5	4	5
Enable Smooth	TRUE	TRUE	TRUE	TRUE
Surface Grain Size	0.090233 (default -- 2x pixel size)	0.090233 (default -- 2x pixel size)	0.090241 (default)	0.090241 (default)
Enable Eliminate Background	TRUE	TRUE	TRUE	TRUE
Diameter of largest sphere	0.28000 µm*	0.60000 µm*	0.28000 µm	0.60000 µm
[Threshold]				
Enable Automatic Threshold	FALSE	FALSE	FALSE	FALSE
Manual Threshold value	2.0241**	5.66575**	3.08326	15.9772
Active Threshold	TRUE	TRUE	TRUE	TRUE
Enable Automatic Threshold B	TRUE	TRUE	TRUE	TRUE
Manual Threshold Value B	19.1165**	14.5472**	97.9303	67.4159
Active Threshold B	false**	false**	FALSE	FALSE
Region Growing Estimated Diameter	0.30000 µm*	0.40000 µm*	0.30000 µm	0.40000 µm
[Classify Seed Points]				
"Quality"	above 1.2526*	above 1.9952*	above 3.5559	above 15.052
[Classify Surfaces]				
"Number of Voxels Img = 1"	above 28.000*	above 28.000*	above 28.000	above 28.000

*Values were determined by trial and error, using values from measurements where possible.

**Manual threshold initially set by turning on Upper Threshold (threshold B) -- this changes the value for the automatic lower threshold. Set the lower threshold to lock that value, then turn off the upper threshold. This makes the Value B stored, although it isn't actually in use (active = false). Note: the same technique would provide different values for each image, so the 2.0241 was arbitrarily set on the image settings.

Supplementary Figure 1
Aoki, et al.

a ***pgl-1*** **(ZK381.4a.1)**

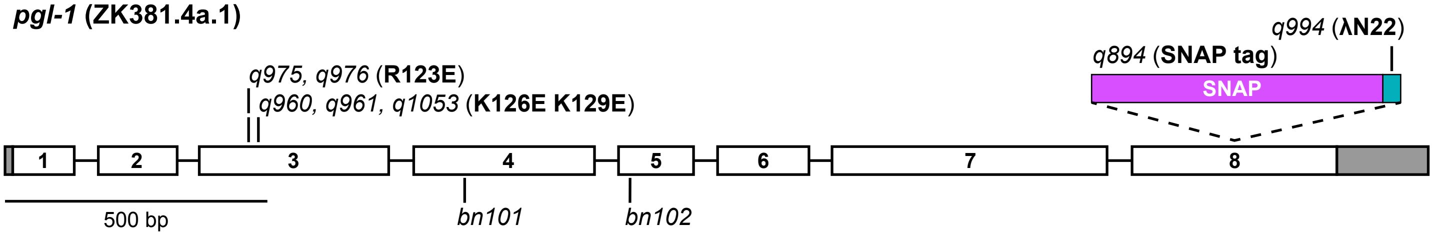

b ***pgl-3*** **(C18G1.4a.1)**

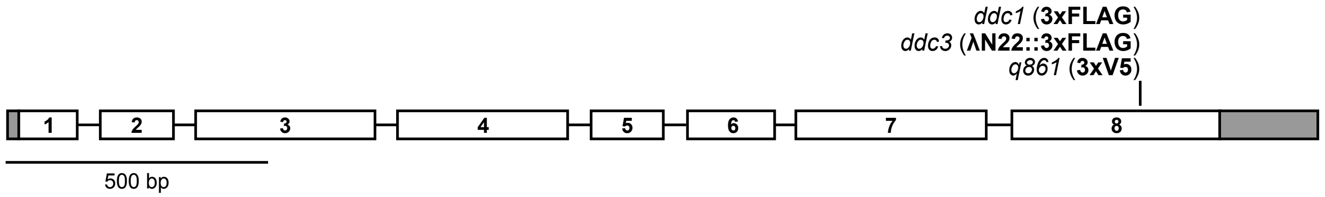

c

```
CePGL-1     1 MEANKREIVDFGGLRSYFFPNLAHYITKNDEELFNNTSQANKLAAFVLGASKDAPGDEDILEMILPNDANAAVIAAGMDV  80
CePGL-3     1 MEANKRQIVEVDGIKSYFFPHLAHYLASNDELLVNNIAQANKLAAFVLGATDKRPSNEEIAEMILPNDSSAYVLAAGMDV  80
CjPGL-1     1 MDTNKREIVEFLGIRTYFFPNLALYAVNNDELLVSDPNKANSFAAYVFGASDKKPSVDDIVQILFPSGSDSGTILTSMDT  80
CbnPGL-1    1 MEANKREIVETGGIKSYLFSNLAQYVTKNAELLQKTPKQANSLAAFVIGVSAERPTKDDILEMIIPNGANAAVLAAGMDV  80
CbrPGL-1    1 MELNKREIVEVGGIKSCFFPNLALYASKNSEALLNDPKSTNLFAANVFGALKDQPNENDITEMILPQDANADVLAAGMDA  80
CrPGL-1     1 MENNKRGVVEAKGIKSHYFQTLANYVSNNLELLHNNPKQANSFAASVFGSTAPI-DEKDLLDLLVPSDANADALAAGMDC  80
cons.         *: *** :*:  *:::  *  ** *  .* * * .   .:* :** *:*        .:: :::.*..:.: .: :.**

                                                            R123  K126  K129
CePGL-1    81 CLLLGDKFRPKFDAAAEKLSGLGHAHDLVSVID-----DDKKLGMLARKAKLKKTEDAKILQALLKV-IAIDDAAEKFVE 154
CePGL-3    81 CLILGDDFRPKFDSGAEKLSQLGQAHDLAPIID-----DEKKISMLARKTKLKKSNDAKILQVLLKV-LGAEEAEEKFVE 154
CjPGL-1    81 LLALGPDFLTEFKKRNQDLARFNLTHDLSILAQGDEDAAKKKLNLMGRKAKLQKTEAAKILAILIKT-INSEENYEKFTE 159
CbnPGL-1   81 CFLLGEEYRTNFQTAGEQLAQLNHSHDILAAVD-----DKKKLESLLRKTKIRKTPDAKILQRILTVHLEREEPLEKFEE 155
CbrPGL-1   81 CLLLGKQYHQLFESANERLSVLGRTHDLASIKD-----DEKKLTVLARRTKLKKTEGAKILQILIEA-IAEEDVFEKFMK 154
CrPGL-1    81 CLLLGEKYRPHFDAAVQQLARLGRTHDVATVID-----DEKKFTALSKKTKLKKTDEAKILQAFFKI-HST-EDEEKFEA 153
cons.          : ** .:   *.   : *: :. :**:    :      .**:  : :::*::*:  **** ::       :  ***

                                                             <-- NtDD domain |     | CDD domain -->
CePGL-1   155 LTELVSQLDLDFDVYVLTKILGLISEETSDEVDIIRDNVVNAFDSCKPLLKQLMLDGPK---SEPADPFISLLMDPL-EE 230
CePGL-3   155 LSELSSALDLDFDVYVLAKLLGFASEELQEEIEIIRDNVTDAFEACKPLLKKLMIEGPK---IDSVDPFTQLLLTPQ-EE 230
CjPGL-1   160 LSELC-GLDLDFDAYVFTKILGLEDEDTADEVEVIRDNFLNRLDQTKPKLADIIRNGPAVVELTPAEQFSRLLEVPVDES 238
CbnPGL-1  156 LSELC-ALDLDFDAYVLIKLLDIENEETAEEIEVVRENIQEIFQKANPLLKQLMEQGPQ---TEPADEFTELLRAPIGDD 231
CbrPGL-1  155 IAELC-SHDLDFDAYVLIKALGLECEEAVEEFKIIRENVLAVLKECNPLLSELLVDGPK---GAPVNEFTQLLLGSLSEE 230
CrPGL-1   154 ISELC-QLDLDFDAYVFIKALTLENEENQELVETIKDNLVEAWNKSNPLLVKLLLEGVKE--QDPVDKFTYLLLQPLTEA 230
cons.         ::**    *****.**: * * :  *:  : .. :::*.    .  :* * .:: :*       ..: *  **  .  :
```

Supplementary Figure 2
Aoki, et al.

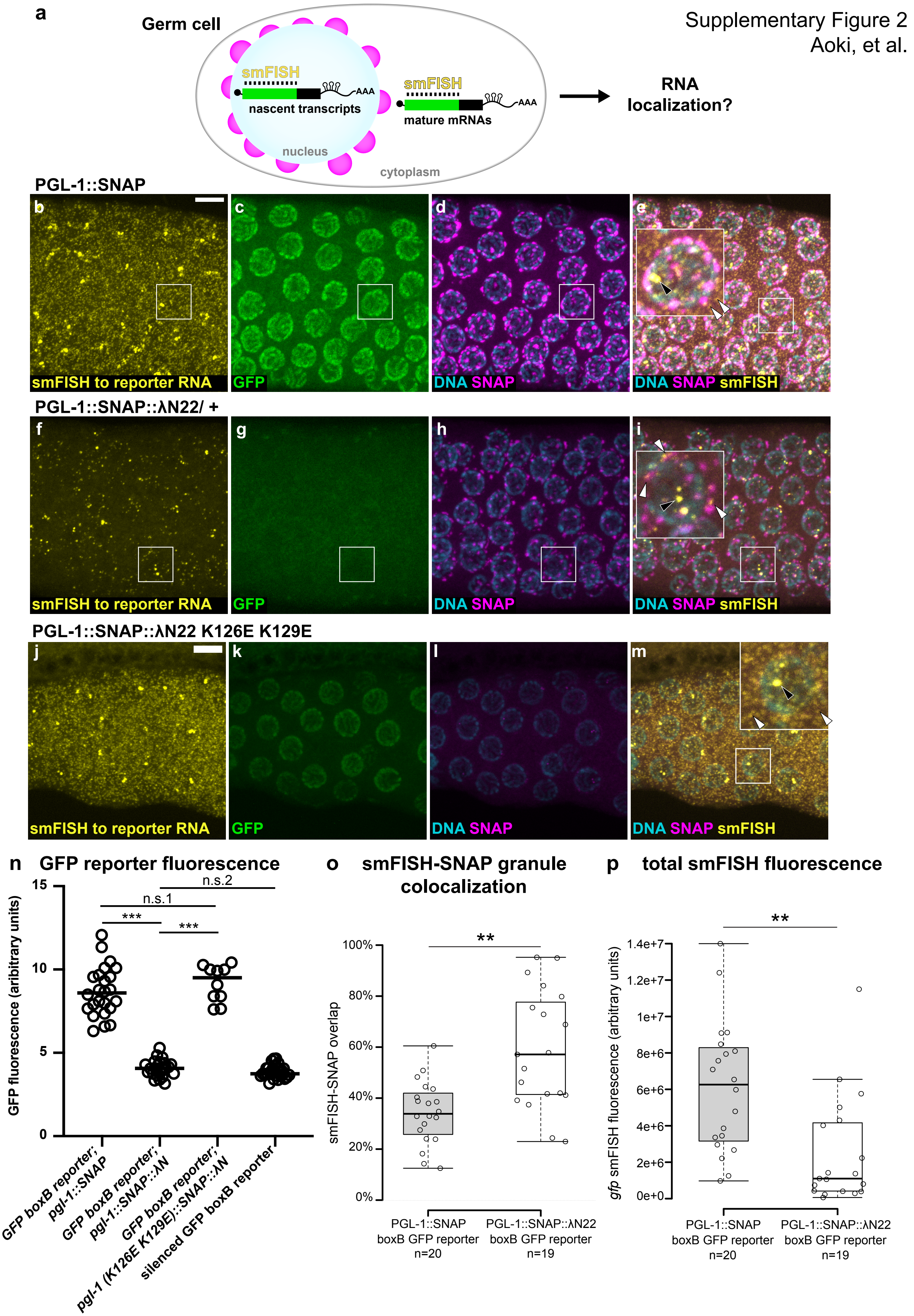

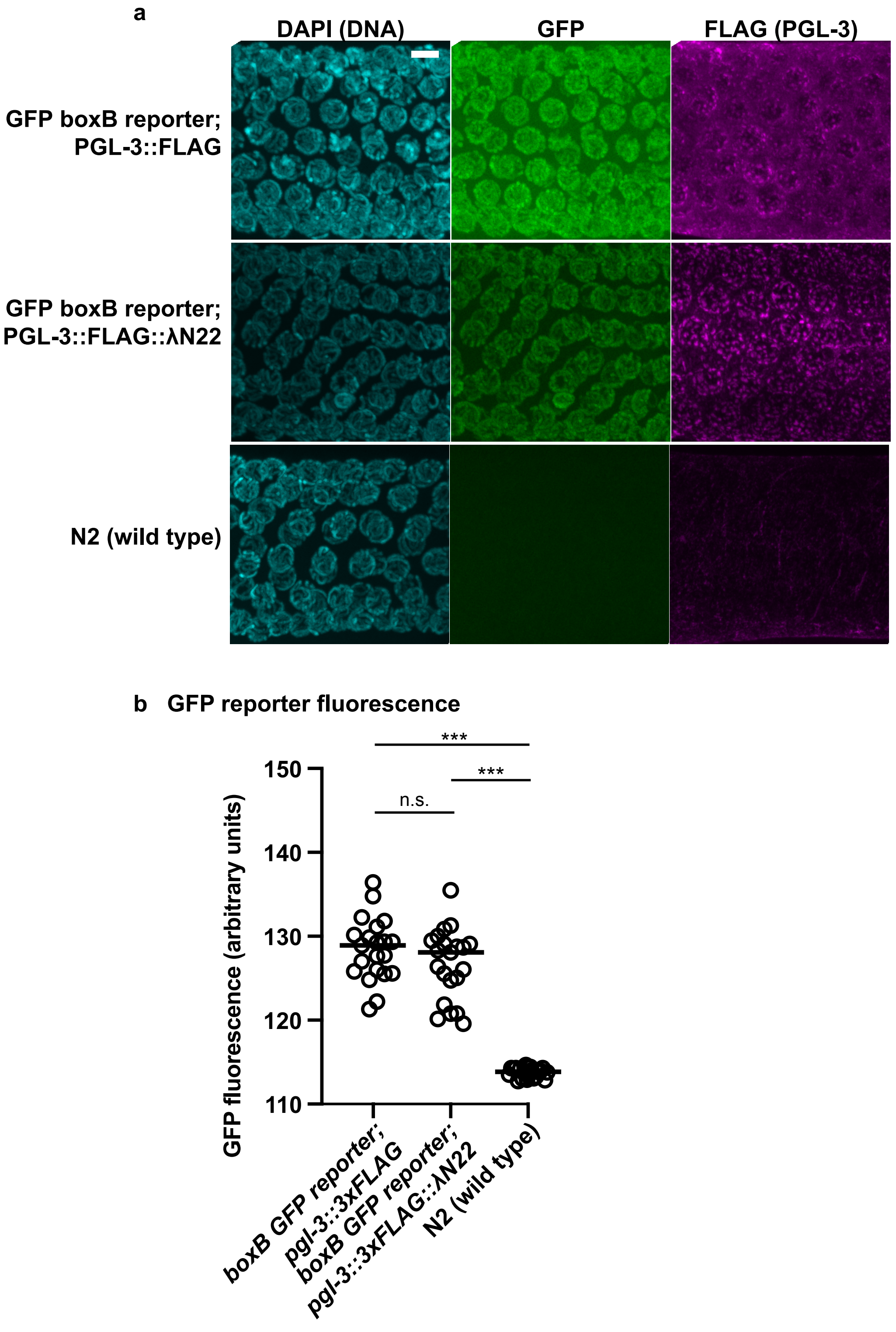
a
DAPI (DNA)
GFP
FLAG (PGL-3)
GFP boxB reporter; PGL-3::FLAG
GFP boxB reporter; PGL-3::FLAG::λN22
N2 (wild type)
b GFP reporter fluorescence
***
***
n.s.
150
140
130
120
110
GFP fluorescence (arbitrary units)
boxB GFP reporter; pgl-3::3xFLAG
boxB GFP reporter; pgl-3::3xFLAG::λN22
N2 (wild type)

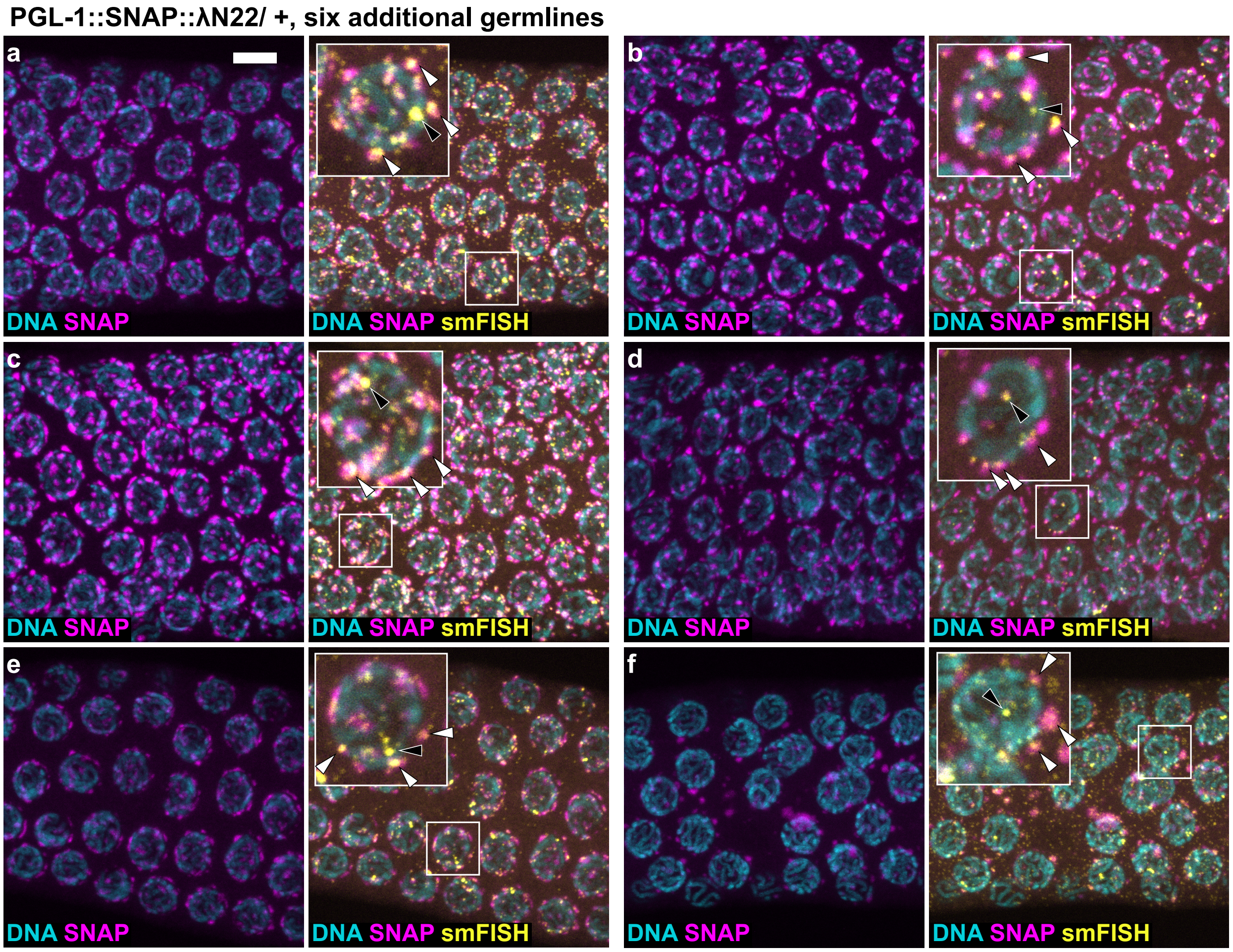
PGL-1::SNAP::λN22/ +, six additional germlines
a
DNA SNAP
DNA SNAP smFISH
b
DNA SNAP
DNA SNAP smFISH
c
DNA SNAP
DNA SNAP smFISH
d
DNA SNAP
DNA SNAP smFISH
e
DNA SNAP
DNA SNAP smFISH
f
DNA SNAP
DNA SNAP smFISH

Supplementary Figure 5
Aoki, et al.

PGL-1 and PGL-3 recombinant proteins

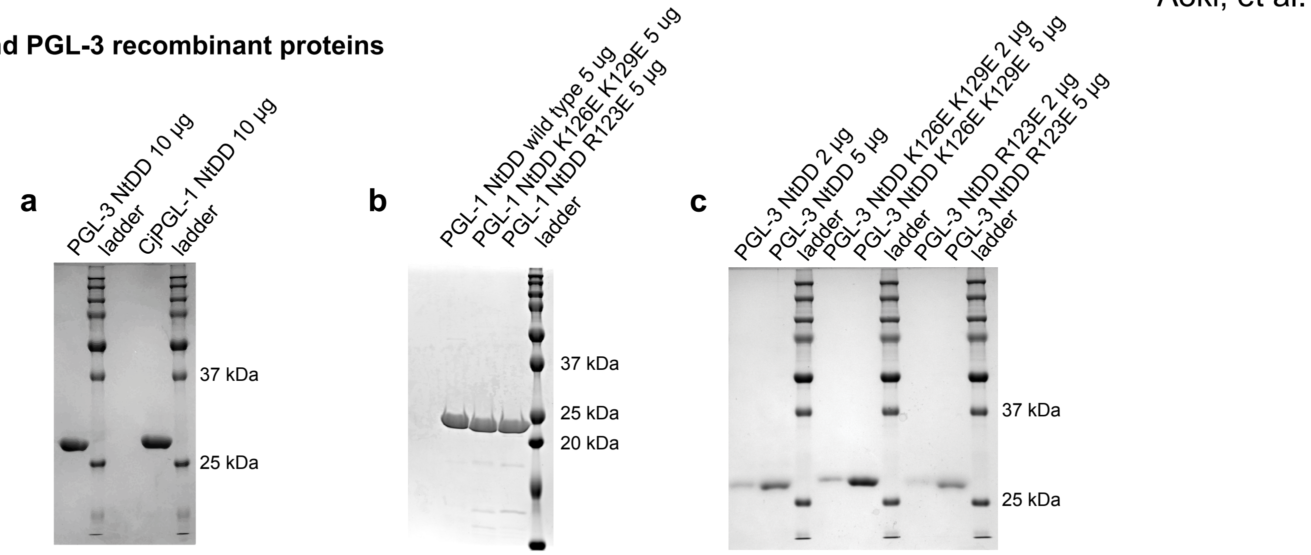

d *C. japonica (C. elegans)*

H-bonds

subunit 1	subunit 2
A:R128 (**R123**)	B:T158 (V153)
A:R128 (**R123**)	B:E189 (E185)
A:K131 (**K126**)	B:E162 (E157)
A:L132 (L127)	B:N196 (N192)
A:K134 (**K129**)	B:D167 (D163)
A:K134 (**K129**)	B:D169 (D165)
A:T158 (V153)	B:R128 (**R123**)
A:E162 (E157)	B:K131 (**K126**)
A:D167 (D163)	B:K134 (**K129**)
A:D169 (D165)	B:K134 (**K129**)
A:E189 (E185)	B:R128 (**R123**)
A:N196 (N192)	B:L132 (L127)

salt bridges

subunit 1	subunit 2
A:R128 (**R123**)	B:E189 (E185)
A:K131 (**K126**)	B:E162 (E157)
A:K134 (**K129**)	B:D167 (D163)
A:K134 (**K129**)	B:D169 (D165)
A:E162 (E157)	B:K131 (**K126**)
A:D167 (D163)	B:K134 (**K129**)
A:D169 (D165)	B:K134 (**K129**)
A:E189 (E185)	B:R128 (**R123**)

e

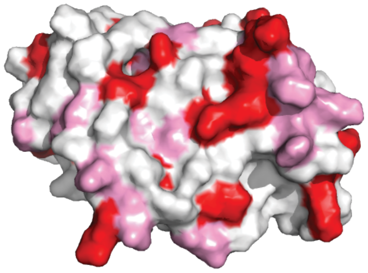

f

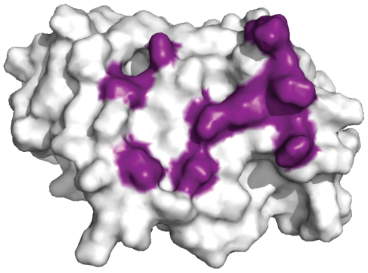

g

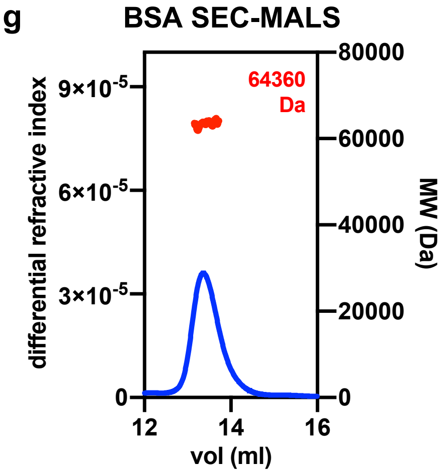

PGL-3 SEC-MALS:

h

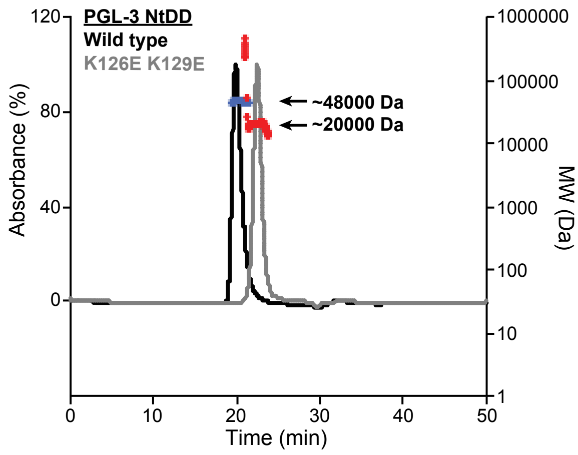

i

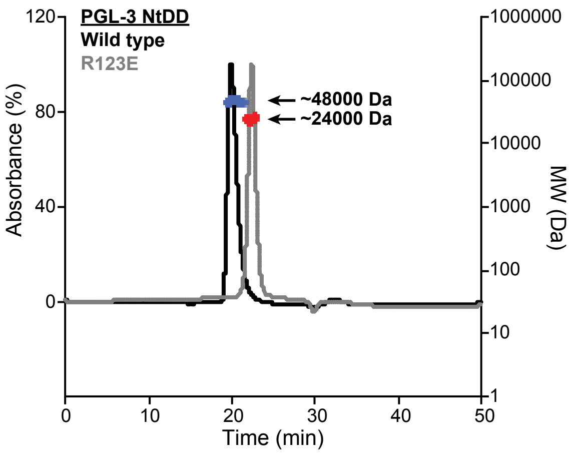

Supplementary Figure 6
Aoki, et al.

a

PGL-1 compared		alleles	p-value	<0.01?
**PGL-1(wt), 20°C**	**PGL-1(wt)::SNAP, 20°C**	+ v. *q894*	>0.9999	
	**PGL-1 null, 20°C**	+ v. *bn102*	0.2041	
	**PGL-1(wt), 25°C**	+ v. +	0.6171	
**PGL-1(wt)::SNAP, 20°C**	**PGL-1(K126E K129E)::SNAP, 20°C**	*q894* v. *q960*	<0.0001	***
	**PGL-1(R123E)::SNAP, 20°C**	*q894* v. *q975*	<0.0001	***
	**PGL-1(wt)::SNAP, 25°C**	*q894* v. *q894*	0.0346	
**PGL-1 null, 20°C**	**PGL-1 null, 25°C**	*bn102* v. *bn102*	<0.0001	***
**PGL-1(wt), 25°C**	**PGL-1(wt)::SNAP, 25°C**	+ v. *q894*	0.0999	
	**PGL-1 null, 25°C**	+ v. *bn102*	<0.0001	***
**PGL-1(wt)::SNAP, 25°C**	**PGL-1(K126E K129E)::SNAP, 20°C**	*q894* v. *q960*	<0.0001	***
	**PGL-1(R123E)::SNAP, 20°C**	*q894* v. *q975*	<0.0001	***

b

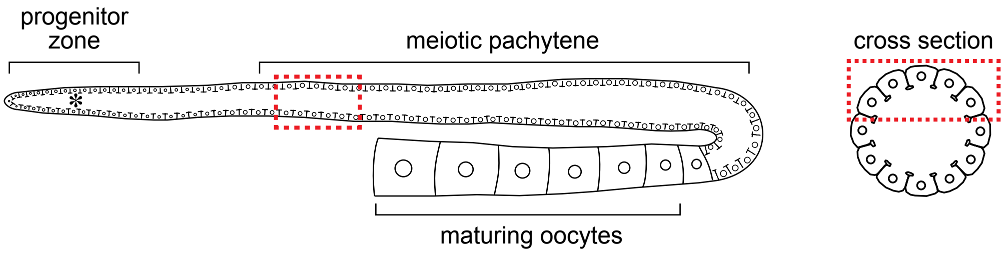

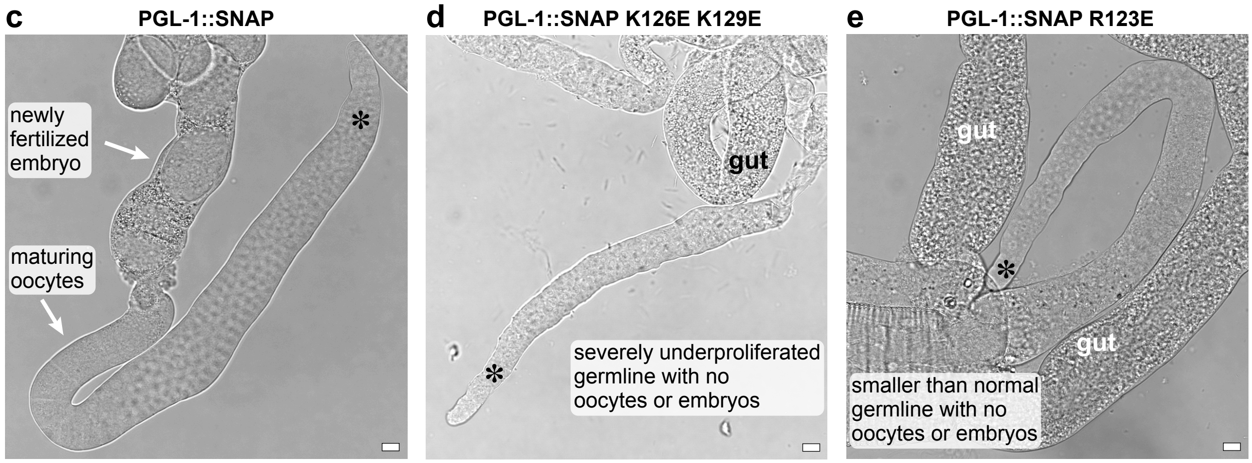

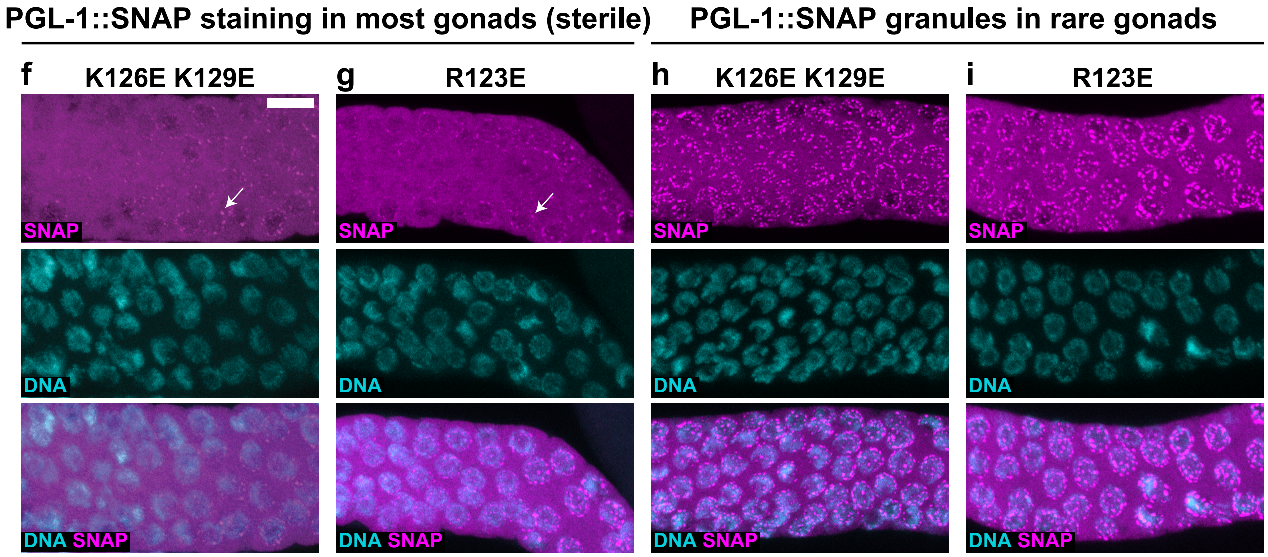

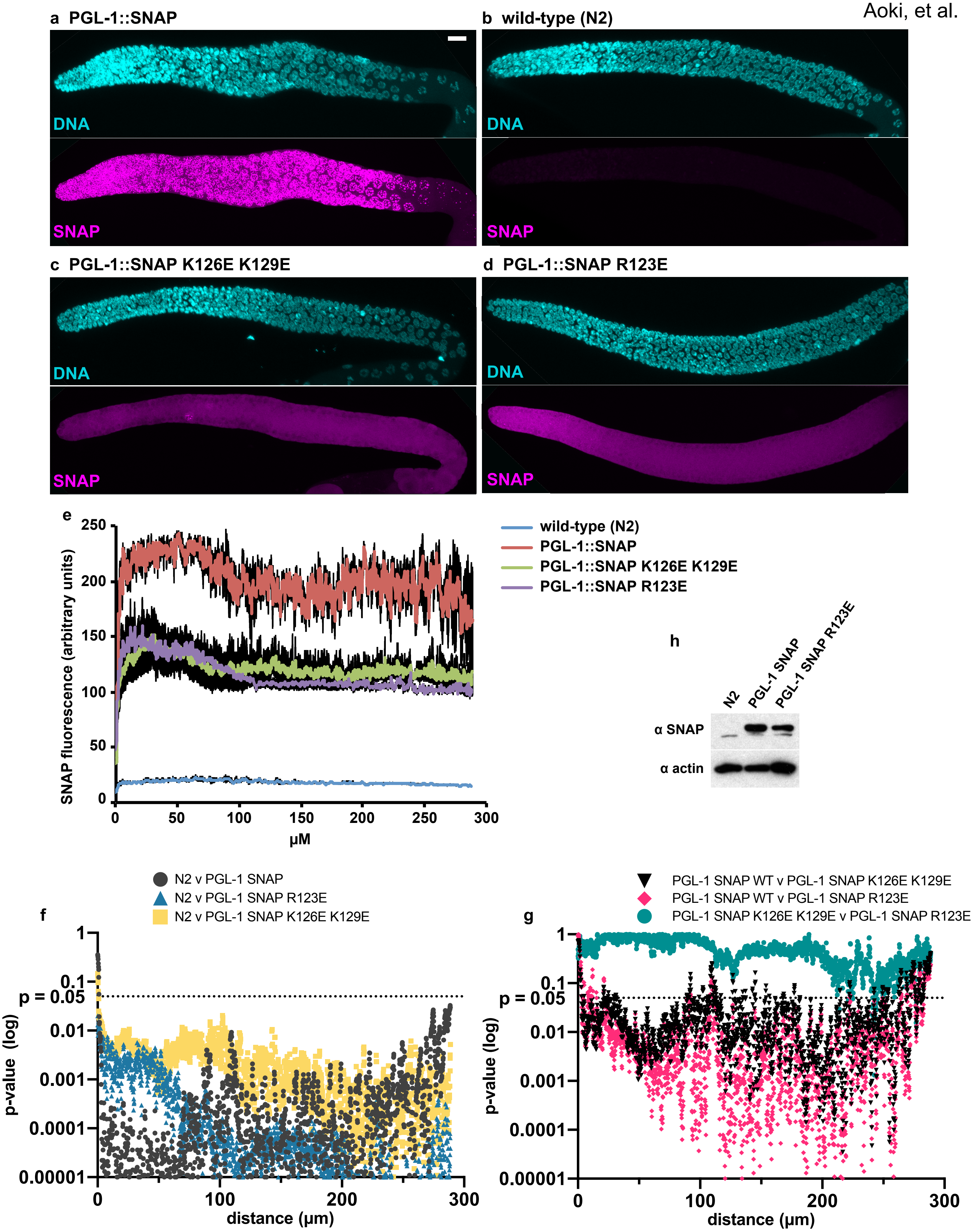
a PGL-1::SNAP
b wild-type (N2)
DNA
SNAP
c PGL-1::SNAP K126E K129E
d PGL-1::SNAP R123E
e
SNAP fluorescence (arbitrary units)
μM
wild-type (N2)
PGL-1::SNAP
PGL-1::SNAP K126E K129E
PGL-1::SNAP R123E
h
N2
PGL-1 SNAP
PGL-1 SNAP R123E
α SNAP
α actin
f
N2 v PGL-1 SNAP
N2 v PGL-1 SNAP R123E
N2 v PGL-1 SNAP K126E K129E
g
PGL-1 SNAP WT v PGL-1 SNAP K126E K129E
PGL-1 SNAP WT v PGL-1 SNAP R123E
PGL-1 SNAP K126E K129E v PGL-1 SNAP R123E
p = 0.05
p-value (log)
distance (μm)

a

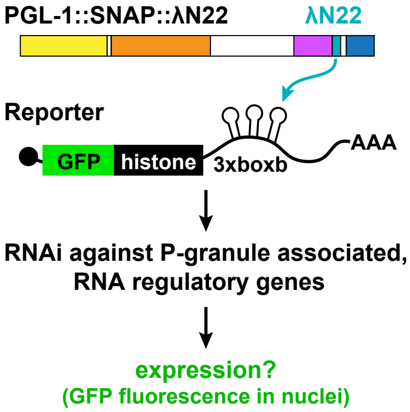

b

Gene	Sequence	Genome region (start...end)	GFP %	n
empty	–	–	0	204
csr-1	F20D12.1	CAGTACGCATTTCCTCGTGA...TTTACAGTCTTGCGTGCTGG	0	46
drh-3	D2005.5	CGCTCAACGTGTCAGTGATT...TCAAATGCTTCCATTGCAAG	0	59
ego-1	F26A3.3	ATCCGATCCGAACCATTACA...GGCATTTGGGATGAAAATTG	2.0	49
ekl-1	F22D6.6	ATGCAAAGCCAATTCCAGTC...GATTCAGGCGGTCGTGATAT	0	64
mut-2	K04F10.6	GCCTGGCACCAATGTAGTTT...CGTCCTCGTGATCCCTTAAA	0	70
parn-1	K10C8.1	TTCCGCCCAAATATTTCAAG...ATCAAGTCTTCATGGACGGC	0	46
pgl-1	ZK381.4	AATTTGTTCAAGGAATCAAC...GGAGAAGGTGTTACTGTAAAAGCG	0	64
prg-1	D2030.6	AGTGTGCTTTGCGATCTCCT...GACCACTTCCAGATGCCATT	1.4	69
rde-1	K08H10.7	CGACATCTGTTTCAGCAGGA...GATTGCGCTCCTCTGTTTTC	1.9	54
wago-1	R06C7.1	GGGAAGAGATTGACGTTGGA...AATCGGCTACAAACAAACCG	43.6	94
wago-4	F58G1.1	CATTGACGGTTGGTATTCAAAAT...TCAACAAGACCAATGTGAAGTTG	1.4	71

c ***wago-1*** **(R06C7.1.1)**

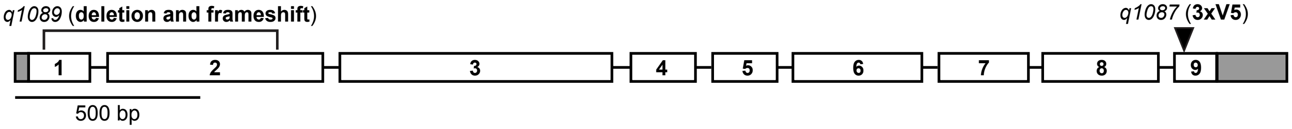

Supplementary Figure 9
Aoki, et al.

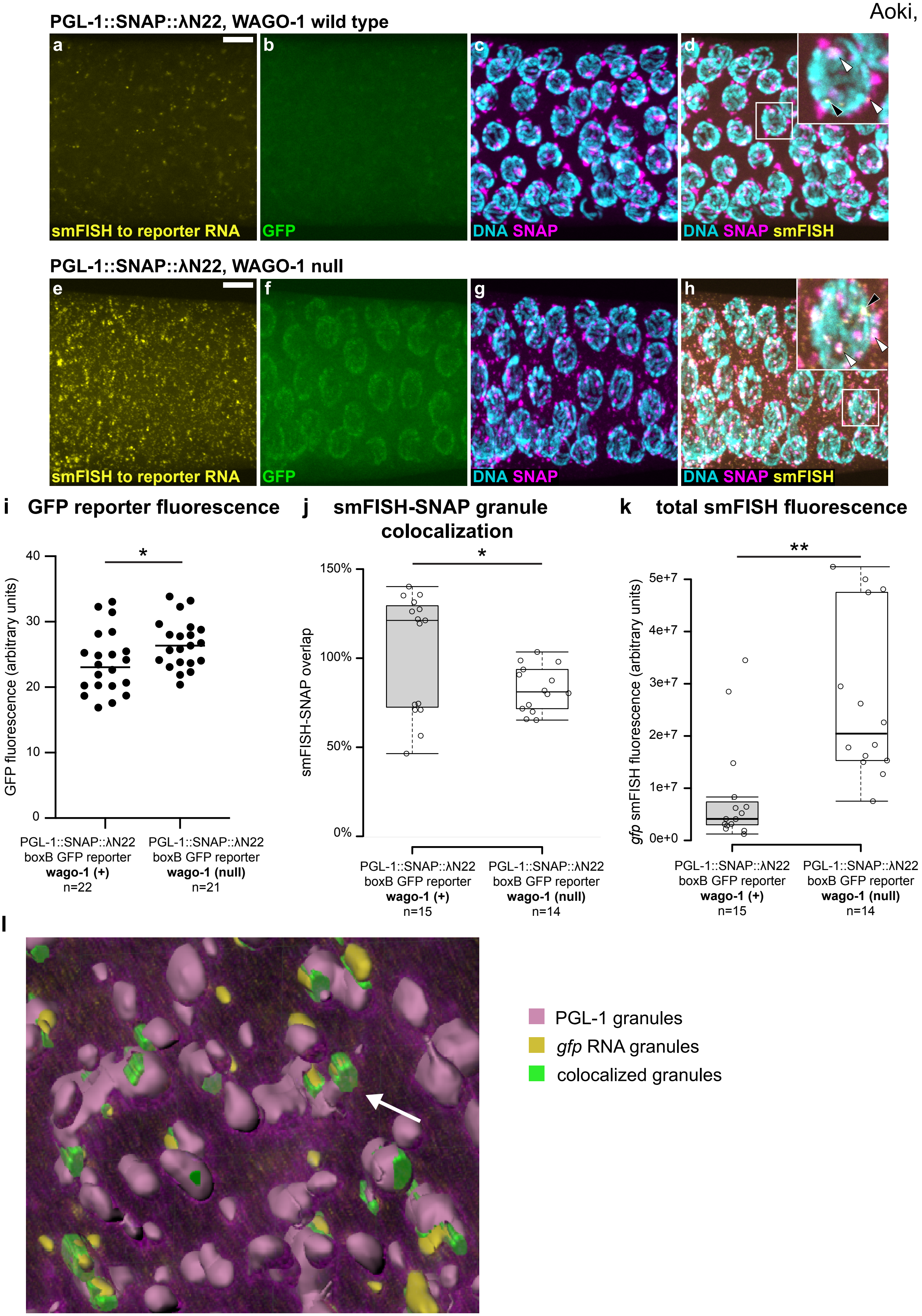

Supplementary Figure 10
Aoki, et al.

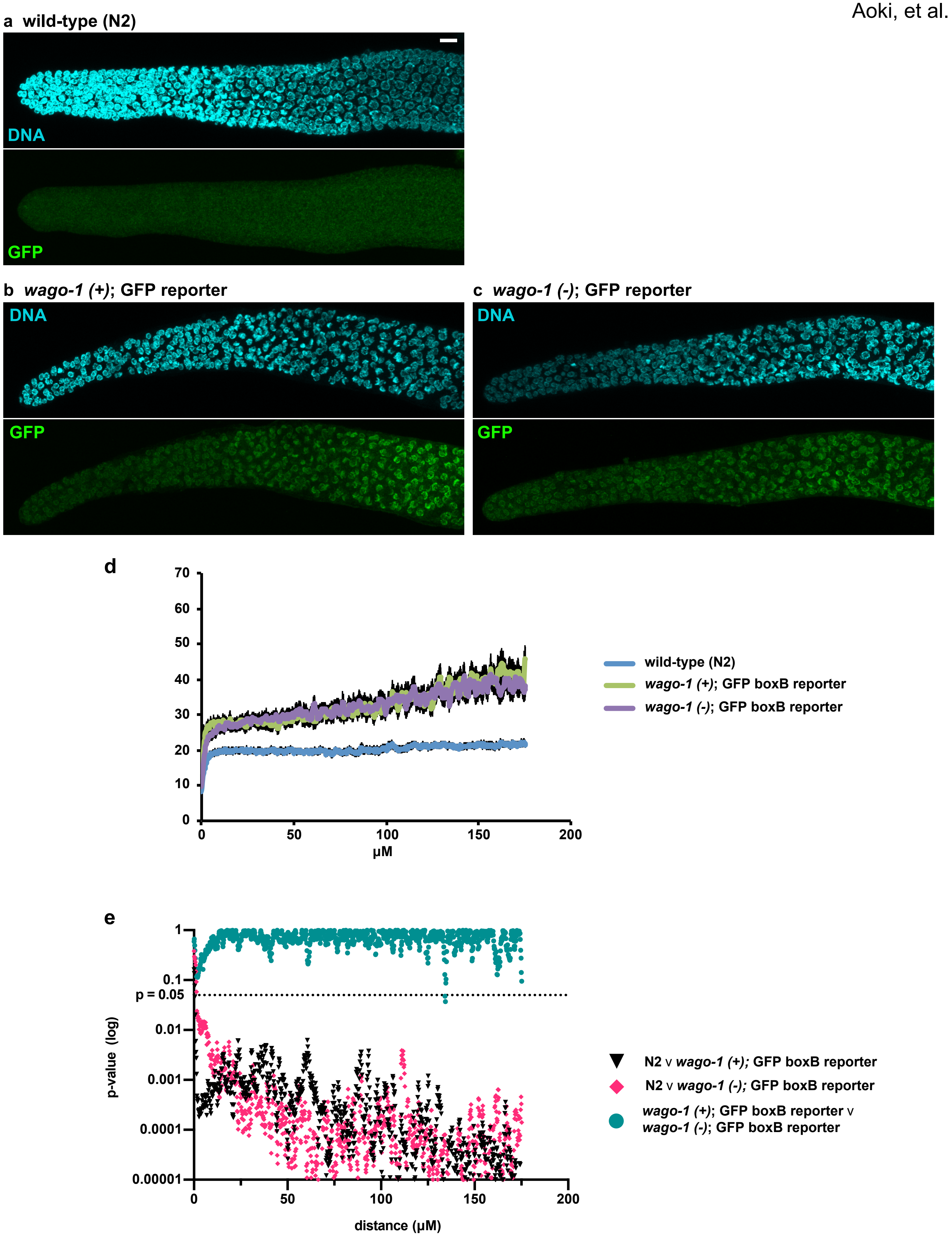

PGL-1::SNAP::λN22, WAGO-1 null, six additional germlines

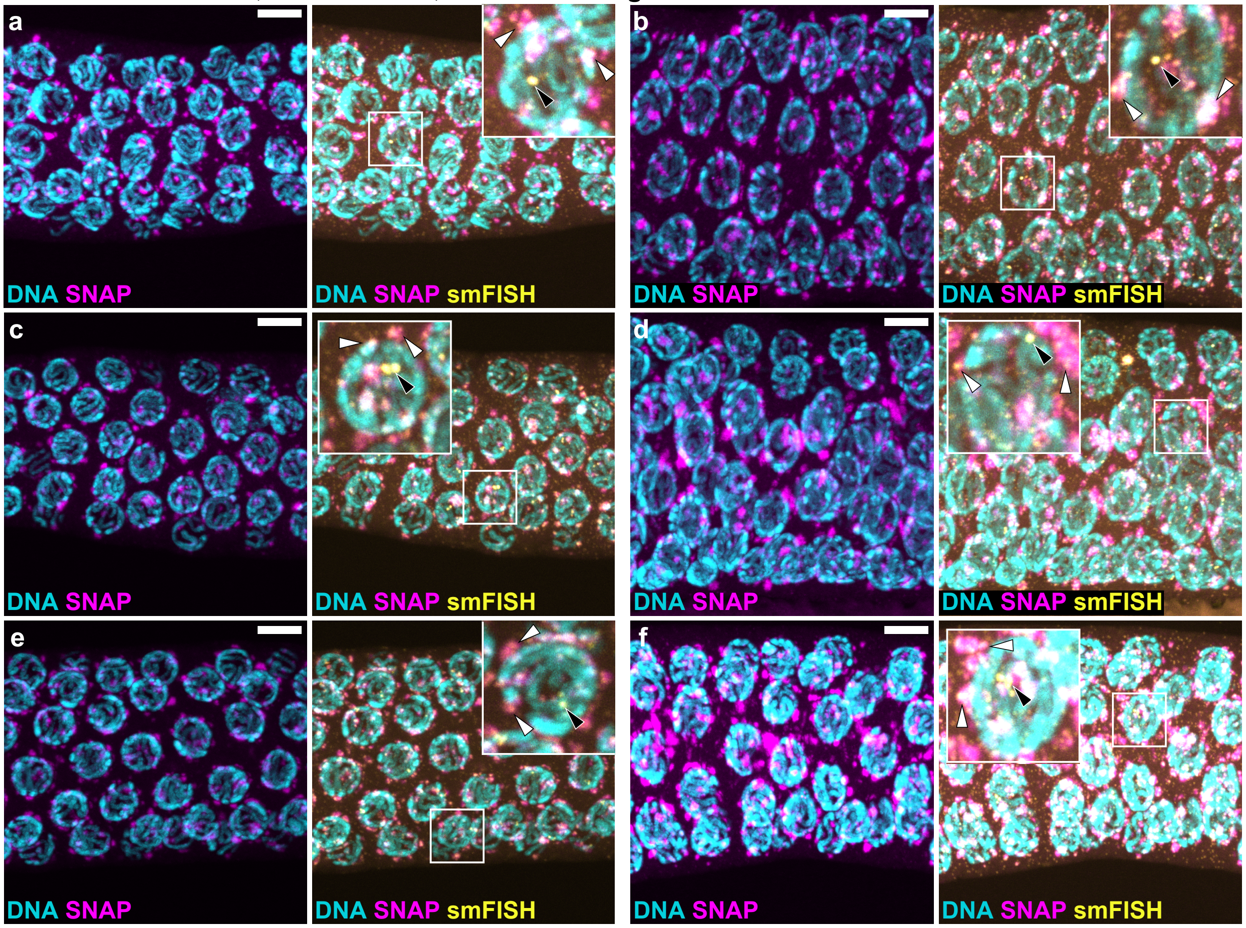

Supplement: Supplementary file 1 — Supplementary Information [file 41467_2021_21278_MOESM1_ESM.pdf]
