# Supplementary material for: C. elegans germ granules require both assembly and localized regulators for mRNA repression
Source: Nat Commun. 2021 Feb 12;12:996. doi: 10.1038/s41467-021-21278-1 (PMC7881195; doi:10.1038/s41467-021-21278-1)
Supplement: Supplementary file 3 — Reporting Summary [file 41467_2021_21278_MOESM3_ESM.pdf]

nature research

Corresponding author(s): Judith Kimble, Scott Aoki

Last updated by author(s): 12/14/2020

Reporting Summary

Nature Research wishes to improve the reproducibility of the work that we publish. This form provides structure for consistency and transparency in reporting. For further information on Nature Research policies, see our Editorial Policies and the Editorial Policy Checklist.

Statistics

For all statistical analyses, confirm that the following items are present in the figure legend, table legend, main text, or Methods section.

n/a	Confirmed	
☐	☒	The exact sample size (*n*) for each experimental group/condition, given as a discrete number and unit of measurement
☐	☒	A statement on whether measurements were taken from distinct samples or whether the same sample was measured repeatedly
☐	☒	The statistical test(s) used AND whether they are one- or two-sided<br>*Only common tests should be described solely by name; describe more complex techniques in the Methods section.*
☒	☐	A description of all covariates tested
☐	☒	A description of any assumptions or corrections, such as tests of normality and adjustment for multiple comparisons
☐	☒	A full description of the statistical parameters including central tendency (e.g. means) or other basic estimates (e.g. regression coefficient) AND variation (e.g. standard deviation) or associated estimates of uncertainty (e.g. confidence intervals)
☐	☒	For null hypothesis testing, the test statistic (e.g. *F*, *t*, *r*) with confidence intervals, effect sizes, degrees of freedom and *P* value noted<br>*Give P values as exact values whenever suitable.*
☒	☐	For Bayesian analysis, information on the choice of priors and Markov chain Monte Carlo settings
☒	☐	For hierarchical and complex designs, identification of the appropriate level for tests and full reporting of outcomes
☒	☐	Estimates of effect sizes (e.g. Cohen's *d*, Pearson's *r*), indicating how they were calculated

Our web collection on statistics for biologists contains articles on many of the points above.

Software and code

Policy information about availability of computer code

Data collection	Adobe Illustrator 2020 (24.3), FIJI/ImageJ (2.1.0/1.53c), Slidebook (6.0), Microsoft Excel for Mac (16.43.1) , Microsoft Word for Mac (16.41) , Phenix (1.12rc0-2798), Coot (0.8.2), Imarisx64 (9.3.1), MATLAB (R2018b), Prism 9 (9.0.0), LAS X (3.0.13), Pymol (2.4.0), ASTRA (ver. 6), Unicorn (5.1)
Data analysis	Bitplane XTension File Exchange [http://open.bitplane.com/tabid/235/Default.aspx?id=111]

For manuscripts utilizing custom algorithms or software that are central to the research but not yet described in published literature, software must be made available to editors and reviewers. We strongly encourage code deposition in a community repository (e.g. GitHub). See the Nature Research guidelines for submitting code & software for further information.

Data

Policy information about availability of data

All manuscripts must include a data availability statement. This statement should provide the following information, where applicable:

- Accession codes, unique identifiers, or web links for publicly available datasets
- A list of figures that have associated raw data
- A description of any restrictions on data availability

PDB IDs for structures published in this manuscript are 5W4A and 5W4D. The source data underlying Figures 1F-I, 3D-F, H-K, 4B, 5D-G, and 7D-G and Supplementary Figures S2N-P, S3B, S5A-C, G-I, S7E-H, S9I-K, S10D-E are provided as a Source Data file. All relevant data are available from the authors.

Field-specific reporting

Please select the one below that is the best fit for your research. If you are not sure, read the appropriate sections before making your selection.

- [x] Life sciences
- [ ] Behavioural & social sciences
- [ ] Ecological, evolutionary & environmental sciences

For a reference copy of the document with all sections, see nature.com/documents/nr-reporting-summary-flat.pdf

Life sciences study design

All studies must disclose on these points even when the disclosure is negative.

Sample size	All experiments were replicated at least twice to check for reproducibility. Sample sizes for worm imaging experiments were within ranges (~ 20 worm germlines) based on previous publications (e.g. DOI: 10.1534/genetics.115.185678). Fertility and cell culture imaging results were striking (e.g. +/- granules, 0 or ~100%), and n size was collected to be above a 95% confidence interval (5% error) for results < 5% or > 95%. Collective sizes (number) can be found in the figures and figure captions. Similar fertility counts were taken in all experiments and conclusions made emphasized a difference between genotypes, not specific values.
Data exclusions	PGL granule staining is sensitive to physical perturbation and some germlines lacked PGL-1::SNAP signal entirely or almost entirely. GFP smFISH and PGL-1::SNAP signal overlap was therefore calculated from the top 66% of images in terms of PGL-1::SNAP Intensity (Detailed tab, select Average Values from dropdown, "Intensity Sum Ch=5 Img=1" row and "Sum" column). Excluding the low PGL images increased median signal overlap from 26% to 34% in PGL-1::SNAP and from 41% to 57% in PGL-1::SNAP::λN22 (Figure S2O). Excluding the low PGL images increased median signal overlap from 113% to 121% in PGL-1::SNAP::λN22 with WAGO-1 and from 74% to 81% in PGL-1::SNAP::λN22 without WAGO-1 (Figure S9J). See Table S5 for the Imaris settings used. The excluded data is included in the Source Data file.
Replication	We define technical replicates as those samples performed in parallel with the same reagents, while biological replicates as samples performed on different days with similar reagents, depending on the experiment. Worm imaging, recombinant protein sizing column profiles and cell culture experiments were performed (data collected) at least twice. For the fertility experiments, worms were singled and checked for fertility once. Worm imaging experiments were done with the same strains, RNA probes and antibody reagents but with different animal generations. Recombinant protein experiments were done with at least two different protein preparations, with the cleanest preparations reported. Cell culture experiments were done using the same plasmid preparations and cell culture line, but they were performed on different days and transfections performed separately. All replicates were successful and reproduced similar data to those reported.
Randomization	Samples were organized by strains and their respective genotypes. Randomization was not relevant to this study. The experiments required knowledge of the strain genotypes to properly compare the measured results.
Blinding	Blinding was not performed nor relevant to this study, since similar genotypes had to be grouped to compare the effect of these genes on the phenotypes and molecular assays performed.

Reporting for specific materials, systems and methods

We require information from authors about some types of materials, experimental systems and methods used in many studies. Here, indicate whether each material, system or method listed is relevant to your study. If you are not sure if a list item applies to your research, read the appropriate section before selecting a response.

Materials & experimental systems

n/a	Involved in the study	
	x	Antibodies
	x	Eukaryotic cell lines
x		Palaeontology and archaeology
	x	Animals and other organisms
x		Human research participants
x		Clinical data
x		Dual use research of concern

Methods

n/a	Involved in the study	
x		ChIP-seq
x		Flow cytometry
x		MRI-based neuroimaging

Antibodies

Antibodies used

anti-MYC (JAC6 (rat), Bio-Rad (Hercules, CA; MCA1929)
anti-V5 (sv5-Pk1 (mouse), Bio-Rad, Hercules, CA; MCA1360)
Alexa 488 Donkey anti-Mouse (Donkey anti-Mouse IgG (H+L) Highly Cross-Adsorbed Secondary Antibody, Alexa Fluor 488 Invitrogen, Carlsbad, CA; A21202)
Alexa 555 Donkey anti-Mouse (Donkey anti-Mouse IgG (H+L) Highly Cross-Adsorbed Secondary Antibody, Alexa Fluor 555 Invitrogen, Carlsbad, CA; A31570)
Alexa 647 Donkey anti-Mouse (Donkey anti-Mouse IgG (H+L) Highly Cross-Adsorbed Secondary Antibody, Alexa Fluor 647

Invitrogen, Carlsbad, CA; A31571)
Alexa 488 Goat anti-Rabbit (Goat anti-Rabbit IgG (H+L) Highly Cross-Adsorbed Secondary Antibody, Alexa Fluor 488
Invitrogen, Carlsbad, CA; A11008)
Anti-FLAG (M2 (mouse), Millipore-Sigma; Burlington, MA; F1804-1MG)
Anti-SNAP (Anti-SNAP-tag® Antibody (Polyclonal) (rabbit), New England BioLabs, Ipswich, MA; F1804-1MG)
Anti-actin (ACTN05) (C4 (mouse), Novus Biologicals, Centennial, CO; NB600-535)
Goat anti mouse Horseradish peroxidase (Goat anti-Mouse IgG (H+L) Secondary antibody, Centennial, CO; NB600-535)

Validation

Anti-V5 antibody: readily detects epitope-tagged proteins (PMID: 10357214);
Anti-MYC antibody: readily detects epitope-tagged proteins (e.g. PMID: 12077606)
Anti-FLAG antibody: readily detects epitope-tagged proteins in C. elegans (DOI: 10.1534/genetics.115.182592)
Anti-SNAP antibody: readily detects epitope-tagged proteins in C. elegans (DOI: 10.1038/s41592-018-0017-z)
Anti-actin (C4) antibody: readily detects actin in C. elegans (DOI 10.1534/genetics.115.182592)

Eukaryotic cell lines

Policy information about cell lines

Cell line source(s)	Chinese Hampster Ovary (CHO) cells (American Type Culture Collection, ATCC)
Authentication	The cell line was directly ordered from ATCC but not directly tested.
Mycoplasma contamination	Cell lines were not tested for mycoplasma contamination.
Commonly misidentified lines (See ICLAC register)	No commonly misidentified cell lines were used in the study.

Animals and other organisms

Policy information about studies involving animals; ARRIVE guidelines recommended for reporting animal research

Laboratory animals

Caenorhabditis elegans
Sex: hermaphrodites
Age: analyzed worms were approximately 24 hours past the mid-L4 larval stage, as determined by vulva development
Strains used:
N2 bristol
JK5687: pgl-1(q894)[PGL-1::SNAP] IV
JK5902: pgl-1(q975)[PGL-1(R123E)::SNAP] IV
JK6158: wago-1(q1087)[WAGO-1::3xV5]; pgl-1(q894)[PGL-1::SNAP] IV
JK6159: wago-1(q1089)[WAGO-1(null deletion)::3xV5]; pgl-1(q894)[PGL-1::SNAP] IV
JK6157: wago-1(q1087)[WAGO-1::3xV5]; pgl-1(q975)[PGL-1(R123E)::SNAP] IV
JK5898: glh-1(q858)[GLH-1::3xMYC] I; pgl-1(q894)[PGL-1::SNAP] IV; pgl-3(q861)[PGL-3::3xV5] V
JK5970: qSi375[(mex-5 promoter::eGFP::linker::his-58::3xboxb::tbb-2 3'UTR) *weSi2] II; pgl-1(q894)[PGL-1::SNAP] IV
JK5873: qSi375[(mex-5 promoter::eGFP::linker::his-58::3xboxb::tbb-2 3'UTR) *weSi2] II; pgl-1(q994)[PGL-1::SNAP::λN22]/nT1[qIs51] (IV;V)
JK5874: qSi375[(mex-5 promoter::eGFP::linker::his-58::3xboxb::tbb-2 3'UTR) *weSi2] II; pgl-1(q994)[PGL-1::SNAP::λN22]/nT1[qIs51] (IV;V)
JK6149: qSi375[(mex-5 promoter::eGFP::linker::his-58::3xboxb::tbb-2 3'UTR) *weSi2] II; pgl-1(q994)[PGL-1::SNAP::λN22]/nT1[qIs51] (IV;V)
JK6150: qSi375[(mex-5 promoter::eGFP::linker::his-58::3xboxb::tbb-2 3'UTR) *weSi2] II; pgl-1(q994)[PGL-1:SNAP:λN22]/nT1[qIs51] (IV;V)
JK6147: wago-1(q1089)[WAGO-1(null deletion)::3xV5]; qSi375[(mex-5 promoter::eGFP::linker::his-58::3xboxb::tbb-2 3'UTR) *weSi2] II; pgl-1(q994)[PGL-1::SNAP::λN22]/nT1[qIs51](IV;V)
JK6148: wago-1(q1089)[WAGO-1(null deletion)::3xV5]; qSi375[(mex-5 promoter::eGFP::linker::his-58::3xboxb::tbb-2 3'UTR) *weSi2] II; pgl-1(q994)[PGL-1::SNAP::λN22]/nT1[qIs51](IV;V)
JK6367: qSi375[(mex-5 promoter::eGFP::linker::his-58::3xboxb::tbb-2 3'UTR) *weSi2] II
JK6368: wago-1(q1089)[WAGO-1(null deletion)::3xV5]; qSi375[(mex-5 promoter::eGFP::linker::his-58::3xboxb::tbb-2 3'UTR) *weSi2] II
CDE15: qSi375[(mex-5 promoter::eGFP::linker::his-58::3xboxb::tbb-2 3'UTR) *weSi2] II; pgl-3(ddc1)[PGL-3::3xFLAG] V
CDE16: qSi375[(mex-5 promoter::eGFP::linker::his-58::3xboxb::tbb-2 3'UTR) *weSi2] II; pgl-3(ddc3)[PGL-3::λN22::3xFLAG] V

Worm strains that could not be frozen:
1. pgl-1(q960)[PGL-1(K126E K129E)::SNAP] IV
2. glh-1(q858)[GLH-1::3xMYC] I; pgl-1(q960)[PGL-1(K126E K129E)::SNAP] IV; pgl-3(q861)[PGL-3::3xV5] V
3. qSi375[(mex-5 promoter::eGFP::linker::his-58::3xboxb::tbb-2 3'UTR) *weSi2] II; pgl-1(q1053)[PGL-1(K126E K129E)::SNAP::λN22]/nT1[qIs51](IV;V)

Wild animals	No wild animals were used in the study.
Field-collected samples	No field collected samples were used in the study.

Ethics oversight	The study did not require an ethical approval.

Note that full information on the approval of the study protocol must also be provided in the manuscript.
