# Supplementary material for: C. elegans germ granules require both assembly and localized regulators for mRNA repression
Source: Nat Commun. 2021 Feb 12;12:996. doi: 10.1038/s41467-021-21278-1 (PMC7881195; doi:10.1038/s41467-021-21278-1)
Supplement: Supplementary file 2 — Peer Review File [file 41467_2021_21278_MOESM2_ESM.pdf]

Reviewers' comments:

Reviewer #1 (Remarks to the Author):

Summary
P granules, the C. elegans germ granules, have essential roles for the development and maintenance of germ line. However, the molecular basis of their assembly and function still remains elusive. In a previous study, the authors identified a domain in PGL-3 (CDD) that has a capacity to dimerize in vitro (Aoki et al, 2016). They also found that an equivalent domain in PGL-1 possesses a guanosine-specific endonuclease activity (Aoki et al, 2016). In this following study, the authors identified an additional domain in the N-terminal region of PGL-3 (NtDD), which also has a capacity to dimerize in vitro. When conserved K or R residues in this domain were replaced with E residue (K126E, K129E, R123E), the "mutated" PGL-3 NtDD domain lost the capacity to dimerize in vitro. In addition, when the equivalent amino-acid substitutions were introduced into PGL-1, the "mutated" PGL-1 failed to form granular structures in mammalian cultured cells. When these "mutated" PGL-1 proteins were expressed in C. elegans hermaphrodite gonads, these "mutated" PGL-1 proteins mostly failed to assemble into P granules and caused highly penetrant sterility similar to, but even severer than, a pgl-1 null mutant. On the other hand, using a protein-mRNA tethering assay, the authors demonstrated that a reporter mRNA expression was repressed when this mRNA was tethered to PGL-1 and, as a result, localized to P granules. To examine whether this mRNA repression depends on the recruitment of this mRNA to P granules, this reporter mRNA was tethered to the "mutated" PGL-1 that is incapable of assembling into P granules because of the K to E substitutions. In this way, the authors smartly demonstrated that when the reporter mRNA is not assembled into P granules, repression of this mRNA expression does not occur. Finally, the authors found that this repression of mRNA expression requires WAGO-1, a P-granule associated Argonaute that functions in the 22G-endo-siRNA pathways in the germ line. This manuscript is well-thought, nicely designed, and described mostly based on solid experimental evidences they presented, and should certainly contribute to the community for further understanding of the molecular basis of assembly and function of PGL-1, PGL-3, and P granules. However, this reviewer requests the authors to revise the manuscript on the following points before acceptance for publication.

General comments
(1) Please describe "P granules" instead of "P-granules" through the manuscript when this term is used as a "noun" because that has been the original manner of description in the original papers (for example, Kawasaki et al, 1998, 2004). The description "P-granules" is sometimes used in recent papers but this is a confusion possibly caused by its similarity to "P-bodies". On the other hand, when it is used as an "adjective", it is fine to describe with a hyphen such as "P-granule assembly".

(2) Usage of the terms "PGL" and "PGLs": In this manuscript, the terms "PGL" and "PGLs" are often used instead of PGL-1 and/or PGL-3. For example, although the authors presented the results of mRNA tethering experiments using only PGL-1, they concluded that "tethered PGL represses a reporter mRNA in nematode germ cells". This description can be an overstatement. The authors should either present results of equivalent experiments using also PGL-3 or "PGL" should be corrected to "PGL-1" for accuracy. Through this study, PGL-1 appears to be used in the majority of experiments. PGL-3 appears to be used only in the experiments shown in Fig. 3. "PGL" and "PGLs" should be used only when the description (conclusion) is supported by the experimental results that were examined using both PGL-1 and PGL-3. The authors should be careful on this point because although PGL-1 and PGL-3 are members of the same protein family and function redundantly, at least respective single mutant phenotypes are not identical (Kawasaki et al, 2004).

(3) The title: Recently, the liquid droplet-like nature of P granules or PGL-1 has been intensively studied. However, in this study, the authors did not present any data showing how these dimerization domains (CDD and NtDD) actually affect and contribute to the "liquid droplet" nature of PGL-1 and/or PGL-3. For example, experiments examining the effect of amino-acid substitutions in the DD domain

on the liquid droplet nature of PGL-1 protein, as examined by Zhang et al (2018), are not presented in this study. In this sense, it appears not relevant to include the term "liquid droplet" in the title, unless this aspect is actually investigated, and the data are presented in this study.

Specific comments
Also, please respond to the following comments:
(1) For the protein-mRNA tethering assay: The authors arbitrarily selected to insert the "boxBs" into the 3'UTR of the reporter mRNA to tether this mRNA to PGL-1. Many of previous studies have shown that binding of various RNA-binding proteins to their target mRNAs via their 3'UTR results in repression of target mRNA expression. On the other hand, it has not been really proven for mRNAs that associate with P granules in nature, whether these associations always occur via their 3'UTR. There may also exist a class of germline mRNAs that associate with P granules via their 5'UTR or even via coding sequences. Notably, other classes of P-granule associated Argonautes, PRG-1 and ALG-3/4, appear to function for maintenance of robust spermatogenesis at high temperatures, suggesting that association of a class of spermatogenic mRNAs with P granules rather facilitate their expression rather than repression. Therefore, the authors should consider and mention a possibility in Discussion that the repression of reporter mRNA expression observed in this study might be affected by the design, that is, tethering this reporter mRNA to PGL-1 via the 3'UTR. In addition, because this protein-mRNA tethering assay is one of the key experiments in this study, it is strongly recommended to examine whether tethering of the reporter mRNA to "PGL-3" also repress the expression of a reporter mRNA so as to demonstrate that this is a common feature of PGLs or PGL granules.

(2) For the possible mechanism of mRNA repression: In Discussion, the authors described as, "the specific mechanism for mRNA repression, mRNA turnover or translational repression, is still unclear, although we favor RNA turnover driven by PGL's RNase activity and the Argonaute". In Fig. S2, when F is compared with B and J, not only the quantitative level of "GFP protein" but also the overall level of "gfp mRNA" appears to be reduced after tethering of the reporter mRNA to PGL-1. To further address the possible mechanism for mRNA repression, gfp mRNA levels should be quantified and compared among Fig. S2B, F, and J, by using either digital image analysis of smFISH fluorescence images or by qPCR analysis. If reduction of the level of gfp mRNA after tethering is confirmed, this result can further support the view that mRNA repression occurs through the "facilitation of mRNA turnover" rather than "repression of translation".

(3) For the P-granule localization of gfp mRNA: When Fig. S2E and I are compared, to this reviewer's eye, colocalization (overlap) frequency between gfp mRNA and PGL-1 (SNAP) does not look drastically increased by the addition of λN22 to PGL-1::SNAP (Fig. S2O). Why not all of (or even not the majority of) the gfp mRNA signals were co-localized with PGL-1::SNAP after addition of λN22 (Fig. S2I)? Further, why many gfp mRNA signals were still localized to P granules in the absence of λN22 in PGL-1::SNAP or after introduction of K126E and K129E substitutions into PGL-1::SNAP? Is there additional mechanism that localizes gfp mRNA to P granules independent of the boxBs-λN22 binding system? If there is such an additional mechanism that localizes gfp mRNA to P granules, why that localization mechanism does not repress the mRNA expression as the boxBs-λN22 binding system?

(4) For the P-granule localization of "mutated" PGL-1: Even after introduction of K126E and K129E substitutions or introduction of R123E substitution, these "mutated" PGL-1 proteins were still capable of localizing to P granules at least in a small fraction of gonads (Fig. S5E-H). Was this "minor" P-granule localization of "mutated" PGL-1 caused by an activity of another DD domain, CDD, in PGL-1 or by a redundant activity of NtDD in PGL-3? Or does PGL-1 contain a third domain for dimerization? What are the speculations of the authors?

(5) For the protein level of "mutated" PGL-1: Assuming that all the images were taken under the same conditions, it appears to this reviewer that immunofluorescence levels of PGL-1::SNAP were also reduced after introduction of K126E K129E or R123E substitution into PGL-1::SNAP in multiple Figures (C vs. E, F, and H vs. M in Fig. 4; A, D vs. B, C in Fig. 6; A vs. C, D, graph E, and western blot F in

Fig. S6). This reviewer requests the authors to quantify and compare the protein levels among PGL-1::SNAP, PGL-1(K126E K129E)::SNAP, and PGL-1(R123E)::SNAP in transgenic animals to answer this question. Possibly, one of the most straightforward approaches would be to reproduce a western immunoblot analysis as shown in Fig. S6F with addition of a protein sample obtained from PGL-1(K126E K129E)::SNAP animals. Then, band intensities of obtained immunoblots should be quantified, normalized, and statistically evaluated to answer this question. If reduction of PGL-1 protein levels after introduction of these substitutions is confirmed, how the authors would argue/explain this phenomenon? If these substitutions only affect dimerization or assembly of PGL-1, total protein level of PGL-1 in animals should not be changed. Recently, it was reported that ectopic expression of PGL proteins in somatic cells or a DNA damage-induced stress activated autophagy to degrade PGL proteins (Zhang et al, 2009, 2018; Min et al, 2019). Is there any possibility that the change of subcellular localization (diffusion to cytoplasm) or a structural change of PGL-1 caused by these substitutions (K126E K129E and R123E) might have induced autophagy to facilitate degradation of PGL-1?

(6) For the effect of "mutated" PGL-1 on the P-granule localization of PGL-3 (and GLH-1): As shown in Fig. 4N, O, P, not only PGL-1 but also the P-granule localization of PGL-3 was abolished by the K126E K129E substitution in PGL-1. (To this reviewer, it appears that the P-granule localization of GLH-1 was also, at least partially, abolished by this PGL-1 substitution like WAGO-1 in Fig. 6B, C.) Furthermore, it appears to this reviewer that protein levels of PGL-3 and GLH-1 were also reduced by this amino-acid substitution in PGL-1. Can the authors quantify and compare the protein levels of PGL-3 and GLH-1 between PGL-1::SNAP animals and PGL-1(K126E K129E)::SNAP animals using western blot analysis to make this point clearer? As authors mentioned in the manuscript, it was previously revealed that, although PGL-1 and PGL-3 could physically interact with each other, the absence of PGL-1 did not affect the localization of PGL-3 to P granules. Furthermore, although there was no direct protein-protein interaction between PGL-1 and GLH-1, whereas localization of GLH-1 to P granules was not affected by the absence of PGL-1, localization of PGL-1 to P granules was totally abrogated by the absence of GLH-1 in C. elegans germ line (Kawasaki et al, 2004). Therefore, the effect of this amino-acid substitution in PGL-1 and the effect of the absence of PGL-1 on the P-granule localization of PGL-3 (and possibly GLH-1) are quite contrasting. Because this is an interesting new finding, this reviewer appreciates if the authors try to explain how these contrasting effects are generated by this amino-acid substitution in PGL-1 compared with the absence of PGL-1 in Discussion. One more question is, was the same "dominant-negative" effect observed when another substitution, R123E, was introduced into PGL-1 on the P-granule localization of PGL-3 (and GLH-1)?

(7) For the effect of PGL-1 amino-acid substitutions on WAGO-1 expression and localization: In animals expressing PGL-1(R123E)::SNAP + WAGO-1::V5, the WAGO-1::V5 expression was diffuse and its localization to P granules was almost abrogated in about half of the germlines (Fig. 6B, C). Did the authors observe similar change of WAGO-1::V5 localization pattern in animals expressing PGL-1(K126E K129E)::SNAP + WAGO-1::V5 compared with animals expressing PGL-1::SNAP + WAGO-1::V5? Furthermore, protein level of WAGO-1 also appears to be reduced at least in half of the animals expressing PGL-1(R123E)::SNAP + WAGO-1::V5 compared with animals expressing PGL-1::SNAP + WAGO-1::V5 (Fig. 6A vs. 6B). The authors should quantify and compare the protein level of WAGO-1 between PGL-1::SNAP animals and PGL-1(R123E)::SNAP animals using western blot analysis, as requested for the quantification of PGL-3 and GLH-1 in a previous comment. If reduction of WAGO-1 protein level in PGL-1(R123E)::SNAP animals is confirmed, this result indicates that assembly of PGL-1 to P granules is critical for both P-granule localization and maintenance of protein level of WAGO-1, both of which should contribute to repress expression of mRNA localized to P granules.

(8) By using their RNAi screening system, did the authors examine (or will the authors examine) in more "wider" scale, whether any of the other endo-siRNA pathway players (not listed in Fig. S7B), which possibly function in the same pathway as WAGO-1, such as players functioning in the 22G-RNA pathway, are also required for the repression of P-granule localized mRNA so as to support the "mRNA

turnover" model?

Minor comments:
(1) Statistical significance of difference among values should be examined using a specified statistical method and should be addressed in Fig. S2N, S2O, S6F, and S9I.

(2) Why smFISH-SNAP overlap can exceed over 100% for wago-1 wild type in the Fig. S9I box plot?

(3) The description on the line 119, "PGL-1::SNAP with λN22 had GFP was faintly detected in only a few gonads" appears grammatically incorrect. At least, this reviewer could not understand the meaning well. Is this sentence means, for example, "In PGL-1::SNAP::λN22 animals, GFP was faintly detected in only a few gonads"? Please correct this sentence.

Reviewer #2 (Remarks to the Author):

The manuscript by Aoki et al. describes the identification of a second dimerization interface in PGL-1, and further tries to illustrate that mutations affecting dimerization alter PGL assembly and function.

This manuscript builds on prior work from the lab demonstrating the identification of a second dimerization domain (DD) in PGL proteins. Thus, PGL-1 and -3 contain not one, but two dimerization domains that appear to be essential for their assembly into P-granules.

Interestingly, among the four molecules in the asu, two interfaces were identified, though only one is reported to involve "conserved" amino acids. Although it is appreciated that residues involved in the NtDD interface are listed in Figure S4, it is common practice to report on buried surface area of all interfaces identified in the crystal structure, as was done by the authors in their 2016 publication in PNAS. It would also be helpful if the authors discussed how they believe the three mutations alter dimerization. If I am reading this correctly, the three residues are not listed as being involved in either hydrogen bonds or salt bridges, yet they are potent in their ability to disrupt dimerization, at least in the case of PGL-3 (Figure S4C). Is this through simple steric clashes or electronic repulsion?

For clarity, it would be helpful if more information were provided concerning the alignment of residues. The rmsd values are very low but it's unclear from what atoms this value was derived (all atom or alpha carbon), what residues (authors indicate not termini and loops-not clear what that does include?), and if refinement included symmetry constraints or not, and if so, on what residues. It would also be helpful if primary colors were used for distinguishing subunits. It is very difficult to clarify subunits in some images (Figure 2A, right panel).

It's unclear why mutations are being biochemically evaluated in PGL-3 while using PGL-1 as a model, as defined by the crystal structure. Ideally one should compare apples to apples and truly examine what is observed in the structure. Although I understand the two proteins share significant homology, since little information is given, an important question remains about the additional interface observed in the structure, which may or may not be present in PGL-3.

Responses to Reviewers' Comments:

REVIEWER 1

General comments
(1) Please describe "P granules" instead of "P-granules" through the manuscript when this term is used as a "noun" because that has been the original manner of description in the original papers (for example, Kawasaki et al, 1998, 2004). The description "P-granules" is sometimes used in recent papers but this is a confusion possibly caused by its similarity to "P-bodies". On the other hand, when it is used as an "adjective", it is fine to describe with a hyphen such as "P-granule assembly".

> **Response:** We thank the reviewer for this clarification and have made the requested change from "P-granule" to "P granules" throughout the revised manuscript.

(2) Usage of the terms "PGL" and "PGLs": In this manuscript, the terms "PGL" and "PGLs" are often used instead of PGL-1 and/or PGL-3. For example, although the authors presented the results of mRNA tethering experiments using only PGL-1, they concluded that "tethered PGL represses a reporter mRNA in nematode germ cells". This description can be an overstatement. The authors should either present results of equivalent experiments using also PGL-3 or "PGL" should be corrected to "PGL-1" for accuracy. Through this study, PGL-1 appears to be used in the majority of experiments. PGL-3 appears to be used only in the experiments shown in Fig. 3. "PGL" and "PGLs" should be used only when the description (conclusion) is supported by the experimental results that were examined using both PGL-1 and PGL-3. The authors should be careful on this point because although PGL-1 and PGL-3 are members of the same protein family and function redundantly, at least respective single mutant phenotypes are not identical (Kawasaki et al, 2004).

> **Response:** We thank the reviewer for this emphasizing this point and did the experiment to tether PGL-1. Indeed, PGL-1 and PGL-3 turned out to behave differently in the tethering assay (see below and manuscript). The revised manuscript includes this new experiment and has been changed throughout to specify that PGL-1, not PGL-3, regulates reporter transcripts. We also have now added text in the Introduction (lines 81-86) and Results (lines 148-153) to explain how our molecular findings correspond to previously published differences in the mutant phenotypes of these two proteins.

(3) The title: Recently, the liquid droplet-like nature of P granules or PGL-1 has been intensively studied. However, in this study, the authors did not present any data showing how these dimerization domains (CDD and NtDD) actually affect and contribute to the "liquid droplet" nature of PGL-1 and/or PGL-3. For example, experiments examining the effect of amino-acid substitutions in the DD domain on the liquid droplet nature of PGL-1 protein, as examined by Zhang et al (2018), are not presented in this study. In this sense, it appears not relevant to include the term "liquid droplet" in the title, unless this aspect is actually investigated, and the data are presented in this study.

> **Response:** "Liquid droplet" has been removed from the title.

Specific comments
Also, please respond to the following comments:
(1a) For the protein-mRNA tethering assay: The authors arbitrarily selected to insert the "boxBs" into the 3'UTR of the reporter mRNA to tether this mRNA to PGL-1. Many previous studies have

shown that binding of various RNA-binding proteins to their target mRNAs via their 3'UTR results in repression of target mRNA expression. On the other hand, it has not been really proven for mRNAs that associate with P granules in nature, whether these associations always occur via their 3'UTR. There may also exist a class of germline mRNAs that associate with P granules via their 5'UTR or even via coding sequences. Notably, other classes of P-granule associated Argonautes, PRG-1 and ALG-3/4, appear to function for maintenance of robust spermatogenesis at high temperatures, suggesting that association of a class of spermatogenic mRNAs with P granules rather facilitate their expression rather than repression. Therefore, the authors should consider and mention a possibility in Discussion that the repression of reporter mRNA expression observed in this study might be affected by the design, that is, tethering this reporter mRNA to PGL-1 via the 3'UTR.

> **Response:** We agree with the reviewer and have changed the revised manuscript accordingly. We have added text in the Discussion mentioning how the mode of mRNA binding may affect its regulation (lines 429-433).

(1b) In addition, because this protein-mRNA tethering assay is one of the key experiments in this study, it is strongly recommended to examine whether tethering of the reporter mRNA to "PGL-3" also repress the expression of a reporter mRNA so as to demonstrate that this is a common feature of PGLs or PGL granules.

> **Response:** As recommended, we did the requested experiment with PGL-3. Specifically, we used CRISPR/Cas9 to add a 3xFLAG or LambdaN22::3xFLAG tag to the endogenous *pgl-3* locus and tested the ability of PGL-3 to regulate the boxB GFP reporter. Figure S3 reports our results of this new experiment. Because, addition of the LambdaN22 to *pgl-3* had no effect on the boxB GFP reporter expression, we conclude that reporter repression is specific to tethered PGL-1, which provides a molecular basis to the phenotypic differences between *pgl-1* and *pgl-3* null mutants, first observed by Kawasaki, et al. (KAWASAKI *et al.* 2004). We incorporate this new finding into the Results (lines 131-133, 148-153, 1096-1098, 1129-1134) and Methods (lines 615-618, 651-652), and emphasize mechanistic differences between PGL-1 and PGL-3 in the Discussion (lines 427-429).

(2) For the possible mechanism of mRNA repression: In Discussion, the authors described as, "the specific mechanism for mRNA repression, mRNA turnover or translational repression, is still unclear, although we favor RNA turnover driven by PGL's RNase activity and the Argonaute". In Fig. S2, when F is compared with B and J, not only the quantitative level of "GFP protein" but also the overall level of "gfp mRNA" appears to be reduced after tethering of the reporter mRNA to PGL-1. To further address the possible mechanism for mRNA repression, gfp mRNA levels should be quantified and compared among Fig. S2B, F, and J, by using either digital image analysis of smFISH fluorescence images or by qPCR analysis. If reduction of the level of gfp mRNA after tethering is confirmed, this result can further support the view that mRNA repression occurs through the "facilitation of mRNA turnover" rather than "repression of translation".

> **Response:** We thank the reviewer for this suggestion and have now incorporated this quantitation into the relevant Supplemental Figures. Upon analysis of total *gfp* reporter mRNA, we find that 1) tethering reduces its abundance (Supp Fig S2P, p-value = 0.002), and that 2) loss of WAGO-1 increases its abundance (Supplemental Figures S9K, p-value = 0.001). The revised manuscript not only adds this quantitation in the Supplemental Figures and Results (lines 171-173, 338-342, 345-349, 1123-1127, 1215-1221, Figure S2P, Figure S9K), but also suggests in the Discussion that PGL-1 repression can facilitate mRNA turnover in a WAGO-1 dependent manner (lines 449-451, 1082-1083).

(3) For the P-granule localization of gfp mRNA: When Fig. S2E and I are compared, to this reviewer's eye, colocalization (overlap) frequency between gfp mRNA and PGL-1 (SNAP) does not look drastically increased by the addition of λN22 to PGL-1::SNAP (Fig. S2O). Why not all of (or even not the majority of) the gfp mRNA signals were co-localized with PGL-1::SNAP after addition of λN22 (Fig. S2I)? Further, why many gfp mRNA signals were still localized to P granules in the absence of λN22 in PGL-1::SNAP or after introduction of K126E and K129E substitutions into PGL-1::SNAP? Is there additional mechanism that localizes gfp mRNA to P granules independent of the boxBs-λN22 binding system? If there is such an additional mechanism that localizes gfp mRNA to P granules, why that localization mechanism does not repress the mRNA expression as the boxBs-λN22 binding system?

Response: Boundaries of PGL-1::SNAP granules were determined by Imaris, and mRNA smFISH signal within these boundaries were scored as co-localizing with the granule. The addition of LambdaN22 to PGL-1::SNAP led to an approximately two-fold higher recruitment of the reporter mRNA to P granules (from a mean of ~30% to a mean of ~60%). This co-localization was variable from germline to germline, as shown in Figure S2O, but a statistically significant difference (p-value = 0.0003) was found in co-localization with versus without the LambdaN22 tag.

We do not understand why we did not observe 100% recruitment of the reporter mRNA to P granules but suggest three plausible reasons. First, PGL granules are fluid, liquid droplets, as first demonstrated by Brangwynne, et al. in the embryo (BRANGWYNNE *et al.* 2009), and other groups with recombinant protein (SAHA *et al.* 2016; ZHANG *et al.* 2018; PUTNAM *et al.* 2019). The liquid nature allows PGL-1 protein to readily diffuse in and out of granules. That fluidity implies that mRNAs also can move in and out of P granules when bound by LambdaN22-tagged PGL-1. Second, P granules are unstable, as shown by Updike, et al. after hexanediol treatment (UPDIKE *et al.* 2011) and Smith, et al. after embryo rupture (SMITH *et al.* 2016). Thus, P granules dissipate without chemical crosslinking. We use fixation conditions that best capture assembled P granules, but despite this caution, some germline P granules may be more robust than others. Destabilized P granules may have released some mRNAs into the cytoplasm, which would lower the co-localization. Third, every reporter mRNA may not maintain PGL-1 association throughout its lifespan. We include these three possibilities in the text (lines 174-179).

The reviewer makes the keen observation that we detect reporter mRNAs localized to P granules in the absence of LambdaN22. We speculate mRNA colocalization with "wild-type" P granules may be due to two reasons. First, these mRNAs in P granules may be those exiting the nucleus. The Priess lab proposed that P granules exist on the cytoplasmic side of most, if not all, nuclear pores (Sheth, et al. (SHETH *et al.* 2010)). These overlapping reporter mRNAs may be newly transcribed mRNAs crossing through P granules to enter the cytoplasm. Second, and as the reviewer suggests, there may be endogenous mechanism(s) recruiting and/or trapping reporter mRNAs in P granules. If this is the case, it currently is unclear whether these mRNAs are being negatively regulated as they are when tethered to PGL-1. The revised manuscript now includes these possibilities in the Discussion (lines 179-185).

(4) For the P-granule localization of "mutated" PGL-1: Even after introduction of K126E and K129E substitutions or introduction of R123E substitution, these "mutated" PGL-1 proteins were still capable of localizing to P granules at least in a small fraction of gonads (Fig. S5E-H). Was this "minor" P-granule localization of "mutated" PGL-1 caused by an activity of another DD

domain, CDD, in PGL-1 or by a redundant activity of NtDD in PGL-3? Or does PGL-1 contain a third domain for dimerization? What are the speculations of the authors?

Response: We speculate that mutant PGL-1 can assemble with PGL-3 via the CDD and that PGL-3 still assembles into granules. Alternative, a third region may exist that associates with P granules. The revised manuscript now includes these possibilities in the text (lines 285-288, 379-384).

(5) For the protein level of "mutated" PGL-1: Assuming that all the images were taken under the same conditions, it appears to this reviewer that immunofluorescence levels of PGL-1::SNAP were also reduced after introduction of K126E K129E or R123E substitution into PGL-1::SNAP in multiple Figures (C vs. E, F, and H vs. M in Fig. 4; A, D vs. B, C in Fig. 6; A vs. C, D, graph E, and western blot F in Fig. S6). This reviewer requests the authors to quantify and compare the protein levels among PGL-1::SNAP, PGL-1(K126E K129E)::SNAP, and PGL-1(R123E)::SNAP in transgenic animals to answer this question. Possibly, one of the most straightforward approaches would be to reproduce a western immunoblot analysis as shown in Fig. S6F with addition of a protein sample obtained from PGL-1(K126E K129E)::SNAP animals. Then, band intensities of obtained immunoblots should be quantified, normalized, and statistically evaluated to answer this question. If reduction of PGL-1 protein levels after introduction of these substitutions is confirmed, how the authors would argue/explain this phenomenon? If these substitutions only affect dimerization or assembly of PGL-1, total protein level of PGL-1 in animals should not be changed. Recently, it was reported that ectopic expression of PGL proteins in somatic cells or a DNA damage-induced stress activated autophagy to degrade PGL proteins (Zhang et al, 2009, 2018; Min et al, 2019). Is there any possibility that the change of subcellular localization (diffusion to cytoplasm) or a structural change of PGL-1 caused by these substitutions (K126E K129E and R123E) might have induced autophagy to facilitate degradation of PGL-1?

Response: The idea of using immunoblots to compare protein levels suffers from the complication that germline sizes are variable within each strain and the ranges in size differ between the two key strains. Unfortunately, no ideal normalization control exists for germline proteins.

We agree that in situ fluorescence measurements are not perfect for quantitation of granular proteins – fluorescence has a tight linear range and protein concentrated in granules will have a dramatically higher signal than protein diffuse in the cytoplasm. Nonetheless, our fluorescent measurements (Figure S7E-G) suggest that PGL-1::SNAP fluorescence is lower in the assembly mutants than wild-type, as the reviewer noted. Possible explanations include enhanced protein turnover of mutant PGL-1 protein, as the reviewer suggests, but also effects of defective germ cell development and measurement inaccuracies. The revised manuscript now mentions these possibilities (lines 258-261).

(6) For the effect of "mutated" PGL-1 on the P-granule localization of PGL-3 (and GLH-1): As shown in Fig. 4N, O, P, not only PGL-1 but also the P-granule localization of PGL-3 was abolished by the K126E K129E substitution in PGL-1. (To this reviewer, it appears that the P-granule localization of GLH-1 was also, at least partially, abolished by this PGL-1 substitution like WAGO-1 in Fig. 6B, C.) Furthermore, it appears to this reviewer that protein levels of PGL-3 and GLH-1 were also reduced by this amino-acid substitution in PGL-1. Can the authors quantify and compare the protein levels of PGL-3 and GLH-1 between PGL-1::SNAP animals and PGL-1(K126E K129E)::SNAP animals using western blot analysis to make this point clearer? As authors mentioned in the manuscript, it was previously revealed that, although PGL-1 and PGL-3 could physically interact with each other, the absence of PGL-1 did not affect the localization of

PGL-3 to P granules. Furthermore, although there was no direct protein-protein interaction between PGL-1 and GLH-1, whereas localization of GLH-1 to P granules was not affected by the absence of PGL-1, localization of PGL-1 to P granules was totally abrogated by the absence of GLH-1 in C. elegans germ line (Kawasaki et al, 2004). Therefore, the effect of this amino-acid substitution in PGL-1 and the effect of the absence of PGL-1 on the P-granule localization of PGL-3 (and possibly GLH-1) are quite contrasting. Because this is an interesting new finding, this reviewer appreciates if the authors try to explain how these contrasting effects are generated by this amino-acid substitution in PGL-1 compared with the absence of PGL-1 in Discussion. One more question is, was the same "dominant-negative" effect observed when another substitution, R123E, was introduced into PGL-1 on the P-granule localization of PGL-3 (and GLH-1)?

Response: See response to comment (7).

(7) For the effect of PGL-1 amino-acid substitutions on WAGO-1 expression and localization: In animals expressing PGL-1(R123E)::SNAP + WAGO-1::V5, the WAGO-1::V5 expression was diffuse and its localization to P granules was almost abrogated in about half of the germlines (Fig. 6B, C). Did the authors observe similar change of WAGO-1::V5 localization pattern in animals expressing PGL-1(K126E K129E)::SNAP + WAGO-1::V5 compared with animals expressing PGL-1::SNAP + WAGO-1::V5? Furthermore, protein level of WAGO-1 also appears to be reduced at least in half of the animals expressing PGL-1(R123E)::SNAP + WAGO-1::V5 compared with animals expressing PGL-1::SNAP + WAGO-1::V5 (Fig. 6A vs. 6B). The authors should quantify and compare the protein level of WAGO-1 between PGL-1::SNAP animals and PGL-1(R123E)::SNAP animals using western blot analysis, as requested for the quantification of PGL-3 and GLH-1 in a previous comment. If reduction of WAGO-1 protein level in PGL-1(R123E)::SNAP animals is confirmed, this result indicates that assembly of PGL-1 to P granules is critical for both P-granule localization and maintenance of protein level of WAGO-1, both of which should contribute to repress expression of mRNA localized to P granules.

Response: The reviewer has a number of insightful questions about the relationships among PGL-1, PGL-3, GLH-1, and WAGO-1 but unfortunately, as discussed in response to comment (5), measuring protein levels is a challenge. Reagents for normalization will be needed to assay protein levels accurately and explore the relationships among PGL-1, PGL-3, GLH-1, and WAGO-1. The revised manuscript now mentions this future direction (lines 325-327). We did not test PGL-1 R123E::SNAP for its ability to act as a "dominant-negative" in the assembly of GLH-1 and PGL-3, but we should do so in the next manuscript while accurately examining granule protein levels by controlled immunoblot.

(8) By using their RNAi screening system, did the authors examine (or will the authors examine) in more "wider" scale, whether any of the other endo-siRNA pathway players (not listed in Fig. S7B), which possibly function in the same pathway as WAGO-1, such as players functioning in the 22G-RNA pathway, are also required for the repression of P-granule localized mRNA so as to support the "mRNA turnover" model?

Response: We agree that the targeted RNAi screen for Argonautes should be expanded to include other RNAi pathway players to tease apart the PGL-1 mRNA repression mechanism more fully. The revised manuscript now mentions this idea as a future direction (lines 453-454).

Minor comments:
(1) Statistical significance of difference among values should be examined using a specified statistical method and should be addressed in Fig. S2N, S2O, S6F, and S9I.

Response: Statistical significance for numerical values are now included in the figures and figure captions (lines 997, 999-1000, 1026-1027, 1032, 1054-1055, 1075-1076, 1163, 1182-1185, 1229-1233, Figure S6A, Figure S7F-G, Figure S10E), and statistical methods described in Methods (lines 587, 649-650, 667-668, 681-684, 715-721).

(2) Why smFISH-SNAP overlap can exceed over 100% for wago-1 wild type in the Fig. S9I box plot?

Response: The greater than 100% overlap reflects how Imaris software calculates surface area. The revised manuscript now includes a supplemental figure (Fig S9L), corresponding caption, and details in the Method that explains this discrepancy (lines 737-740).

(3) The description on line 119, "PGL-1::SNAP with λN22 had GFP was faintly detected in only a few gonads" appears grammatically incorrect. At least, this reviewer could not understand the meaning well. Is this sentence means, for example, "In PGL-1::SNAP::λN22 animals, GFP was faintly detected in only a few gonads"? Please correct this sentence.

Response: We have corrected the sentence in the Results (lines 143-144).

REVIEWER 2

The manuscript by Aoki et al. describes the identification of a second dimerization interface in PGL-1, and further tries to illustrate that mutations affecting dimerization alter PGL assembly and function.

This manuscript builds on prior work from the lab demonstrating the identification of a second dimerization domain (DD) in PGL proteins. Thus, PGL-1 and -3 contain not one, but two dimerization domains that appear to be essential for their assembly into P-granules.

Interestingly, among the four molecules in the asu, two interfaces were identified, though only one is reported to involve "conserved" amino acids. Although it is appreciated that residues involved in the NtDD interface are listed in Figure S4, it is common practice to report on buried surface area of all interfaces identified in the crystal structure, as was done by the authors in their 2016 publication in PNAS. It would also be helpful if the authors discussed how they believe the three mutations alter dimerization. If I am reading this correctly, the three residues are not listed as being involved in either hydrogen bonds or salt bridges, yet they are potent in their ability to disrupt dimerization, at least in the case of PGL-3 (Figure S4C). Is this through simple steric clashes or electronic repulsion?

Response: The revised manuscript now reports the buried surface area for both interfaces in the caption (Figure 2 (lines 1005-1008)), buried surface area of tested interface = 874.1 Å^2). We apologize for that omission along with the confusion in Figure S5D. The mutated residues are conserved among all *Caenorhabditid* species. The originally submitted Figure S5D reported predicted contacts in the *C. japonica* NtDD crystal structure but with *C. elegans* PGL-1 numbering. The revised manuscript now includes salt bridge and hydrogen bond residues that are predicted for the *C. japonica* NtDD crystal structure and the corresponding residues for *C. elegans* PGL-1 in parentheses (Figure S5D, lines 1147-1150).

For clarity, it would be helpful if more information were provided concerning the alignment of residues. The rmsd values are very low but it's unclear from what atoms this value was derived

(all atom or alpha carbon), what residues (authors indicate not termini and loops-not clear what that does include?), and if refinement included symmetry constraints or not, and if so, on what residues. It would also be helpful if primary colors were used for distinguishing subunits. It is very difficult to clarify subunits in some images (Figure 2A, right panel).

Response: We apologize for the confusion. Amino acid G114 and the first 3 residues of the N-terminus could not be visualized in all of the copies of the ASU. The RMSD was calculated with only the C-alpha carbons present in all four ASU copies. No symmetry constraints were used in refinement. The revised manuscript includes these points in the Results and Methods (lines 209-211, 549-552). As recommended, we have also changed the subunit colors in Figure 2A (lines 1006-1007).

It's unclear why mutations are being biochemically evaluated in PGL-3 while using PGL-1 as a model, as defined by the crystal structure. Ideally one should compare apples to apples and truly examine what is observed in the structure. Although I understand the two proteins share significant homology, since little information is given, an important question remains about the additional interface observed in the structure, which may or may not be present in PGL-3.

Response: These comments are fair and appreciated. We previously had trouble expressing significant amounts of PGL-1 NtDD in bacteria, but were able to remedy this problem using a codon optimized expression construct (lines 499-500). Size Exclusion Chromatography with Multiangle Light Scattering (SEC-MALS) of PGL-1 wild type and mutant recombinant protein showed that wild type NtDD is a dimer and K126E K129E or R123E mutations disrupt dimerization (Figure 3D-F and Figure S5G, lines 215-216, 566-571, 1015-1021, 1154-1156). The revised manuscript includes this PGL-1 data in the main figures (Figure 3D-F and Figure S5G) and moves the PGL-3 data to the Supplement (Figure S5H-I).

References:

Brangwynne, C. P., C. R. Eckmann, D. S. Courson, A. Rybarska, C. Hoege *et al.*, 2009 Germline P granules are liquid droplets that localize by controlled dissolution/condensation. Science 324**:** 1729-1732.

Kawasaki, I., A. Amiri, Y. Fan, N. Meyer, S. Dunkelbarger *et al.*, 2004 The PGL family proteins associate with germ granules and function redundantly in *Caenorhabditis elegans* germline development. Genetics 167**:** 645-661.

Putnam, A., M. Cassani, J. Smith and G. Seydoux, 2019 A gel phase promotes condensation of liquid P granules in *Caenorhabditis elegans* embryos. Nat Struct Mol Biol 26**:** 220-226.

Saha, S., C. A. Weber, M. Nousch, O. Adame-Arana, C. Hoege *et al.*, 2016 Polar positioning of phase-separated liquid compartments in cells regulated by an mRNA competition mechanism. Cell 166**:** 1572-1584 e1516.

Sheth, U., J. Pitt, S. Dennis and J. R. Priess, 2010 Perinuclear P granules are the principal sites of mRNA export in adult C. elegans germ cells. Development 137**:** 1305-1314.

Smith, J., D. Calidas, H. Schmidt, T. Lu, D. Rasoloson *et al.*, 2016 Spatial patterning of P granules by RNA-induced phase separation of the intrinsically-disordered protein MEG-3. Elife 5**:** e21337.

Updike, D. L., S. J. Hachey, J. Kreher and S. Strome, 2011 P granules extend the nuclear pore complex environment in the *C. elegans* germ line. J Cell Biol 192**:** 939-948.

Zhang, G., Z. Wang, Z. Du and H. Zhang, 2018 mTOR regulates phase separation of PGL granules to modulate their autophagic degradation. Cell 174**:** 1492-1506 e1422.

Reviewer #1 (Remarks to the Author):

I really appreciate the authors' tremendous efforts for revising the paper.
The authors responded to most of my comments as positively as they could.
All those revisions made by the authors are satisfactory to this reviewer. Therefore, I RECOMMEND ACCEPTING the revised manuscript for publishing in Nature Communications.

I found minor typos in the revised manuscript as follows. Therefore, please proof them:

Typo on the line 82 "similarity, (STOTHARD 2000))(KAWASAKI et al. 2004),".
Typo on the line 335 "The reporter dwas repressed".
Typo on the line 348 "smFISH signal was higher in wago-1 null mutants" (wago-1 should be italicized).
Typo on the line 454"additional factors necessary involved in regulating mRNA expression."
("necessary" and "involved" appear to be redundant).

Replies to REVIEWER 1 comments:

Reviewer #1 (Remarks to the Author):

I really appreciate the authors' tremendous efforts for revising the paper.
The authors responded to most of my comments as positively as they could.
All those revisions made by the authors are satisfactory to this reviewer. Therefore, I RECOMMEND ACCEPTING the revised manuscript for publishing in Nature Communications.

I found minor typos in the revised manuscript as follows. Therefore, please proof them:

Typo on the line 82 “similarity, (STOTHARD 2000))(KAWASAKI et al. 2004),”.

Response: The typo was corrected.

Typo on the line 335 “The reporter dwas repressed”.

Response: The typo was corrected.

Typo on the line 348 “smFISH signal was higher in wago-1 null mutants” (wago-1 should be italicized).

Response: The typo was corrected.

Typo on the line 454“additional factors necessary involved in regulating mRNA expression.” (“necessary” and “involved” appear to be redundant).

Response: The typo was corrected.
